# Comparative analysis of multimodal large language models GPT-4o and o1 versus clinicians in clinical case challenge questions: Retrospective cross-sectional study

**DOI:** 10.1097/MD.0000000000047071

**Published:** 2026-01-23

**Authors:** Jaewon Jung, Hyunjae Kim, SungA Bae, Jin Young Park

**Affiliations:** a Department of Medicine, Yonsei University College of Medicine, Seoul, Republic of Korea; b Department of Biomedical Systems Informatics, Yonsei University College of Medicine, Yongin, Republic of Korea; c Department of Internal Medicine, Division of Cardiology, Yonsei University College of Medicine, Yongin Severance Hospital, Yongin, Republic of Korea; d Department of Psychiatry, Yongin Severance Hospital, Yonsei University College of Medicine, Yongin, Republic of Korea.

**Keywords:** artificial intelligence, clinical decision-making, diagnostic accuracy, GPT-4 omni, multimodal large language model, o1

## Abstract

Generative pretrained transformer 4 (GPT-4) has demonstrated strong performance in standardized medical examinations but has limitations in real-world clinical settings. To address these limitations, the multimodal GPT-4o model integrates text and image inputs, and the multimodal o1 model incorporates advanced reasoning. This study compared the performance of GPT-4o and o1 against that of Medscape respondents (majority vote) in real-world clinical case challenges. This retrospective, cross-sectional study used 1426 Medscape case challenge questions from May 2011 to June 2024. Each case included text and images of patient history, physical examinations, diagnostic tests, and imaging studies. Medscape respondents were required to choose 1 answer from among multiple options, with the most frequent response defined as the Medscape respondent’s decision. GPT models (3.5 Turbo, 4 Turbo, 4 Omni, and o1) were used to interpret the text and images and generate formatted responses. We compared the performances of the Medscape respondents and GPT models using mixed-effects logistic regression analysis. Medscape respondents (majority vote) achieved an overall accuracy of 85.0%, whereas GPT-4o and o1 demonstrated higher accuracies of 88.4% (*P* = .005) and 94.3% (*P* < .001), respectively. In multimodal analysis involving images (n = 917), GPT-4o achieved an accuracy of 88.3% (*P* = .005), while o1 achieved 93.9% (*P* < .001), both significantly outperforming Medscape respondents. o1 demonstrated the highest accuracy across all question categories, achieving 92.6% (*P < *.001) in diagnosis, 97.0% (*P* < .001) in disease characteristics, 92.6% (*P* = .002) in examination, and 94.8% (*P* = .005) in treatment. In terms of medical specialty, o1 achieved 93.6% (*P* < .001) accuracy in internal medicine, 96.6% (*P* = .030) in major surgery, 97.3% (*P* = .030) in psychiatry, and 95.4% (*P* < .001) in minor specialties, significantly surpassing Medscape respondents. Across 5 trials, GPT-4o and o1 provided the correct answer 5/5 times in 86.2% and 90.7% of the cases, respectively. The GPT-4o and o1 models achieved higher accuracy than Medscape respondents (majority vote) in clinical case evaluations, particularly in disease diagnosis. GPT-4o and o1 could serve as valuable tools to assist healthcare professionals in structured scenarios.

## 1. Introduction

Rapid advancements in artificial intelligence (AI) have revolutionized various sectors, including healthcare, where the importance of digital health technologies is increasingly emphasized.^[1–3]^ Of these technologies, large language models (LLMs) have shown considerable promise in healthcare, particularly in answering medical questions, aiding diagnoses, and automating administrative tasks.^[4]^ Generative pretrained Transformer 4 (GPT-4; OpenAI, San Francisco, CA, USA) is one of the most advanced LLMs, consistently achieving high scores on the United States Medical Licensing Examination and often surpassing the passing threshold.^[5–7]^ Despite these achievements, existing evaluations have predominantly focused on text-only formats, which frequently exclude or substitute visual elements with descriptions. This limitation reduces the applicability of GPT-4 in image-dependent specialties such as radiology.

To address these limitations of unimodal LLMs, the newly released GPT-4 Omni introduces multimodal capabilities, enabling it to process and integrate both textual and visual information.^[8]^ This enhancement allows for a more comprehensive analysis of clinical cases, particularly in fields such as diagnostic and interventional radiology and nuclear medicine, where early studies have demonstrated its potential.^[9,10]^ Despite these advancements, existing research has focused largely on simplified exam questions, and no studies have reported significant performance improvements over text-only models in medical examinations.^[11,12]^ This gap highlights the need to investigate the performance of such multimodal models in clinical case settings that reflect the complexity of diagnostic decision-making encountered in clinical practice.

In parallel with these multimodal developments, OpenAI recently introduced the o1 model, which incorporates advanced reasoning capabilities designed to improve safety and robustness.^[13]^ By utilizing chain-of-thought reasoning, o1 has demonstrated better adherence to safety protocols in complex scenarios, effectively reducing the risk of generating harmful or biased content. These enhanced reasoning capabilities are particularly crucial in healthcare, where complex problem-solving and data interpretation are vital.^[14]^ Several studies have demonstrated that o1 outperforms both GPT-4 and GPT-4o models in clinical settings, underscoring its superior reasoning abilities in medical decision-making processes.^[15,16]^ However, the performance of the o1 model in complex real-world clinical cases remains unexplored owing to its recent release.

Accordingly, this study evaluated the clinical potential of 2 advanced LLMs – GPT-4 Omni and o1 – in solving complex clinical case challenge questions that incorporate both textual and visual data and validated their effectiveness as diagnostic support tools in comparison with clinicians.

## 2. Methods

### 2.1. Study design

**Figure 1.**
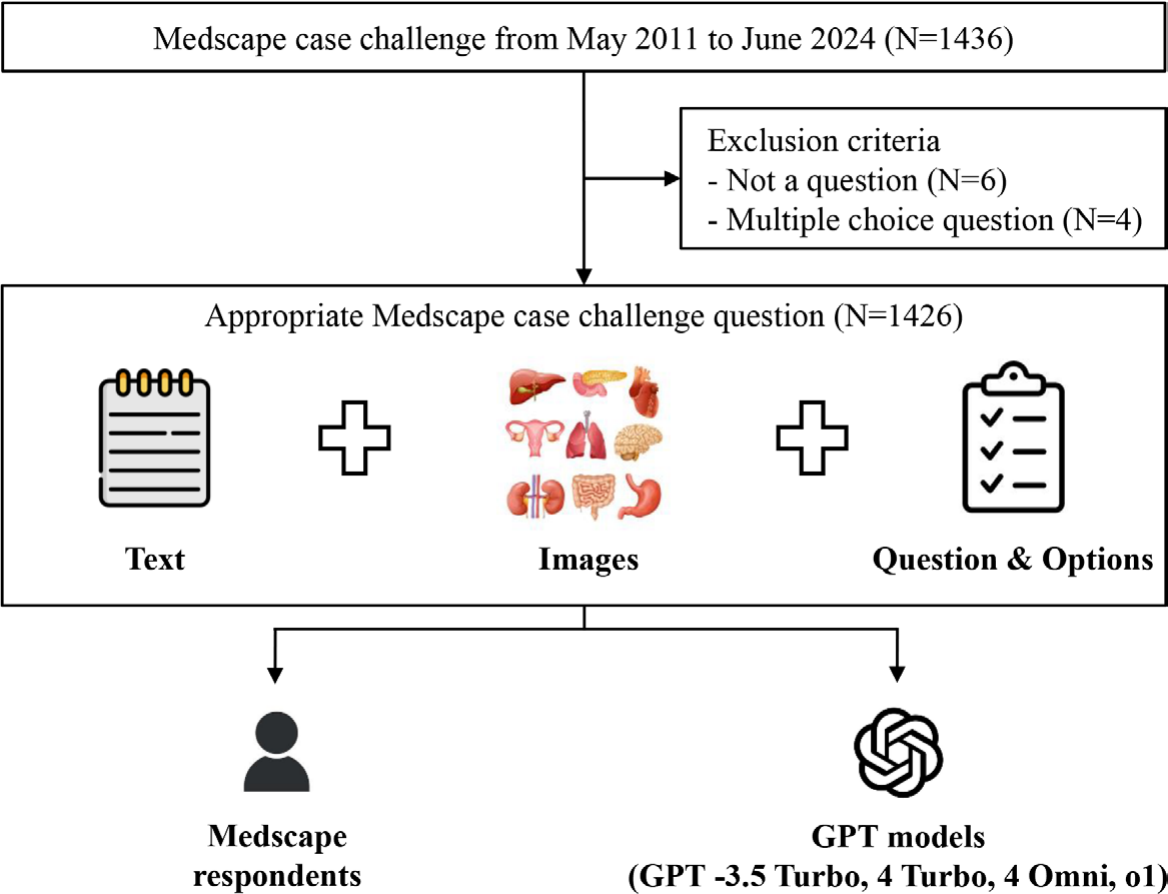
Study flowchart. This flowchart illustrates the workflow for evaluating the diagnostic performances of GPT models compared with Medscape respondents (majority vote) in Medscape case challenge questions. GPT = generative pretrained transformer.

This retrospective study analyzed Medscape clinical case challenge questions from May 2011 to June 2024 (Fig. 1, Fig. S1, Supplemental Digital Content, and Table S1, Supplemental Digital Content, https://links.lww.com/MD/R100).^[17]^ Medscape clinical case challenges are interactive educational tools designed to help healthcare professionals assess and improve their clinical knowledge and decision-making skills. Only publicly accessible cases were included, and no personal data nor protected health information were collected. Ethical approval was not required owing to the retrospective nature of the study and the use of open-source data. This study was conducted and reported in accordance with the STROBE (Strengthening the Reporting of Observational Studies in Epidemiology) guidelines.

Each case presented a realistic clinical scenario, including patient history, physical examination findings, diagnostic test results, and imaging studies. Participants were tasked with selecting the most appropriate answer from a set of multiple-choice options, covering aspects of diagnosis, disease characteristics, examinations, and treatment strategies. Clinicians’ responses were defined as the most frequently selected option for each question. Each question had a predefined correct answer, established by the Medscape case author based on the confirmed diagnosis and clinical guidelines. The cases were categorized by medical specialty, including internal medicine (allergy, cardiology, endocrinology, gastroenterology, hematology, infectious diseases, nephrology, oncology, pulmonology, and rheumatology), major surgery (obstetrics/gynecology and surgery), pediatrics, psychiatry, and minor specialties (dermatology, emergency medicine, neurology, neurosurgery, ophthalmology, orthopedics, otorhinolaryngology, and urology).

#### 2.1.1. Data Governance

Clinical case data were collected from publicly accessible Medscape pages using a custom Python-based scraper. The cases were preselected from a dedicated clinical challenge section of the website. Only open-access content was retrieved without bypassing any authentication or restrictions. All data were stored in a secure, access-controlled environment, and no patient-identifiable information was involved at any stage of data processing or analysis.

#### 2.1.2. Large language models and prompts

The models evaluated included GPT-3.5 Turbo (March 2023), GPT-4 Turbo (January 2024), GPT 4o (May 2024), and o1 (September 2024). Each GPT model was accessed through the OpenAI application programming interface without browsing or tool functionalities enabled. They were tasked to generate a formatted response based on the available options for each question. To ensure consistency and replicability, the models were queried using predefined prompt templates that simulated a clinical reasoning scenario. All prompts were submitted in batch mode using manually defined inputs, and no retry logic was implemented during the evaluation process. Table 1 outlines the prompts used to query the models, including their structures and formatting. For questions that included images, both textual and visual data were provided to the models where applicable, enabling multimodal input processing in GPT-4o and o1. Original images uploaded by Medscape authors were used in JPG format without any preprocessing. Captions of images were included in text inputs when present. The chat session was reset before each new question to eliminate the influence of memory retention or in-context learning, ensuring independent processing and unbiased responses. To prevent potential data leakage, identifiable elements including case titles, URLs, publication dates, and author names were removed from every prompt prior to model input. GPT-3.5 Turbo, GPT-4 Turbo, and GPT-4 Omni were configured with a temperature of 0.1 and top-p of 1.0, while o1 maintained its default settings of a temperature of 1.0 and top-p of 1.0.

**Table 1:** Prompt template for GPT models.

Models	Prompt
GPT-3.5 Turbo, GPT-4 Turbo	You are a specialist clinician. The data provided is about 1 patient, and it might involve a rare disease, so consider many possibilities. As a clinician, you must make data-based decisions, highly recommended to be based on journals and medical textbooks. Examination findings are very important!
	(Question)
	(Options)
	(All textual information)
GPT-4 Omni, o1	You are a specialist clinician. The data provided is about 1 patient, and it might involve a rare disease, so consider many possibilities. As a clinician, you must make data-based decisions, highly recommended to be based on journals and medical textbooks. Examination findings are very important!
	(Question)
	(Options)
	(All textual information)
	(Images)

#### 2.1.3. Statistical analysis

We compared the performances of the 4 GPT models (GPT-3.5 Turbo, GPT-4 Turbo, GPT-4 Omni, and o1) with those of clinicians using mixed-effects logistic regression analysis to evaluate differences in accuracy. Model type was included as a fixed effect to compare performance across models, and a random intercept was added for each case to account for potential correlations among nested questions within the same case. We calculated the accuracy and 95% confidence intervals for each model and clinician and made pairwise comparisons between them. For cases with images, a focused analysis of GPT-4o and o1 was conducted to specifically assess their multimodal capability in interpreting visual data. The dataset was further analyzed by question category (diagnosis, characteristics, examination, and treatment), medical specialty (internal medicine, major surgery, pediatrics, psychiatry, and minor specialties), option counts (2–3, 4,5, ≥ 6), and image modality (radiography, CT, sonography, MRI, pathology images, cutaneous/external findings, ECG, endoscopy, angiography, others). GPT-4o and o1 were tested repeatedly using the same set of questions to evaluate response consistency, and a multilevel logistic mixed model was used to quantify intraclass correlation coefficient, accounting for potential within-case correlations. All analyses were conducted using R (version 4.5.0, R Foundation for Statistical Computing, Vienna, Austria), with a *P*-value < .05 considered statistically significant.

## 3. Results

### Question characteristics

We evaluated the performance of 4 language models – GPT-3.5 Turbo, GPT-4 Turbo, GPT-4 Omni, and o1 – using a dataset of 1426 cases. Of these, 917 questions included images, resulting in a total of 1475 images. Figure S2, Supplemental Digital Content, https://links.lww.com/MD/R100 displays the quiz samples. The number of answer options per question ranged from 2 to 9, with the majority of cases (97.2%) offering either 4 or 5 answer options – 906 cases had 4 options, and 480 had 5. The random chance of selecting the correct answer was 23.1%.

### Overall accuracy and model comparison

**Figure 2.**
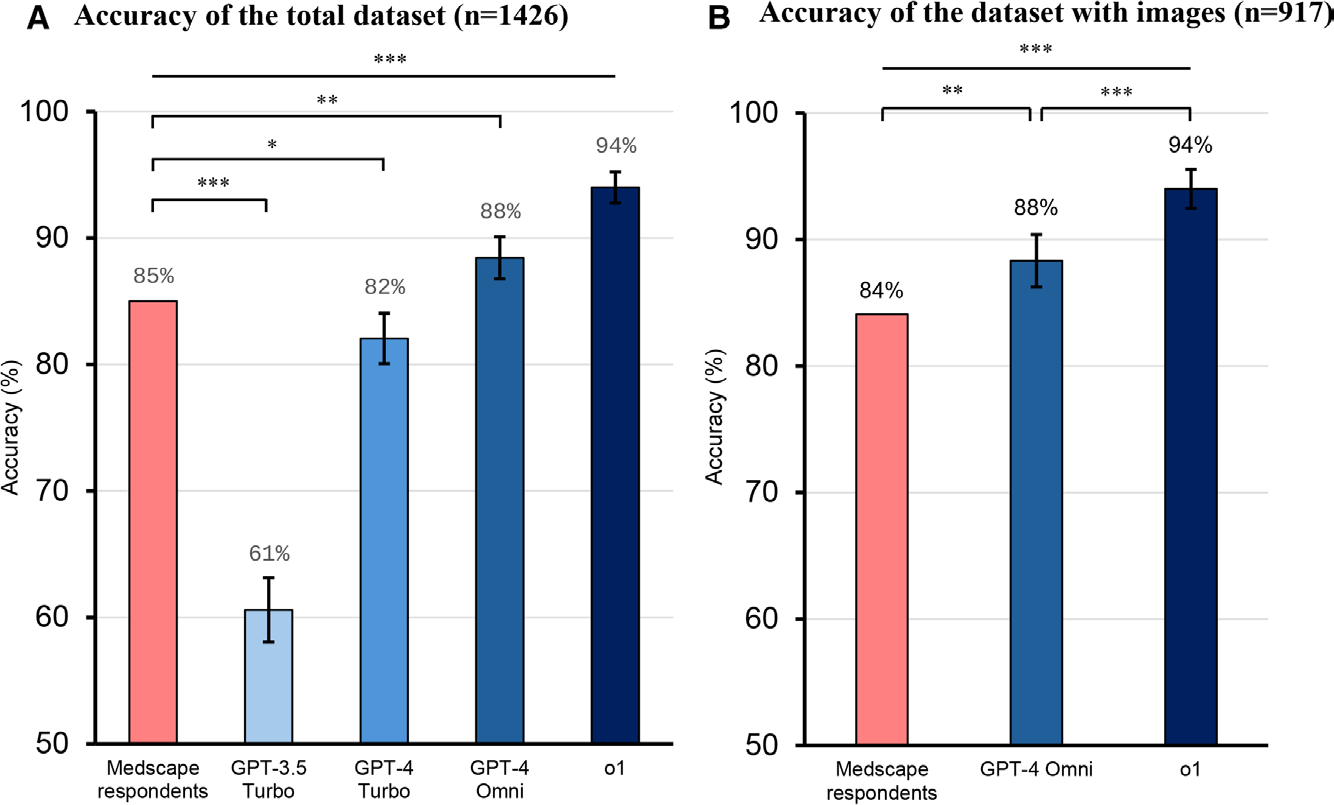
Overall performance of the GPT models. (A) Accuracy of the models across the entire dataset (n = 1426). Medscape respondents (majority vote) achieved an accuracy of 85.0%, while GPT-3.5 Turbo, GPT-4 Turbo, GPT-4 Omni, and o1 achieved accuracies of 60.6%, 82.1%, 88.4%, and 94.3%, respectively. Statistical comparisons showed that GPT-4o and o1 significantly outperformed Medscape respondents (**P <.01; ***P <.001), while GPT-3.5 Turbo and GPT-4 Turbo performed significantly worse (***P <.001; *P <.05). Error bars represent 95% confidence intervals. (B) Accuracy of models for the dataset subset with images (n = 917). Medscape respondents (majority vote) achieved an accuracy of 84.1%, while GPT-4o achieved 88.3%, significantly surpassing Medscape respondents (**P <.01). o1 demonstrated an accuracy of 93.9%, significantly outperforming both Medscape respondents and GPT-4o (***P <.001 for both). Error bars represent 95% confidence intervals. GPT = generative pretrained transformer.

Medscape respondents (majority vote) demonstrated an accuracy of 85.0% across the entire dataset (see Fig. 2A and Table S2, Supplemental Digital Content, https://links.lww.com/MD/R100). In comparison, GPT-3.5 Turbo, GPT-4 Turbo, GPT-4o, and o1 achieved accuracies of 60.6% (95% CI, 58.1–63.1), 82.1% (95% CI, 80.1–84.0), 88.4% (95% CI, 86.8–90.1), and 94.3% (95% CI, 93.1–95.5), respectively. The performances of GPT-4o and o1 surpassed that of Medscape respondents, demonstrating significantly higher accuracy (*P* = .005 and *P < *.001, respectively). By contrast, GPT-3.5 Turbo and GPT-4 Turbo performed significantly lower than Medscape respondents (*P* < .001 and *P* = .028, respectively).

**Figure 3.**
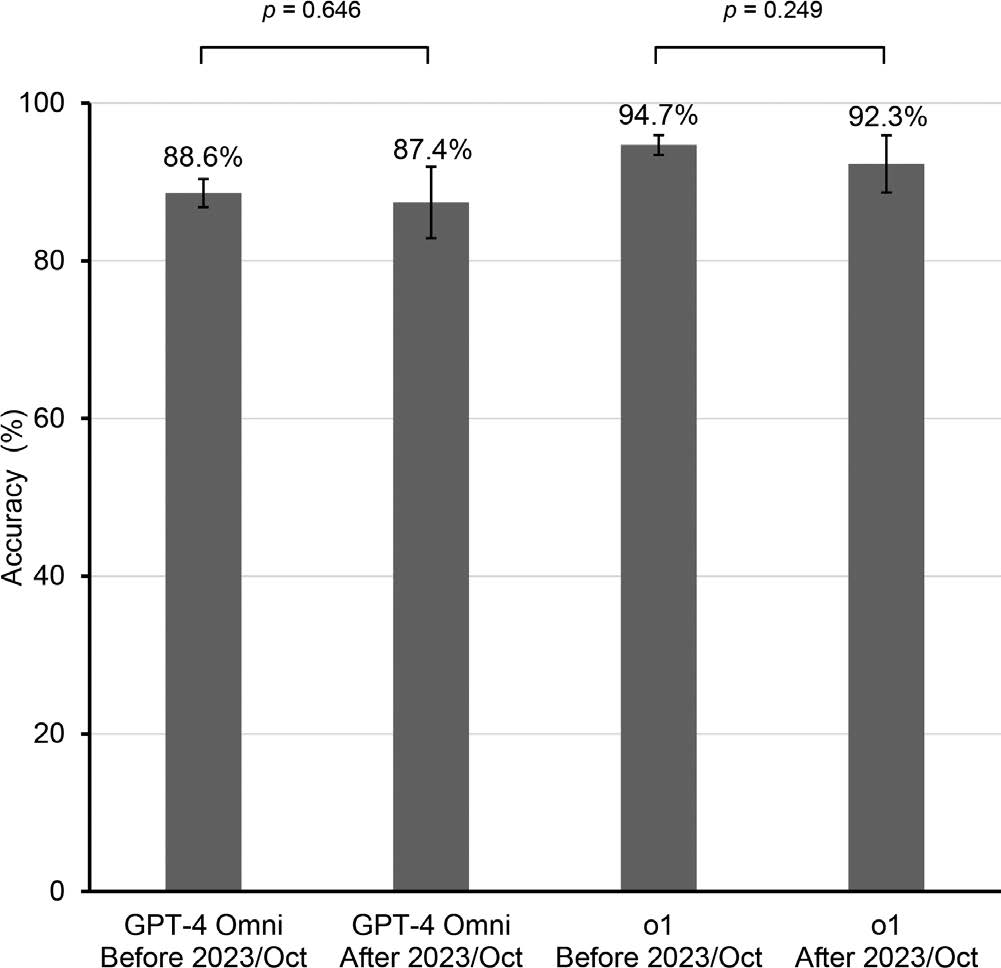
Model performances before and after the knowledge cutoff date. Accuracy of GPT-4o and o1 on clinical case datasets introduced before (n = 1219) and after (n = 207) the October 2023 knowledge cutoff. Both models demonstrated comparable accuracies across the 2 periods (*P* = .646 for GPT-4o and *P* = .249 for o1), suggesting minimal to no effect of potential data exposure on model performance. Error bars indicate 95% confidence intervals. GPT = generative pretrained transformer.

To assess the potential impact of data exposure on model performance, we evaluated the accuracy of GPT-4o and o1 on datasets introduced both before (n = 1219) and after (n = 207) the knowledge cutoff date of October 2023. Both models demonstrated comparable accuracy across the 2 time periods (*P* = .646 for GPT-4o and *P* = .249 for o1), indicating that exposure to the test data had minimal to no impact on model accuracies (Fig. 3).

### Multimodal model performances on cases with images

In the subset of cases that included images (n = 917), Medscape respondents (majority vote) achieved an accuracy of 84.1% (Fig. 2B). GPT-4o performed better, with an accuracy of 88.3% (95% CI, 86.3–90.4), significantly higher than that of Medscape respondents (*P* = .005). o1 exhibited the highest performance, achieving an accuracy of 93.9% (95% CI, 92.3–95.4), significantly surpassing those of both Medscape respondents and GPT-4o (*P* < .001 for both).

### Performance by question category

The dataset was stratified based on question category, with the diagnosis category comprising the largest number of questions (n = 530), followed by disease characteristics (n = 401), treatment (n = 305), and examination (n = 190) (Table 2). o1 demonstrated the highest accuracy across all question categories, consistently outperforming Medscape respondents (majority vote) and other GPT models. Overall, it achieved 94.3% accuracy, significantly surpassing Medscape respondents (85.0%, *P* < .001) and all other models.

**Table 2:** Performance by question category.

Category	No. of questions	No. of options (median)	Accuracy (95% CI, n)					Odds ratio*	Odds ratio†	*P*-value‡	*P*-value§
			Medscape respondents	GPT							
				3.5 Turbo	4 Turbo	4 Omni	o1				
Diagnosis	530	4	78.1 (74.6–81.6, 414)	56.4 (52.2–60.6, 299)	76.8 (73.2–80.4, 407)	84.9 (81.9–88.0, 450)	92.6 (90.4–94.9, 491)	1.80 (1.25–2.59	4.51 (2.86–7.11)	**.002**	**<.001**
Disease characteristics	401	4	89.8 (86.8–92.7, 360)	62.6 (57.9–67.3, 251)	86.5 (83.2–90.0, 347)	91.5 (88.8–94.2, 367)	97.0 (95.3–98.7, 389)	1.45 (0.76–2.79)	18.14 (5.65–58.24)	.261	**<.001**
Examination	190	4	85.3 (80.2–90.3, 162)	65.8 (59.0–72.5, 125)	85.8 (80.8–90.8, 163)	89.5 (85.1–93.8, 170)	92.6 (88.9–96.3, 176)	2.17 (0.85–5.53)	8.74 (2.26–33.73)	.105	**.002**
Treatment	305	4	90.5 (87.2–93.8, 276)	62.0 (56.5–67.4, 189)	83.0 (78.7–87.2, 253)	89.8 (86.4–93.2, 274)	94.8 (92.3–97.3, 289)	0.87 (0.43–1.79)	4.39 (1.56–12.33)	.715	**.005**
Total	1426	4	85.0 (83.1–86.8, 1212)	60.6 (58.1–63.1, 864)	82.1 (80.1–84.0, 1170)	88.4 (86.8–90.1, 1261)	94.3 (93.1–95.5, 1345)	1.39 (1.10–1.74)	3.20 (2.42–4.23)	**.005**	**<.001**

Bold values indicate statistical significance (*P* < .05).

CI = confidence interval, GPT = generative pretrained transformer.

* 4o relative to Medscape respondents.

† o1 relative to Medscape respondents.

‡ Medscape respondents versus 4o.

§ Medscape respondents versus o1.

In the diagnosis and disease characteristics categories, o1 achieved accuracies of 92.6% and 97.0%, respectively, significantly outperforming Medscape respondents (*P* < .001 for both). In the examination category, o1 achieved 92.6% accuracy, surpassing Medscape respondents, who achieved 85.3% accuracy (*P* = .002). In the treatment category, o1 also outperformed Medscape respondents, achieving 94.8% accuracy compared to 90.5% for Medscape respondents (*P* = .005).

In the diagnosis category, GPT-4o significantly outperformed Medscape respondents, achieving 84.9% accuracy compared to 78.1% for Medscape respondents (*P* = .002). For categories related to disease characteristics and examination, GPT-4o also demonstrated higher accuracy than Medscape respondents; however, the differences were not statistically significant (*P* = .261 and *P* = .105, respectively). In the treatment category, Medscape respondents showed greater accuracy than GPT-4o, but the difference was not statistically significant (*P* = .715).

### Performance by medical specialty

The dataset was stratified by medical specialty, with internal medicine comprising 766 questions, followed by minor specialties (n = 390), pediatrics (n = 108), major surgery (n = 87), and psychiatry (n = 75) (Table 3).

**Table 3:** Performance by medical specialty.

Category	No. of questions	No. of options (median)	Accuracy (95% CI, n)					Odds ratio*	Odds ratio†	*P*-value‡	*P*-value§
			Medscape respondents	GPT							
				3.5 Turbo	4 Turbo	4 Omni	o1				
Internal medicine	766	4	83.3 (80.6–85.9, 638)	59.0 (55.5–62.5, 452)	80.2 (77.3–83.0, 614)	86.9 (84.6–89.3, 666)	93.6 (91.9–95.3, 717)	1.37 (1.02–1.84)	3.22 (2.24–4.64)	**.037**	**<.001**
Major surgery	87	4	87.4 (80.4–94.3, 76)	64.4 (54.3–74.4, 56)	79.3 (70.8–87.8, 69)	88.5 (81.8–95.2, 77)	96.6 (92.7–100.0, 84)	1.13 (0.42–3.04)	4.62 (1.16–18.46)	.802	**.030**
Pediatrics	108	4	92.6 (87.7–97.5, 100)	61.1 (51.9–70.3, 66)	84.3 (77.4–91.1, 91)	90.7 (85.3–96.2, 98)	91.7 (86.5–96.9, 99)	0.78 (0.29–2.09)	0.88 (0.32–2.39)	.617	.798
Psychiatry	75	4	86.7 (79.0–94.4, 65)	60.0 (48.9–71.1, 45)	82.7 (74.1–91.2, 62)	84.0 (75.7–92.3, 63)	97.3 (93.7–100.0, 73)	0.81 (0.33–2.00)	5.62 (1.19–26.58)	.645	**.030**
Minor	390	4	85.4 (81.9–88.9, 333)	62.8 (58.0–67.6, 245)	85.6 (82.2–89.1, 334)	91.5 (88.8–94.3, 357)	95.4 (93.3–97.5, 372)	2.00 (1.23–3.23)	4.03 (2.25–7.23)	**.005**	**<.001**
Total	1426	4	85.0 (83.1–86.8, 1212)	60.6 (58.1–63.1, 864)	82.1 (80.1–84.0, 1170)	88.4 (86.8–90.1, 1261)	94.3 (93.1–95.5, 1345)	1.39 (1.10–1.74)	3.20 (2.42–4.23)	**.005**	**<.001**

Bold values indicate statistical significance (*P* < .05).

CI = confidence interval, GPT = generative pretrained transformer.

* 4o relative to Medscape respondents.

† o1 relative to Medscape respondents.

‡ Medscape respondents versus 4o.

§ Medscape respondents versus o1.

o1 achieved an accuracy of 93.6% in internal medicine and 95.4% in minor specialties, which were statistically significant compared to Medscape respondents (majority vote) (*P* <.001 for both). In major surgery, o1 outperformed Medscape respondents with 96.6% accuracy, while Medscape respondents achieved 87.4% accuracy (*P* = .030). In psychiatry, o1 achieved an accuracy of 97.3%, surpassing Medscape respondents, who achieved 86.7% accuracy (*P* = .030). Medscape respondents demonstrated numerically higher performance in pediatrics than o1, but the difference was not statistically significant (*P* = .798).

GPT-4o demonstrated superior accuracy in internal medicine, with 86.9% compared to 83.3% for Medscape respondents (*P* = .037). Similarly, in minor specialties, GPT-4o outperformed Medscape respondents, achieving 91.5% accuracy compared to 85.4% (*P* = .005). In addition, GPT-4o also showed higher accuracy than that of Medscape respondents in major surgeries; however, the difference was not statistically significant (*P* = .802). By contrast, Medscape respondents demonstrated higher accuracy in pediatrics and psychiatry, but these differences were not statistically significant (*P* = .617 and *P* = .645, respectively).

### Performance by option count and image modality

The dataset was further stratified by option count and image modality. Across different numbers of answer options, the overall trend was consistent, with o1 achieving the highest accuracy, followed by GPT-4o and Medscape respondents (Table S3, Supplemental Digital Content, https://links.lww.com/MD/R100). No significant differences were observed according to the presence or type of image modality (Table S4, Supplemental Digital Content, https://links.lww.com/MD/R100).

### Consistency of responses

**Figure 4.**
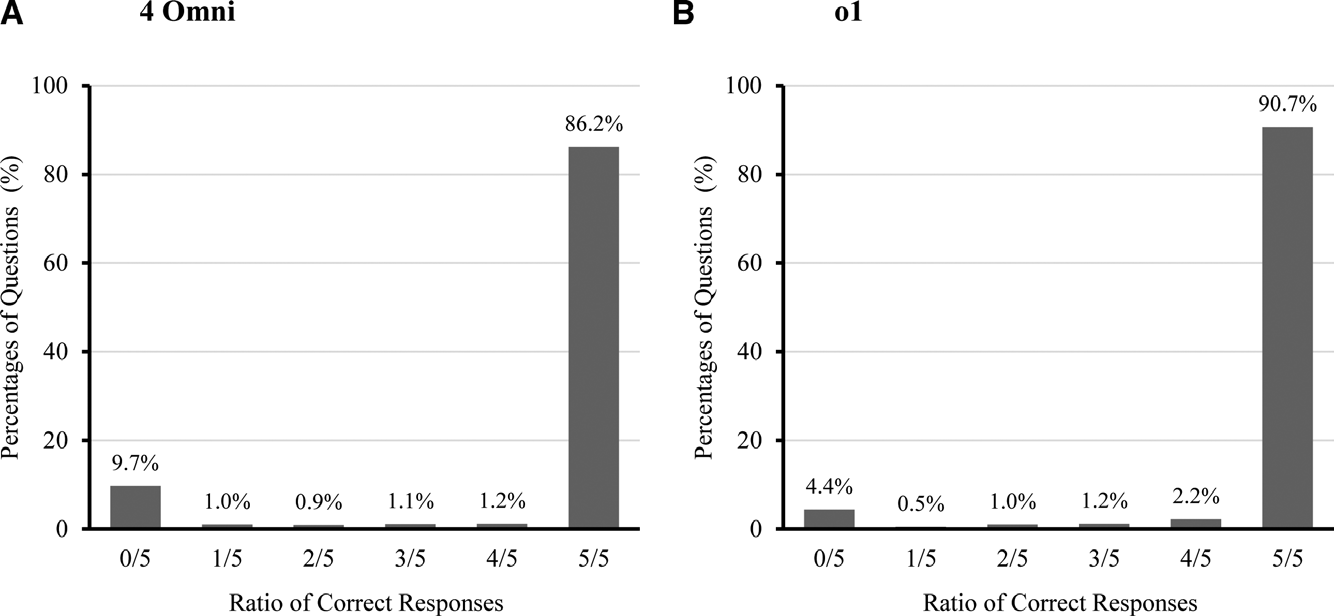
Response consistency of GPT-4o and o1. (A) Performance of GPT-4o across 5 independent trials, showing that 86.2% of cases had all correct responses (5/5). GPT-4o achieved at least 1 correct response in 90.3% of cases across the trials. (B) Performance of o1 across 5 independent trials, demonstrating slightly higher consistency, with 90.7% of cases achieving all correct responses (5/5). o1 achieved at least 1 correct response in 95.6% of cases across the trials. GPT = generative pretrained transformer.

GPT-4o and o1 were evaluated using the entire dataset over 5 independent trials to assess response consistency (Fig. 4). GPT-4o achieved the correct answer 5/5 times in 86.2% of the cases, while o1 demonstrated slightly higher consistency, with the correct answer 5/5 times in 90.7% of the cases. Notably, GPT-4o answered correctly at least once in 90.3% of the cases across the 5 trials, whereas o1 achieved at least 1 correct answer in 95.6% of cases. The inter-run agreement was substantial for the GPT-4o model (ICC = 0.760) and almost perfect for the o1 model (ICC = 0.881), indicating strong and highly reproducible predictions across independent runs.

## 4. Discussion

### Principal results

This study demonstrates that recent GPT models, specifically o1 and GPT-4o, can outperform clinicians in clinical case challenges.

### Comparison with prior work

Previous studies using earlier models, such as GPT-3.5 Turbo and GPT-4 Turbo, generally found that the results for medical cases were comparable to or below human performance.^[18,19]^ A prior study using Medscape clinical case challenges with a text-only model showed performance below 50%, highlighting significant limitations.^[20]^ By contrast, our study utilized multimodal models, resulting in substantial performance improvements. Both GPT-4o and o1 maintained high accuracy in cases involving images, indicating their ability to interpret various imaging types, including magnetic resonance imaging, computed tomography, radiography, ultrasound, and pathological images, across multiple specialties. These findings extend prior benchmarks that largely focused on text-only tasks and contribute to the growing literature on multimodal evaluation in real-world clinical scenarios. Additionally, earlier research showed that the o1 model outperformed the GPT-4 series in solving medical problems.^[14–16]^ Our findings further support this observation, as o1 outperformed GPT-4o in complex medical scenarios.

The accuracy of o1 remained above 90% across all question categories and medical specialties, with particular strength in diagnosis. The model also performed well in areas such as major surgery and psychiatry, with accuracy rates exceeding those of GPT-4o by more than 8%. Research suggests that the high performance of o1 is driven by its “chain-of-thought” reasoning, which systematically breaks down complex problems, reducing hallucinations and improving logical reasoning.^[13,14]^ This capability is particularly beneficial in clinical cases with abundant patient data, which often includes extraneous information. In such contexts, o1’s ability to filter relevant insights and synthesize data across specialties aids in understanding disease progression and managing complex multisystem conditions.

o1 demonstrated stronger performance and higher consistency than GPT-4o in selecting the most accurate option from multiple choices. This finding contrasts with previous studies suggesting similar gains between the o1 model and GPT-4 in probabilistic reasoning and critical diagnosis identification.^[21]^ This divergence may be attributed to differences in prompt design and parameter tuning, which allowed o1 to achieve significant improvements. However, this also suggests that o1’s performance may vary depending on the clinical setting.

### Integration into clinical workflows and application

**Figure 5.**
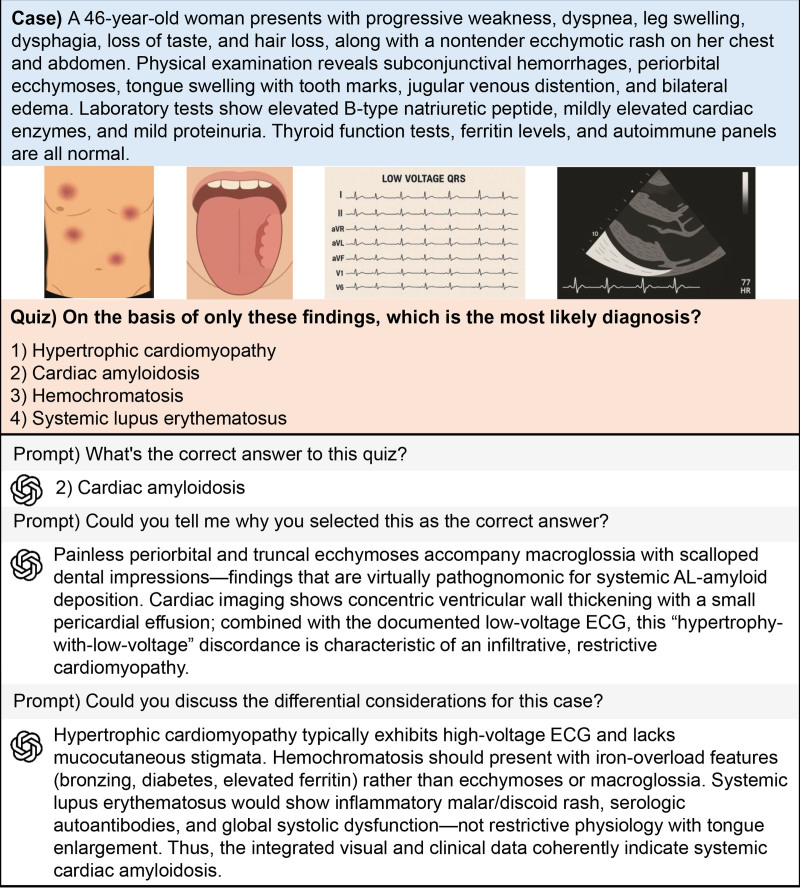
Illustrative pipeline of GPT applied to a clinical case. This figure demonstrates how the GPT model integrates textual and visual information to interpret a clinical case. The GPT model is prompted to identify the most likely diagnosis, explain its reasoning, and discuss differential diagnoses. The example consists solely of schematic/vector elements, with no patient-identifiable data included. GPT = generative pretrained transformer.

While the study demonstrates that AI models outperform clinicians in accuracy, their reasoning process remains a black box. Understanding how these models arrive at their conclusions and making this process interpretable for clinicians is essential for their integration into clinical workflows. In this context, Figure 5 illustrates how the AI model interprets imaging studies and synthesizes differential diagnoses. This approach allows clinicians to verify, understand, and critically assess AI recommendations, thus facilitating safer and more informed decision-making.

In practice, GPT models may serve as valuable tools to support clinicians, particularly in settings where specialized expertise is limited. Clinicians are prone to diagnostic errors and may occasionally order unnecessary tests, influenced by factors such as revenue considerations, while lacking sufficient tools to oversee such decisions.^[22–25]^ Recent models demonstrate potential to assist in differential diagnosis and initial treatment planning, particularly in fields outside a physician’s primary specialty.^[26]^ Furthermore, GPT models show promise in identifying rare diseases that might otherwise be overlooked and recommending appropriate referrals to specialized care.^[27]^ In medically underserved areas or environments where access to specialist consultation is restricted, AI systems could help reduce healthcare disparities by providing consistent decision support and improving diagnostic reach.^[28]^

### Remaining challenges and ethical considerations

Although AI demonstrates high diagnostic accuracy, the role of clinicians remains indispensable. The selection, preprocessing, and feeding of relevant patient information into AI systems rely heavily on clinician expertise.^[29]^ Moreover, while the models demonstrated impressive performance based on the presented case information, actual clinical settings are far more complex.^[30]^ Patients often provide incomplete histories or omit critical information, posing substantial challenges that current LLMs are not yet fully equipped to manage.^[31]^ Accurate model calibration and validation are therefore needed to ensure consistent performance across diverse clinical contexts. Further prospective studies using real-world clinical data, including free-form questions and open-ended scenarios, and examination of clinician–LLM interaction, are recommended to evaluate model robustness and generalizability.

As AI technologies, including large multimodal models capable of analyzing imaging data, become increasingly integrated into clinical workflows, new ethical considerations must be addressed. In addition to traditional concerns regarding accountability, transparency, and bias in algorithmic decision-making, the incorporation of sensitive medical images amplifies the importance of patient privacy and data protection.^[32]^ Furthermore, the establishment of clear legal frameworks is essential to delineate responsibility in cases where AI-assisted decisions lead to misdiagnosis.^[33]^ As substantial capabilities are demonstrated by these models, it remains crucial to recognize that AI systems currently lack the emotional understanding, empathy, and holistic grasp of individual patient cases that clinicians possess; therefore, the necessity of human intervention and interaction remains critical.

### Study limitations

Our study has several limitations. First, clinicians were defined as site voters rather than a standardized group of certified specialists, and the voting participants included a wide range of users. The lack of participant credential verification and the casual nature of answering, without academic or clinical consequences, may have contributed to the underestimation of actual clinician performance. While these limitations exist, the primary aim of the study was not to benchmark against definitive human performance but to illustrate the rapid and consistent progress of LLMs in performing medical tasks. Similar majority-vote baselines using non-clinician participants have been employed in previous studies to approximate medical decision-making. In prior work, such aggregated crowd-based accuracy has generally been comparable to that of certified physicians, supporting the validity of this approach as a scalable human benchmark.^[34]^ While not a substitute for expert evaluation, these methods have been accepted as practical proxies in diagnostic benchmarking.^[35]^ Given the observed performance trajectory, further studies involving board-certified clinicians are warranted to evaluate the models’ validity in real-world settings.

Second, the dataset was derived from Medscape clinical case challenges, which may not fully capture the diversity and complexity of real-world scenarios. Additionally, because the clinical case dataset was open source, the GPT models could have been exposed to these specific cases during training. To minimize this possibility, identifiers including case titles, URLs, publication dates, and author names were removed from prompts before model input. Importantly, o1 and GPT-4o were trained only on data available until October 2023, and their accuracy rates showed no statistically significant difference across datasets introduced before and after this date, suggesting that any prior exposure to the data had a limited impact on model accuracy.

Third, the dataset may have been influenced by inherent biases. This includes selection bias, as Medscape’s educational objectives often prioritized complex or rare conditions, leading to their preferential selection over common clinical presentations. In addition, latent biases, such as the overrepresentation of specific medical conditions, demographic groups, or clinical scenarios, may have limited the dataset’s generalizability and reduced its ability to reflect real-world patient diversity.^[36]^

Fourth, although decoding parameters were harmonized across GPT-3.5 Turbo, GPT-4 Turbo, and GPT-4 Omni, the o1 model was evaluated using its default decoding settings (temperature 1.0, top-p 1.0) due to architectural constraints and internal optimization. Further evaluation under fully standardized decoding conditions would help clarify performance differences more comprehensively.

## 5. Conclusions

The o1 and GPT-4o models achieved higher accuracy than clinicians in clinical case challenge questions, including those involving medical images. These findings suggest that both models can be valuable tools for healthcare professionals to support various aspects of patient care and decision-making in structured scenarios.

## Acknowledgments

The manuscript also underwent professional English language editing by Editage (www.editage.com). During the preparation of this manuscript, GPT-4o and o1 (OpenAI, 2025) were used as supplementary tools to assist with language polishing and vector image generation for figures. These tools were not used for data analysis, interpretation of results, or formulation of conclusions. After using this tool, the authors reviewed and edited the content as needed and take full responsibility for the content of the published article.

## Author contributions

**Conceptualization:** SungA Bae, Jin Young Park.

**Data curation:** Jaewon Jung, Hyunjae Kim.

**Formal analysis:** Jaewon Jung, Hyunjae Kim.

**Funding acquisition:** SungA Bae, Jin Young Park.

**Investigation:** Jaewon Jung, Hyunjae Kim.

**Methodology:** Hyunjae Kim.

**Software:** Jaewon Jung, Hyunjae Kim, SungA Bae.

**Supervision:** Jin Young Park.

**Validation:** Jaewon Jung, Hyunjae Kim.

**Visualization:** Jaewon Jung, Hyunjae Kim.

**Writing – original draft:** Jaewon Jung, Hyunjae Kim.

**Writing – review & editing:** Jaewon Jung, Hyunjae Kim.

## Supplementary Material


