# Supplementary material for: Comparative analysis of multimodal large language models GPT-4o and o1 versus clinicians in clinical case challenge questions: Retrospective cross-sectional study
Source: Medicine (Baltimore). 2026 Jan 23;105(4):e47071. doi: 10.1097/MD.0000000000047071 (PMC12851745; doi:10.1097/MD.0000000000047071)
Supplement: Supplementary file 1 [file medi-105-e47071-s001.pdf]

Supplementary Figure S1. Graphical abstract of study design and main results

Comparative Analysis of Multimodal Large Language Models GPT-4o and o1 Versus Clinicians in Clinical Case Challenge Questions

Subjects

Medscape case challenge from May 2011 to June 2024 (N=1426)

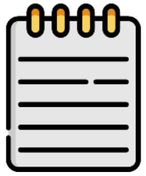

Text
Patient history, Physical Examinations, Diagnostic tests

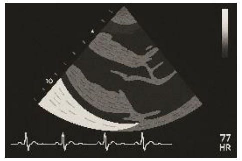

Images
MRI, CT, X-ray, Ultrasound, Pathological Images

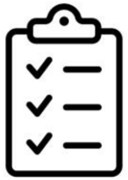

Question & Options
Diagnosis
Disease characteristics
Examinations, and Treatment

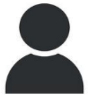

Medscape respondents

vs.

GPT models
Multimodal LLM processed with predefined prompt

Results

Model Accuracy

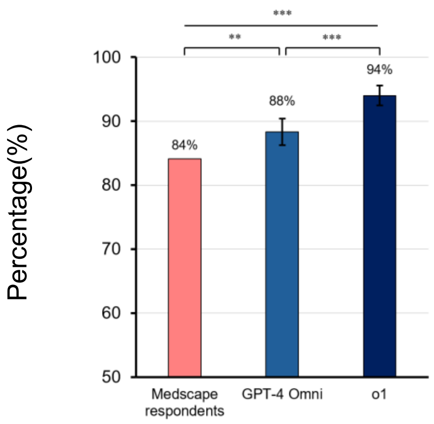

Response Consistency

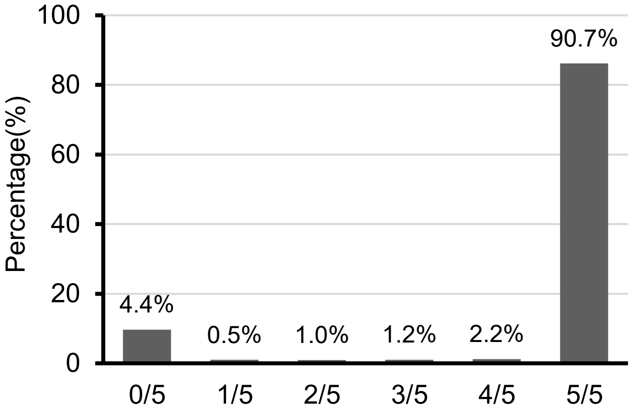

o1 showed high accuracy and consistency on image-based clinical cases

Performance by Question Category

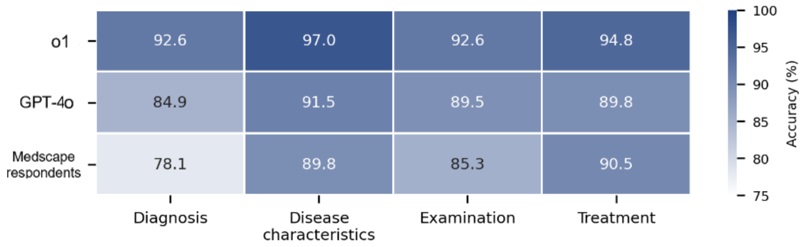

GPT models showed superior performance to Medscape respondents, especially in diagnosis-related cases

Conclusion

GPT-4o and o1 outperformed clinicians in solving structured scenarios, including image-based scenarios. These LLMs demonstrated strong diagnostic capability for medical decision support.

Figure S2. Medscape quiz sample

A 19-Year-Old Woman With Fever, Cough, and Dyspnea at Rest

Background, Physical Examination and Workup

A 19-year-old White woman with no relevant past medical problems presents to the emergency department (ED) with high-grade fever with chills, cough, and dyspnea at rest. Her symptoms have been ongoing for 1 week. She works at a medical office as a receptionist and initially thought she contracted something from one of the patients. However, her symptoms were not getting better, so she decided to come to the ED for evaluation. She denies any recent travel to high-risk countries, has no pets at home, nor has had exposure to smoke or chemicals. Patient takes no medications other than birth control pills and has been otherwise healthy.

Her parents both have well-controlled diabetes and no other medical issues. Her older sister has asthma, for which she uses an albuterol inhaler. She has no prior hospitalizations, and she has been up to date on all her vaccinations.

Prior to coming to the ED, she sought help at an urgent care for her symptoms and she was prescribed a azithromycin for 5 days that she has finished but had no improvement of symptoms. No imaging was performed at the urgent care. Her fever ranges from 100 °F to 101 °F, and her cough is not productive. She has used ibuprofen and acetaminophen to control her fever. She has felt slightly short of breath since her illness began, but this morning, her shortness of breath was the most distressing symptom and she decided to come to the ED for further evaluation.

On arrival in the ED the patient's heart rate was 120 beats/min, respiratory rate was 26 beats/min, body temperature was 102.9 °F, and her oxygen saturation was 90% on room air. On physical exam, there were diminished breath sounds bilaterally, but it was otherwise unremarkable. She was started on supplemental oxygen at 6L/min, 650 mg of acetaminophen, and 1L of normal saline fluid bolus.

A chest radiograph revealed bibasilar pneumonia. Due to concern for pulmonary embolism (PE), a CT of the chest with contrast was done that showed bibasilar consolidation and interlobular septal thickening with diffuse ground glass opacities especially in the bases (Figure 1). Per protocol, she was started on respiratory isolation and a swab for SARS-CoV-2 was sent.

Figure 1. Bilateral interlobular septal thickening with mild diffuse ground glass opacities and subtle ill-defined nodular densities.

The patient was admitted to the hospital and kept under respiratory isolation until all infectious etiologies were ruled out. She was empirically started on antibiotics (2 g of IV ceftriaxone and 500 mg IV azithromycin) for presumptive bacterial pneumonia given the imaging findings. The patient was continued on IV fluids and 4 L/min of oxygen via nasal cannula to keep her saturation above 92%.

Pertinent laboratory values on admission were:

- White blood cell count: 13,800 /µL (reference range, 4500-11,000/µL)
- Low-density lipoprotein cholesterol: 335 ng/mL (reference range, 140-280 ng/mL)
- C-reactive protein: 221 mg/L (reference range, < 3 mg/L)
- Ferritin- 311 (reference range, 13-150 ng/mL)
- D-dimer: 1449 ng/ml (reference range, 220-500 ng/mL)
- Procalcitonin 0.1 ng/mL (reference range, 0.05 ng/mL)
- SARS-CoV-2 antigen nucleic acid amplification test: negative
- Influenza A/B: negative
- Viral pathogen panel: negative

The patient was continued on IV antibiotics, fluids, and supplemental oxygen for 48 hours but did not show any signs of Improvement. A pulmonologist was consulted due to atypical nature of her symptoms and lack of clear diagnosis since patient had no improvement. When questioned by the pulmonologist about tobacco use, the patient reported vaping e-cigarettes over the last month. Patient reports daily use of e-cigarettes given to her by her boyfriend.

Images

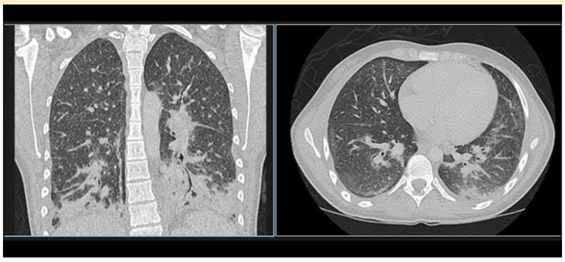

Question

Which is the best next step in the workup and management of the patient?

Options

① Broaden antibiotics to vancomycin and piperacillin tazobactam and get sputum for bacteria

② Refer the patient for bronchoscopy to get broncho-alveolar lavage cultures

③ Diagnose the patient with e-vaping induced lung injury and start the patient on prednisone

④ Order mycoplasma antigen and continue empirically on azithromycin

A 30-Year-Old Man With Paranoia and Vomiting After a Part

Background, Physical Examination and Workup

A 30-year-old man who was previously healthy is brought by his friend to the emergency department owing to visual hallucinations, vomiting, and paranoia. The patient had arrived in town earlier that day and reports mild diffuse abdominal pain and lightheadedness when he stands up. He denies any vertiginous symptoms. His friend reports that he has had these symptoms for the past 2 hours. The patient complains of seeing "people moving in the room" but denies any auditory hallucinations.

The patient denies chest pain, shortness of breath, diarrhea, blood in the stool or vomit, and recent fever. In recounting the events leading up to the start of the patient's symptoms, his friend describes they had been to a house party, as well as several bars and breweries throughout the afternoon.

The patient has no other medical problems and is not currently under the care of a physician. No medication use is reported by the patient, his family, or his friends. Upon questioning, the patient states that he smokes tobacco "occasionally," has tried cannabis "a few times," and drinks four or five alcoholic beverages per week.

The patient appears anxious. He is vomiting and tearful. His heart rate is tachycardic, at over 130 beats/min. His blood pressure is 140/89 mm Hg, respiration rate is 20 breaths/min, and temperature is 98.6°F (37°C). His pupils are dilated and reactive; mild conjunctival injection is noted. His skin is diaphoretic and warm to the touch.

Results of the cardiac examination are normal, with the exception of tachycardia. His lungs are clear to auscultation bilaterally. Examination of the abdomen reveals mild diffuse tenderness without focal peritonitis or localized discomfort. The neurologic examination demonstrates normal cranial nerve testing results, normal strength and sensation in the upper and lower extremities, normal reflexes, and normal gait testing results. However, the patient is experiencing lightheadedness when he stands up. The patient is alert and oriented to self and place but remains agitated and paranoid. No clonus or muscle rigidity is noted. Deep tendon reflexes are normal. The patient is able to provide a urine sample.

The initial diagnostic workup in the ED includes ECG, complete blood cell count, basic metabolic panel, urine drug screen, and ethanol level. Owing to the patient's undifferentiated altered mental status and vomiting, noncontrast head CT is ordered as well.

The ECG shows sinus tachycardia with a normal axis, normal intervals, no ST elevations, no ST depressions or T wave inversions, no Brugada sign, no delta wave, and no prolonged QT.

An example of an ECG similar to the patient's is shown below

Other findings are as follows:

- White blood cell count: 11 × 10^9 cells/L (reference range, 4.5-11.0 × 10^9 cells/L)
- Hemoglobin level: 14 g/dL (reference range, 13.5-17.5 g/dL)
- Platelet count: 350 × 10^9 cells/L (reference range, 150-400 × 10^9 cells/L)
- Sodium level: 129 mEq/L (reference range, 135-145 mEq/L)
- Potassium level: 3.2 mEq/L (reference range, 3.7-5.2 mEq/L)
- Chloride level: 90 mEq/L (reference range, 98-106 mEq/L)
- Bicarbonate level: 26 mEq/L (reference range, 23-30 mEq/L)
- Blood urea nitrogen level: 10 mg/dL (reference range, 7-20 mg/dL)
- Creatinine level: 1 mg/dL (reference range, 0.7-1.2 mg/dL)
- Glucose level: 150 mg/dL (reference range, 74-106 mg/dL)
- Creatine kinase level: 200 IU/L (reference range, 55-170 IU/L)
- Troponin level: < 0.01 ng/mL (reference range, 0-0.4 ng/mL)

The results of the urine drug screen and the ethanol level are pending. Noncontrast head CT reveals no acute intracranial abnormalities. An example of a CT scan similar to the patient's is shown below (Figure 2).

Images

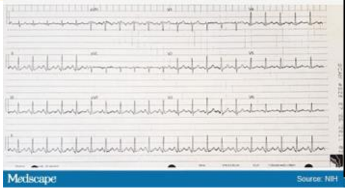

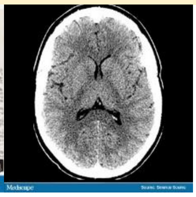

Question

On the basis of these findings only, which of the following is the most likely diagnosis?

Options

① Infectious encephalitis

② Serotonin syndrome

③ Cannabinoid toxicity

④ Anticholinergic toxicity

Table S1. Medscape quiz information

No.	Medical specialty	Case summary	Question	Answer
Case 1	Internal Medicine	A 13-year-old boy presents with fever, headache, nausea, lethargy, incoherence, and nuchal rigidity, with a Glasgow Coma Scale score of 9-10, and no rashes or trauma observed. Laboratory results show a WBC count of 16,000/µL with a left shift, elevated inflammatory biomarkers, and lumbar puncture findings of high opening pressure, elevated WBC and RBC counts, high protein, and low glucose, while imaging studies are normal and Gram staining is negative.	Diagnosis	Naegleria fowleri meningoencephalitis
			Disease characteristics	Nasal inhalation of N fowleri-contaminated fresh water
			Treatment	5 days
Case 2	Internal Medicine	A 17-year-old girl presents with chronic oral thrush, diarrhea, abdominal pain, vomiting, dizziness, skin hyperpigmentation, and amenorrhea, with physical examination revealing pallor, dehydration, and hypotension. Laboratory results show hypochromic microcytic anemia, low serum cortisol, elevated ACTH, high potassium, and normal imaging studies, with Candida albicans identified in oral swabs.	Diagnosis	Polyglandular failure (type I)
			Diagnosis	PGA-I requires lifelong treatment of the manifestations of the disorders
			Treatment	For the diagnosis of PGA-I, the patient should have at least 2 components of the typical triad of AD, hypoparathyroidism, and evidence of mucocutaneous candidiasis
Case 3	Internal Medicine	A 19-year-old man presents with progressive confusion, headache, drowsiness, hypertension, pericardial rub, and 1+ pitting edema in the lower extremities, with crackling and decreased breath sounds at the lung bases. Laboratory results show elevated potassium, low bicarbonate, high blood urea nitrogen, elevated creatinine, anemia, thrombocytopenia, and urinalysis with RBCs, RBC casts, and proteinuria, while imaging reveals small bilateral pleural effusions, a small pericardial effusion, and normal brain CT and kidney appearance.	Diagnosis	Lupus nephritis
			Examination	Immunostaining for IgA, IgG, IgM, C3, and C1q
			Disease characteristics	Anti-dsDNA antibody titer
Case 4	Minor	A 19-year-old woman with obesity presents with diffuse headaches, transient visual obscurations, pulsatile tinnitus, reduced visual acuity to 6/12, left sixth nerve palsy, and bilateral optic disc swelling. Laboratory tests are normal, lumbar puncture shows elevated opening pressure, MRI reveals intraocular protrusion of the left optic nerve head, and Goldmann perimetry indicates an enlarged blind spot.	Diagnosis	Pseudotumor cerebri
			Examination	Blindness
			Disease characteristics	Intraocular protrusion of optic nerve head
Case 5	Internal Medicine	A 25-year-old woman presents with lightheadedness, dyspnea, vague joint aches, and fatigue, with physical examination revealing bradycardia but no respiratory distress or other abnormalities. Laboratory tests, including a complete blood cell count, basic metabolic panel, cardiac troponin, and pro B-type natriuretic peptide, are unremarkable, while imaging shows a normal cardiac silhouette and no pericardial effusion or wall motion abnormality.	Diagnosis	Lyme carditis
			Treatment	History of erythema migrans and tick exposure with conduction delay (laboratory tests still pending)
			Treatment	Ceftriaxone
Case 6	Internal Medicine	A 28-year-old man presents with 8 hours of nausea, vomiting, and diarrhea, accompanied by dry mucus membranes, low-grade tachycardia, hyperactive bowel sounds, and mild diffuse abdominal tenderness. Laboratory results from a basic chemistry panel are pending, and an intravenous line is started for fluid administration.	Diagnosis	Gastroenteritis
			Diagnosis	Norovirus
			Treatment	Hydration
Case 7	Major Surgery	A 28-year-old woman presents with 14 hours of nonbloody, bilious vomiting, constant lower abdominal cramping, and new onset watery diarrhea, accompanied by lethargy, fever, tachycardia, and diffuse abdominal tenderness without rebound or guarding. Laboratory results show leukocytosis with neutrophilia, hypoalbuminemia, hyponatremia, and hyperchloremia, while imaging includes an unremarkable chest radiograph and a transvaginal ultrasound.	Diagnosis	Ovarian hyperstimulation syndrome
			Disease characteristics	A 24-year-old egg donor who had a prior episode of ovarian hyperstimulation syndrome with previous donation and who had 24 follicles and an estradiol level of 3624 pg/mL before

				human chorionic gonadotropin administration
			Disease characteristics	Diuretics should be used during the treatment of ovarian hyperstimulation syndrome
Case 8	Minor	A 29-year-old woman presents with intensely pruritic, erythematous, oozing plaques with vesicles and excoriations on her feet, worsened by hiking and self-treatment with coconut oil. Physical examination shows inflamed plaques on both feet, with intact dull and sharp sensations, and vital signs are within normal limits except for a slight fever.	Diagnosis	Allergic contact dermatitis
			Examination	Suggest that the patient wear shoes with a solid upper portion
			Treatment	Biopsy of a positive patch test for allergic contact dermatitis would reveal thick lymphocytic infiltrates in the upper dermis
Case 9	Internal Medicine	A 35-year-old man presents with confusion, headache, fever, chills, and diffuse anterior chest wall pain, with physical examination showing an ill appearance but no acute distress, normal neurologic and cardiac exams, and clear lungs. Laboratory results reveal leukocytosis with eosinophilia, elevated troponin, and abnormal ECG with ST-segment depression, while imaging shows no acute intracranial abnormality, suspected splenomegaly, normal coronary anatomy, and cardiac MRI indicating subendocardial and mid-myocardial delayed enhancement with a laminated LV apical thrombus, leading to suspicion of acute leukemia with eosinophilia.	Diagnosis	Eosinophilic myocarditis
			Examination	ACE inhibitors
			Treatment	Endomyocardial biopsy
Case 10	Paediatrics	A 5-month-old boy presents with severe abdominal distention, lethargy, pallor, flaccidity, and unresponsiveness, with physical examination revealing a depressed anterior fontanel, dry mucous membranes, weak pulses, tachycardia, hypotension, and a tense, discolored abdomen. Laboratory and imaging results show severe metabolic acidosis with elevated lactate, hemoglobin of 6 g/dL, leukocytosis, neutrophilia, thrombocytopenia, hyperkalemia, and a large, septated cystic mass under the liver with bloodstained free fluid on paracentesis.	Diagnosis	Ruptured hepatic tumor
			Disease characteristics	Ultrasonography
			Examination	Mesenchymal hamartomas of the liver require histologic confirmation for definitive diagnosis
Case 11	Minor	A 61-year-old woman presents with sleep disruption, irritability, headaches described as sharp and electric-shock-like, weight loss, and extreme sensitivity on the left side of her face, with physical examination revealing high blood pressure and a stress-related rash on her left forearm. Laboratory results are normal, and brain MRI shows a small hyperintensity in the left internal capsule, consistent with a prior infarct, with no other abnormalities.	Diagnosis	Trigeminal neuralgia
			Diagnosis	Carbamazepine or oxcarbazepine
			Treatment	Clinical history and physical examination findings
Case 12	Paediatrics	A 7-month-old boy presents with lethargy, abdominal pain, inconsolable crying, vomiting, and passing mucous bloody stool, with physical examination showing high-pitched peristalsis and rigid abdominal muscles during pain episodes. Laboratory results reveal mild leukocytosis with a left shift, normal electrolytes, and imaging includes plain abdominal radiography and ultrasound.	Diagnosis	Intussusception
			Examination	Surgical exploration
			Examination	Air enema
Case 13	Minor	A 5-year-old boy presents with a "gleam" in his left eye, mild esotropia, conjunctival congestion, anisocoric pupils, an afferent pupillary defect, and absent red reflex in the left eye, with tenderness preventing a complete examination. Imaging and laboratory findings reveal a yellowish mass, total retinal detachment, neovascularization of the iris, closed anterior chamber angle, elevated intraocular pressure of 50 mm Hg in the left eye, and total serous retinal detachment on B-scan ultrasonography.	Diagnosis	Coats disease (exudative retinitis)
			Examination	Leukocoria
			Disease characteristics	Biopsy
Case 14	Internal Medicine	A 10-year-old boy presents with multiple fainting spells and generalized tonic-clonic seizure-like activity, often occurring in the early morning or during sports, with a normal physical examination. Laboratory analysis,	Diagnosis	Congenital long QT syndrome
			Disease characteristics	Beta-blockers

		chest radiography, and brain CT are normal, while the ECG shows abnormalities.	Treatment	Congenital optic atrophy
Case 15	Psychiatry	A 12-year-old girl presents with problems at school, including social withdrawal, decreased participation, and a slight drop in grades, and during examination, she exhibits soft quiet speech, occasionally puts her head down, and appears tearful. Laboratory tests and ECG are normal, while the Patient Health Questionnaire adolescent version reveals a score of 14, indicating clinical depression with symptoms of low energy, sadness, irritability, lack of appetite, disinterest in activities, and thoughts of death without a specific plan.	Diagnosis	Major depressive disorder
			Treatment	Fluoxetine
			Treatment	Cognitive-behavioral therapy
Case 16	Psychiatry	A 12-year-old boy presents with falling grades, forgetful behavior, anxiety, and disruptive behavior at school, with a physical examination showing intact memory, coherent thoughts, and no hallucinations or delusions, but he becomes fidgety and blotchy red when performing tasks requiring extended attention. Laboratory and imaging results are normal, with no hearing or sight problems, and a psychoeducational evaluation indicates difficulty focusing, abandoning tasks, and frequent daydreaming.	Diagnosis	Attention deficit hyperactivity disorder
			Disease characteristics	Norepinephrine and dopamine
			Disease characteristics	Often picks at one’s skin
Case 17	Minor	A 12-year-old boy presents with bilateral arm pain, weakness, numbness, inability to grasp objects, urinary retention, and decreased upper-extremity strength, with physical examination showing tenderness from T2-T10, inability to abduct arms, and a postvoid residual bladder volume of 1 L. Laboratory tests reveal normal complete blood cell count and basic metabolic panel, while cervical and thoracic spine MRI is ordered.	Diagnosis	Central cord syndrome
			Disease characteristics	New-onset urinary retention in a patient with 4 days of back pain
			Disease characteristics	Motor paresis that is greater in the upper extremities than in the lower extremities
Case 18	Minor	A 13-year-old athletic boy presents with substernal chest pain extending into the neck, aggravated by deep breaths, and palpable crepitus around the neck base, with normal vital signs and no respiratory distress. Laboratory results are normal, and imaging reveals extensive pneumomediastinum with subcutaneous emphysema, pneumopericardium, and air in the pleural space.	Diagnosis	Pneumorrhachis
			Disease characteristics	1-2 days
			Disease characteristics	Spinal cord compression
Case 19	Paediatrics	A 13-year-old boy presents with recurrent mild pain in the left lower leg, severe localized tenderness, soft-tissue swelling, and mild skin reddening over the lower left tibial prominence. Laboratory results are within normal limits, while imaging reveals a well-demarcated, lytic, lobulated lesion in the tibia with a subtle fracture, and histopathology suggests a benign cartilaginous tumor.	Diagnosis	Chondromyxoid fibroma
			Examination	Recurrence has been described in < 3% cases
			Disease characteristics	Histopathologic analysis
Case 20	Internal Medicine	A 13-year-old girl presents with worsening shortness of breath, malaise, fever, loose stools, tachycardia, tachypnea, and headache, with physical examination showing tenderness in the bilateral temporal areas and mild generalized abdominal tenderness. Laboratory findings reveal leukocytosis, mild anemia, elevated inflammatory markers, and a brain MRI limited by artifact but showing no acute intracranial abnormality, with negative cerebrospinal fluid studies.	Diagnosis	Hyperthyroidism
			Disease characteristics	Beta-blockers
			Treatment	Constipation
Case 21	Internal Medicine	A 14-year-old boy presents with a 2-week history of fever, fatigue, mild headaches, neck stiffness, dry fissured lips, skin peeling, conjunctival injection, lymphadenopathy, and a desquamating rash. Laboratory results show elevated white blood cell and platelet counts, slightly low hemoglobin, and a coronary CT angiography is performed for cardiac evaluation.	Diagnosis	Coronary artery aneurysm
			Disease characteristics	Hepatosplenomegaly
			Disease characteristics	All of the above
Case 22	Internal Medicine	A 15-year-old girl presents with steatorrhea, poor night vision, pale conjunctiva, macrocytic anemia, and a mildly distended abdomen with hyperactive bowel sounds, along with ecchymosis on the right thigh. Laboratory results show macrocytic anemia, prolonged prothrombin time, and mild hypoalbuminemia, while other tests are normal.	Diagnosis	Exocrine pancreatic insufficiency (EPI)
			Examination	Lifestyle modifications
			Treatment	PFTs
Case 23	Paediatrics		Diagnosis	Osteochondral defect

		A 16-year-old boy presents with acute-onset left knee pain, swelling, and positive patellar ballottement, with limited range of motion due to pain. Laboratory results show a normal complete blood count, and knee x-rays along with arthrocentesis reveal 30 mL of old blood without evidence of infection.	Disease characteristics	If MRI is unavailable, CT is the preferred method
			Examination	OCD mainly occurs in the knee
Case 24	Minor	A 16-year-old girl presents with generalized pruritus, eye swelling, full-body rash, and shortness of breath, accompanied by periorbital angioedema, facial erythema, and bilateral diffuse wheezes on physical examination. Laboratory and imaging results are not provided, but treatment with epinephrine, diphenhydramine, methylprednisolone, and nebulized albuterol resolved her symptoms within 30 minutes.	Diagnosis	Exercise-induced anaphylaxis
			Treatment	Epinephrine
			Disease characteristics	Wheat
Case 25	Major Surgery	A 16-year-old girl presents with primary amenorrhea and delayed breast development, with a Sexual Maturity Rating of II for breast development and III for pubic hair growth, and no significant acne, hirsutism, or other abnormal physical findings. Laboratory results show elevated luteinizing hormone and follicle-stimulating hormone levels, low anti-Müllerian hormone and inhibin A levels, and a karyotype of 46,XY with a MAP3K1 mutation, while pelvic ultrasonography reveals a small uterus and streak ovaries.	Diagnosis	Swyer syndrome
			Disease characteristics	X-linked recessive
			Disease characteristics	Turner syndrome
Case 26	Paediatrics	A 19-year-old man presents with morbid obesity, oozing skin ulcers, delayed motor milestones, short stature, and absence of facial and axillary hair, with physical examination revealing generalized obesity, small hands and feet, and erythematous, dry, scaly skin. Laboratory and imaging results show normal liver function, mild cerebral atrophy on MRI, normal high-resolution CT scans, delayed bone age, nonspecific dermatitis on skin biopsy, and hormonal studies indicating low serum cortisol and ACTH levels.	Diagnosis	Prader-Willi syndrome
			Disease characteristics	Ghrelin
			Disease characteristics	Gene deletion
Case 27	Internal Medicine	A 19-year-old man with a history of ileocolonic Crohn's disease presents asymptomatically with a slightly enlarged liver and normal physical examination findings. Laboratory tests reveal dyslipidemia, elevated liver enzymes, and elevated inflammatory markers, while imaging shows chronic active colitis and an enlarged liver with normal bowel signals, and serology for various conditions is negative.	Diagnosis	Primary sclerosing cholangitis
			Disease characteristics	Small-duct disease
			Disease characteristics	Periductal fibrosis
Case 28	Internal Medicine	A 19-year-old woman presents with high-grade fever, chills, cough, dyspnea at rest, tachycardia, tachypnea, and diminished breath sounds bilaterally. Laboratory results show elevated white blood cell count, C-reactive protein, ferritin, and D-dimer, with negative SARS-CoV-2 and influenza tests, while imaging reveals bibasilar consolidation, interlobular septal thickening, and diffuse ground glass opacities.	Treatment	Diagnose the patient with e-vaping-induced lung injury and start the patient on prednisone
			Disease characteristics	Both A and B
			Diagnosis	Both A and B
Case 29	Paediatrics	A 20-year-old man presents with mutism, constant grimacing, slow movements, mild right-sided weakness, and a stooped posture, with a history of attentional and learning disabilities, impaired motor coordination, and unusual behaviors. Laboratory and imaging results show normal lumbar puncture and MRI, nonreactive serum serology, negative antinuclear and HIV antibody titers, normal serum ceruloplasmin, absence of metachromatic leukodystrophy enzymatic defect, normal mitochondrial enzymes, loss of axon cylinders in sural nerve biopsy, perimysial fibrosis and type II fiber predominance in deltoid biopsy, increased theta and delta waves on QEEG, and decreased [18F]FDG PET uptake in the	Diagnosis	Autism with catatonia
			Disease characteristics	Autonomic instability
			Disease characteristics	Good eye-hand coordination
Case 30	Minor	A 20-year-old woman presents with bilateral flank pain, episodic vomiting, malar angiofibromas, a leathery patch on her back, periungual fibromas, and palpable cystic-to-firm masses in the flanks. Laboratory results show anemia and urine examination reveals albuminuria and microscopic hematuria, while imaging	Diagnosis	Tuberous sclerosis
			Examination	Angiomyolipoma
			Disease characteristics	Pituitary mass

		indicates bilateral renal angiomyolipomas and subependymal calcified nodules in the brain.		
Case 31	Internal Medicine	A 21-year-old man presents with 10-hour epigastric pain radiating to the chest, with a slightly tender but soft epigastrium on examination. Laboratory results are normal, and imaging reveals a radio-opaque shadow in the central lower chest/epigastrium region, prompting endoscopic intervention.	Diagnosis	Foreign-body ingestion
			Examination	Upper esophagus
			Disease characteristics	Lateral x-ray
Case 32	Psychiatry	A 21-year-old woman presents with a 3-month history of abdominal discomfort, loss of appetite, diminished academic performance, and anxiety about group work, with physical examination showing normal vital signs and no abdominal tenderness. Laboratory tests, including a complete blood count and electrolytes, are normal, and an abdominal CT scan reveals no structural abnormalities.	Diagnosis	Anxiety
			Disease characteristics	Counseling and possibly medication
			Treatment	Disruption of thalamic inhibitory control
Case 33	Internal Medicine	A 21-year-old woman presents with a 1-month history of fever, malaise, abdominal pain, productive cough with blood-tinged sputum, night sweats, weight loss, and shortness of breath, with physical examination revealing tachycardia, fever, tachypnea, cachexia, and crackles in the right lung field. Laboratory results show anemia, low CD4 count, high HIV viral load, elevated liver enzymes, and elevated LDH, while imaging includes chest radiography and CT of the chest.	Diagnosis	Histoplasmosis
			Treatment	Itraconazole
			Examination	CSF Histoplasma antigen
Case 34	Minor	A 22-year-old man presents with muscle pain, weakness, confusion, and tender, doughy muscles after collapsing during football practice in hot, humid conditions, with vital signs showing mild fever and tachycardia. Laboratory results reveal elevated creatinine and creatin phosphokinase levels, positive urinalysis for blood without red cells, and imaging shows normal CT and MRI of the brain, with ECG indicating sinus tachycardia.	Diagnosis	Heat stroke or exercise-induced rhabdomyolysis
			Treatment	Vigorous hydration and electrolyte replacement
			Treatment	Start IV fluids immediately
Case 35	Internal Medicine	A 22-year-old man presents with loss of consciousness while climbing stairs, a parasternal heave, a pansystolic murmur at the left lower sternal edge, a thrill in the suprasternal region, and severe clubbing of the fingers. Laboratory and imaging results show sinus rhythm with right axis deviation, right ventricular hypertrophy on ECG and chest radiography, a hypokinetic small-volume left ventricle with an ejection fraction of 40%, a hypertrophied septal wall, a ventricular septal defect with a right-to-left shunt, an enlarged right ventricle and atrium, subvalvular right ventricular outflow tract obstruction, a hematocrit level of 56.8%, and lactate	Diagnosis	Tetralogy of Fallot
			Examination	To help force more blood through the pulmonary valve
			Disease characteristics	MRI
Case 36	Internal Medicine	A 23-year-old woman presents with generalized muscle weakness, nonbilious emesis, and intermittent hematuria, with physical examination revealing 2/5 strength in extremities and diminished reflexes. Laboratory results show a positive urine pregnancy test, low bicarbonate with metabolic acidosis (pH 7.25), and urine analysis with moderate blood, while ECG and renal ultrasound are performed.	Diagnosis	Distal renal tubular acidosis
			Examination	Challenge with ammonium chloride, which should cause the urine pH to remain > 5.5 in type 1 RTA
			Disease characteristics	Hyperkalemia
Case 37	Psychiatry	A 23-year-old woman presents with left eye blindness, left lower extremity weakness, and ecchymosis on the right forehead after a fall, with a calm but detached demeanor and intact cranial nerves. Laboratory tests, urine toxicology, and a head CT scan are normal, with no evidence of cerebrovascular accident or mass lesions.	Diagnosis	Conversion disorder
			Diagnosis	Prior or recent psychological trauma
			Disease characteristics	Esophageal spasms
Case 38	Paediatrics	A 24-year-old man with Down syndrome presents with bilateral flank pain, hematuria, and shortness of breath, with physical examination showing mild tachycardia and oxygen saturation of 92% on room air. Laboratory results reveal elevated alkaline phosphatase, low albumin, elevated lactate dehydrogenase, and a slightly elevated international normalized ratio, while imaging includes a chest radiograph.	Diagnosis	Metastatic testicular cancer
			Examination	Ultrasonography and serum testing for tumor markers
			Treatment	Orchiectomy, chemotherapy, and radiation therapy
Case 39	Minor		Diagnosis	Ramsay Hunt syndrome

		A 24-year-old man with HIV presents with left ear pain, dizziness described as vertigo, muffled hearing, erythematous and edematous left ear with vesicles, left-beating nystagmus, and subtle left facial droop with weakness. Laboratory and imaging results are not provided in the case details.	Disease characteristics	Involvement of the cornea
			Treatment	Amoxicillin
Case 40	Minor	A 25-year-old pregnant woman presents with worsening unilateral, pulsatile headaches accompanied by nausea, photophobia, and phonophobia, with normal physical and neurological examinations. MRI prior to pregnancy shows small subcortical white matter T2 hyperintensities.	Diagnosis	Migraine with aura
			Treatment	Propranolol
			Treatment	Acetaminophen
Case 41	Minor	A 25-year-old woman presents with left eye pain, double vision, mild ptosis, and limited eye adduction, with a history of similar episodes and a recent upper respiratory infection. Laboratory tests are unremarkable, and MRI with magnetic resonance angiography is performed.	Diagnosis	Infiltrative lesion of the left oculomotor nerve
			Disease characteristics	Cisternal segment at the root exit zone
			Diagnosis	Exclusion of orbital, parasellar, or posterior fossa lesion using appropriate neuroimaging
Case 42	Internal Medicine	A 26-year-old man involved in a high-speed motor vehicle collision presents with combative behavior, hypotension, tachycardia, tachypnea, hypoxia, tracheal deviation to the right, decreased breath sounds, and hyperresonance on the left chest, with elevated jugular venous pressure. Chest radiograph confirms the clinical suspicion of tension pneumothorax, leading to significant improvement after an emergent procedure.	Diagnosis	Tension pneumothorax; needle thoracostomy
			Treatment	Immediate drainage of > 2000 mL of bloody fluid
			Disease characteristics	Increased sympathetic tone
Case 43	Internal Medicine	A 26-year-old man presents with acute altered mental status, obtundation, large reactive pupils, dry mucous membranes, and a Glasgow Coma Scale score of 7, with no response to naloxone and normal vital signs. Laboratory studies are pending, a chest radiograph shows no acute disease, a head CT is normal, and an ECG reveals an abnormal rhythm with a prolonged QTc interval of 472 ms.	Treatment	Initiate therapy with sodium bicarbonate
			Disease characteristics	Hemodialysis
			Treatment	Diaphoresis
Case 44	Internal Medicine	A 26-year-old man presents with recurrent colicky abdominal pain, intermittent distention, vomiting, and visible peristalsis, with physical examination showing a distended but soft abdomen and hyperactive bowel sounds. Laboratory results reveal mild anemia, elevated blood urea nitrogen, and hyponatremia, while imaging shows multiple air-fluid levels and distended small-bowel loops with hyperperistalsis, indicating a small-bowel obstruction.	Diagnosis	Small-bowel obstruction due to a giant ileal diverticulum
			Disease characteristics	If the patient has evidence of steatorrhea or pernicious anemia, he should initially receive a course of antibiotics and vitamins
			Treatment	Small bowel diverticula are commonly situated in the antimesenteric border of the intestine
Case 45	Internal Medicine	A 26-year-old woman presents with severe knee, hip, and shoulder pain, requiring a cane for walking, decreased range of motion, and tenderness in the left tibia, along with abnormal dentition and a nonhealing avulsion fracture in the right first metatarsal. Laboratory results show normal calcium, phosphorus, albumin, vitamin D, and vitamin B6 levels, with low alkaline phosphatase, while imaging confirms the avulsion fracture.	Diagnosis	Hypophosphatasia
			Disease characteristics	Inorganic pyrophosphate
			Disease characteristics	ALPL
Case 46	Internal Medicine	A 26-year-old woman presents with nausea, vomiting, diarrhea, lower abdominal pain, dysuria, fever, generalized pallor, abdominal distention with tenderness, ascites, and bilateral pleural effusion. Laboratory and imaging results show normocytic anemia, elevated ESR, urinalysis with RBCs, WBCs, and protein, bilateral hydronephrosis, thickened mesentery and gut wall, peritoneal deposits, exudative ascites with lymphocytes, elevated CA-125, and CT confirming ascites, peritoneal nodularity, and enlarged lymph nodes.	Diagnosis	Systemic lupus erythematosus
			Diagnosis	Smooth muscle hypomotility
			Diagnosis	Vasculitis
Case 47	Minor	A 27-year-old man presents with balance problems, leg stiffness, and several episodes of bladder incontinence, with physical examination revealing brisk lower	Diagnosis	Adrenomyeloneuropathy
			Disease characteristics	The condition could predispose to an injury, and

		extremity reflexes, upgoing toes, and spasticity, particularly in the lower extremities. Laboratory tests show normal complete blood cell count and electrolytes, while MRI of the lumbar spine reveals demyelination of the lateral dorsal columns, and elevated plasma very long-chain fatty acid levels with low morning cortisol and elevated adrenocorticotropic hormone levels.		additional physical limitations may result from the injury
			Examination	Patients can be regularly assessed for development of early signs of motor changes
Case 48	Minor	A 27-year-old woman presents with white spots on her face, premature graying of hair, and sharply demarcated, depigmented macules above her left eyebrow, with normal vital signs and no other skin lesions. Laboratory tests show normal blood counts and thyroid hormone levels, while thyroid ultrasound reveals an enlarged gland with decreased echogenicity.	Diagnosis	Vitiligo
			Disease characteristics	Complete absence of melanocytes
			Treatment	Topical corticosteroids
Case 49	Internal Medicine	A 29-year-old man presents with multiple nonitchy skin-colored raised lesions, generalized weakness, dyspnea, and significant weight loss, appearing thin and malnourished on examination. Laboratory findings reveal elevated triglycerides at 1585 mg/dL, low HDL cholesterol at 14 mg/dL, and extremely high fasting and postprandial blood glucose levels of 550 mg/dL and 770 mg/dL, respectively, with skin biopsy showing hyperkeratosis and foamy histiocytes.	Diagnosis	Eruptive xanthoma
			Disease characteristics	Buttocks
			Disease characteristics	Foamy histiocytes containing lipid droplets
Case 50	Internal Medicine	A 29-year-old woman presents with multiple joint and muscle pain, back pain, fatigue, memory and concentration difficulties, and extreme tenderness to palpation over various body areas, with normal strength and reflexes but 4/5 strength in the upper extremities. Laboratory results show an unremarkable CBC, BMP, and UA, an ESR of 26 mm/h, a CRP of 0.5 mg/dL, normal thyroid function tests, a normal creatine kinase level, a low-positive ANA titer of 1:160, and a positive rheumatoid factor test.	Diagnosis	Fibromyalgia
			Treatment	Desipramine
			Treatment	Duloxetine
Case 51	Paediatrics	A 2-year-old girl presents with a 1-week history of worsening papular rash and intense pruritus on the trunk, arms, hands, and soles, with faint excoriations and withdrawal upon palpation. Laboratory and imaging results are not provided; however, the condition persisted despite treatment with a topical antibiotic and oral antihistamine, and the family history includes travel and potential exposure to similar lesions in the father.	Diagnosis	Scabies
			Disease characteristics	Infestation through close contact with infected animals
			Disease characteristics	Erythematous papules associated with burrows located on the child's cheeks
Case 52	Paediatrics	A 2-year-old boy presented with fever, malaise, generalized erythema with target-like lesions, central purpura, bullae, hemorrhagic lip and tongue ulcerations, and acute conjunctivitis with subconjunctival hemorrhage. Laboratory results showed mild anemia, low-grade leukocytosis, elevated IL-6 and CRP, normal electrolytes, and skin biopsy revealed full-thickness necrosis of the epidermis with mild dermal inflammatory cell infiltration.	Diagnosis	Stevens-Johnson syndrome
			Diagnosis	Diagnosis is primarily clinical, although some tests may help with confirmation
			Treatment	The patient should be hospitalized, with special attention to airway and hemodynamic stability, fluid status, wound/burn care, and pain control
Case 53	Psychiatry	A 30-year-old man presents with visual hallucinations, vomiting, paranoia, tachycardia, dilated pupils, and mild diffuse abdominal tenderness after attending a party. Laboratory results show hyponatremia, hypokalemia, and hyperglycemia, with a normal head CT and ECG showing sinus tachycardia.	Diagnosis	Cannabinoid toxicity
			Disease characteristics	Inhalation
			Disease characteristics	A 45-year-old previously healthy man with dark urine and a serum creatinine level > 4 mg/dL
Case 54	Minor	A 30-year-old woman presents with malaise, diffuse myalgia, a full-body purpuric rash, vomiting, confusion, headache, drowsiness, tachycardia, hypotension, and limited neck movement. Laboratory results show elevated white blood cell count, creatinine, blood urea nitrogen, myoglobin, creatine kinase, C-reactive protein, partial thromboplastin time, international normalized ratio, and D-dimer, with metabolic acidosis and high lactate levels on arterial blood gas analysis.	Diagnosis	Waterhouse-Friderichsen syndrome
			Disease characteristics	Waterhouse-Friderichsen syndrome is a complication of N meningitidis infection
			Treatment	A third-generation cephalosporin is required in areas where penicillin-resistant strains of Neisseria species have been identified

Case 55	Major Surgery	A 30-year-old pregnant woman at 23 weeks gestation presents with a history of complete placenta previa, no vaginal bleeding, and normal physical examination findings. Laboratory results are unremarkable, and imaging reveals complete placenta previa with focal myometrial thinning at the site of previous C-sections.	Diagnosis	Concurrent placenta previa and placenta accreta
			Diagnosis	Adenomyosis
			Disease characteristics	Placenta percreta
Case 56	Major Surgery	A 30-year-old woman presents with severe lower left abdominal pain, fever, tachycardia, cervical motion tenderness, and an 8-cm tender mass in the left adnexa. Laboratory results show an elevated white blood cell count, negative beta-human chorionic gonadotropin, and unremarkable basic chemistry and coagulation profiles, while ultrasound imaging reveals an adnexal mass.	Diagnosis	Tubo-ovarian abscess
			Disease characteristics	Tubo-ovarian abscess does not occur during an initial, acute episode of an STD
			Treatment	A trial of oral antibiotics
Case 57	Minor	A 31-year-old woman presents with intermittent right arm tremors, slightly decreased strength in the right biceps and triceps, diminished muscle tone in the right upper extremity, brisk reflexes, impaired coordination, and dysmetria on finger-to-nose testing with her right hand. Laboratory and imaging results are not provided in the case details.	Diagnosis	Demyelination
			Treatment	A course of intravenous steroids
			Treatment	Disease-modifying therapy
Case 58	Internal Medicine	A 32-year-old man presents with sudden hematemesis, nausea, black tarry stools, conjunctival pallor, tachycardia, hypotension, and enlarged cervical, axillary, and inguinal lymph nodes, with physical examination showing black guaiac-positive stool. Laboratory results reveal anemia, markedly elevated BUN and creatinine, high LDH, uric acid, and phosphorus levels, hematuria, leukocyte casts, uric acid crystals in urine, and imaging shows enlarged kidneys with hypoechogenic renal parenchyma, while endoscopy indicates erosive gastritis and biopsies show erythroid hyperplasia and reactive lymph nodes.	Diagnosis	Non-Hodgkin lymphoma
			Disease characteristics	Tumor lysis syndrome
			Disease characteristics	Age, serum LDH, performance status, Ann Arbor staging, and extranodal involvement
Case 59	Minor	A 33-year-old man presents with sudden severe vertigo, vomiting, loss of balance, left-sided facial numbness, mild left-sided ptosis, right-beating nystagmus, and left-sided dysmetria, with elevated blood pressure at 240/120 mm Hg. Laboratory tests are unremarkable, and noncontrast head CT images are provided for further evaluation.	Diagnosis	Lateral medullary syndrome
			Disease characteristics	Symptoms and signs include vertigo and ipsilateral ataxia
			Examination	The presence of a left occipital headache and mild neck pain should prompt investigation for arterial dissection
Case 60	Minor	A 33-year-old woman presents with a 2.5 cm × 2 cm immobile mass in the volar aspect of her right wrist, decreased muscle strength, decreased pinprick sensitivity sparing the fifth finger, and positive Tinel sign and Phalen maneuver. Laboratory and imaging results are not provided in the case details.	Diagnosis	Carpal tunnel syndrome
			Examination	Surgical decompression of the median nerve in the carpal tunnel by section of the flexor retinaculum is the standard approach
			Treatment	Ultrasonography
Case 61	Major Surgery	A 34-year-old pregnant woman presents with acute severe abdominal pain, light-headedness, loss of consciousness, hypotension, tachycardia, pallor, abdominal tenderness, and vaginal bleeding. Laboratory results show low hemoglobin and slightly elevated WBC count, while imaging reveals free fluid in the right upper quadrant and transvaginal ultrasonography findings in the left adnexa.	Diagnosis	Ruptured ectopic pregnancy
			Disease characteristics	None of the above
			Diagnosis	Fallopian tube
Case 62	Internal Medicine	A 34-year-old woman presents with painful, red "knots" on her right leg tracing along veins, generalized mottled skin discoloration, subungual red markings, oral ulcerations, and mild facial asymmetry, along with occasional fevers and joint pain. Laboratory findings show thrombocytopenia, hypochromic microcytic anemia, elevated serum creatinine, prolonged aPTT, and elevated ANA titers, with imaging confirming superficial thrombophlebitis in the right leg.	Diagnosis	Antiphospholipid syndrome
			Examination	Anticardiolipin and lupus anticoagulant
			Treatment	Warfarin
Case 63	Internal Medicine	A 34-year-old woman presents with vomiting, bulbar weakness, flaccid paraplegia, dysphonia, pendular nystagmus, and reduced palatal movements, with normal reflexes and intact sensations. Laboratory and imaging results show normal CBC, elevated CSF	Diagnosis	Sjögren syndrome
			Disease characteristics	Trigeminal nerve
			Disease characteristics	Rheumatoid arthritis

		protein with lymphocytic pleocytosis, aspiration pneumonia on chest radiograph, inconclusive nerve conduction studies, positive ANA, and MRI revealing high signal intensity in the medulla and abnormal signals in the thoracic spine.		
Case 64	Minor	A 35-year-old man presents with acute penile pain, swelling, and inability to urinate following trauma during intercourse, with physical examination revealing a swollen, ecchymosed penis and a full urinary bladder. Laboratory tests are normal, and MRI of the penis is performed, indicating the need for immediate surgical exploration.	Diagnosis	Penile fracture
			Examination	Urgent surgical exploration
			Treatment	MRI
Case 65	Minor	A 35-year-old man presents with moderate discomfort, multiple abrasions, contusions, and right shoulder tenderness with mild swelling and crepitus, but no open wounds or deformities. Laboratory tests are normal, and shoulder and chest radiographs are interpreted as normal, leading to a discharge diagnosis of shoulder contusion.	Diagnosis	Clavicle fracture
			Examination	False-positive error
			Examination	All of the above
Case 66	Internal Medicine	A 35-year-old man presents with dizziness and palpitations, accompanied by bradycardia and no joint swelling or neurological deficits on physical examination. Laboratory results are unremarkable, and electrocardiography shows normal sinus rhythm with a prolonged PR interval of 280 msec.	Diagnosis	Lyme disease
			Disease characteristics	Cardiac symptoms resolve with antibiotic treatment in most patients
			Disease characteristics	PR interval length
Case 67	Internal Medicine	A 35-year-old woman presents with amenorrhea, galactorrhea, dry skin, constipation, weight gain, and mild periorbital puffiness, with physical examination showing bilateral loss of the distal one third of the eyebrows and mildly prolonged relaxation phase of the Achilles tendon reflex. Laboratory results reveal elevated TSH, low free thyroxine, high anti-TPO antibody titer, elevated prolactin, and a pituitary MRI showing a 9-mm mass in the sella turcica without optic chiasm compression.	Diagnosis	Hashimoto thyroiditis
			Disease characteristics	Elevation of TRH by negative feedback on the hypothalamus
			Disease characteristics	Secondary hypothyroidism
Case 68	Psychiatry	A 35-year-old woman presents with a 10-month history of fatigue, muscle stiffness, joint pain, recurrent headaches, and difficulty concentrating, with physical examination showing pain on passive joint movements and muscle strength of 4/5 in extremities. Laboratory results reveal a low-positive ANA titer of 1:160, normal CBC, metabolic panel, urinalysis, ESR, CRP, thyroid function, and creatine kinase levels, with negative tests for HIV, Lyme disease, and viral hepatitis.	Diagnosis	Chronic fatigue syndrome
			Treatment	Major depression
			Disease characteristics	Prolonged rest
Case 69	Internal Medicine	A 36-year-old woman with irritable bowel syndrome presents with loose stools containing occasional bright red blood, intermittent abdominal pain relieved by defecation, and a history of internal hemorrhoids, with physical examination showing a soft, nontender abdomen and normal vital signs. Laboratory tests reveal normal inflammatory markers and negative stool tests for pathogens, but an elevated stool calprotectin level, while colonoscopy shows mild erythema, decreased vascular pattern, and mild friability throughout the colon with biopsies indicating diffuse active colitis and crypt abscesses.	Diagnosis	Ulcerative colitis
			Treatment	Oral 5-ASA 2 g/d
			Treatment	Ozanimod
Case 70	Internal Medicine	A 36-year-old woman presented minimally responsive with hypothermia, tachycardia, hypotension, poor hygiene, a Glasgow Coma Scale score of 8, tachycardia with a holosystolic murmur, coarse breath sounds, skin mottling, and an erythematous rash on her palms. Laboratory findings showed leukopenia, anemia, thrombocytopenia, hyponatremia, elevated creatinine, elevated liver enzymes, metabolic acidosis, and a peripheral blood smear with schistocytes, while imaging revealed diffuse nodular airspace opacities and extensive multifocal bilateral airspace opacities with air bronchograms.	Diagnosis	Infective endocarditis
			Diagnosis	It is more likely to lead to septic pulmonary emboli and pneumonia than left-sided endocarditis
			Disease characteristics	New cardiac murmur
Case 71	Internal Medicine	A 36-year-old woman presents with a worsening productive cough, subtle hearing loss, atopic dermatitis,	Diagnosis	Eosinophilic granulomatosis with polyangiitis

		bilateral otitis media, periorbital edema, nasal polyposis, expiratory wheezing, and mild crackles. Laboratory and imaging results show sinus opacification, diffuse interstitial changes on chest radiography, peribronchial thickening and patchy infiltrates on CT, moderate obstructive defect on pulmonary function testing, elevated eosinophil count, elevated ESR and CRP, positive myeloperoxidase ANCAs, and negative proteinase-3 ANCAs.	Disease characteristics	Necrotic lesions of the upper airway
			Treatment	Mepolizumab
Case 72	Internal Medicine	A 36-year-old woman presents with recurrent diarrhea, abdominal cramping, bloating, flatulence, and "foggy brain," with physical examination showing a healthy-appearing woman with no acute distress and normal abdominal findings. Laboratory tests reveal normal blood work and serology, while imaging and biopsy from EGD show normal duodenal histology except for mildly increased intraepithelial lymphocytes.	Diagnosis	Nonceliac gluten sensitivity
			Disease characteristics	Innate immune response
			Disease characteristics	Anxiety
Case 73	Minor	A 36-year-old woman presents with a one-month history of painful, progressive swelling of the right eye, complete external ophthalmoplegia, moderate proptosis, and blurred disc margins on funduscopic examination. Laboratory tests are unremarkable, and MRI reveals diffuse thickening and swelling of all extraocular muscles of the right orbit with proptosis, but a normal optic nerve and brain.	Diagnosis	Orbital pseudotumor
			Disease characteristics	Extraocular muscle tendon involvement
			Treatment	Corticosteroids
Case 74	Psychiatry	A 37-year-old man presents with agitation, irritability, and poor frustration tolerance following a motor vehicle collision, with physical examination showing elevated heart rate and blood pressure but no visible injuries. Laboratory tests are within normal limits except for cannabis on urine toxicology, and a CT head scan shows no abnormalities.	Diagnosis	Intellectual disability
			Diagnosis	Assessing the patient's adaptive functioning in conceptual, social, and practical domains
			Disease characteristics	Schizophrenia typically develops in late adolescence or adulthood, whereas ID manifests earlier in life
Case 75	Minor	A 38-year-old man presents with a 3-year history of a relapsing pruritic rash on his buttocks, characterized by linear plaques of coalescing erythematous papules with punctate erosions and sanguineous crusting, accompanied by moderate pruritus. Patch testing and swabs for viral and bacterial cultures are negative.	Diagnosis	Contact dermatitis
			Diagnosis	Rubber manufacturing accelerators
			Examination	Perform patch testing using samples of the patient's underwear
Case 76	Psychiatry	A 38-year-old woman presents with bad dreams, screaming, and thrashing during sleep, with a normal physical and mental status examination. Laboratory tests including a complete blood cell count, electrolyte tests, and a cholesterol panel are all within the reference range.	Diagnosis	Sleep terrors
			Disease characteristics	NREM sleep
			Treatment	After conservative measures have been attempted
Case 77	Internal Medicine	A 39-year-old man presents with severe, throbbing back pain radiating to the left buttock and upper back, with mild tenderness at T11-L1, normal strength and sensation in lower extremities, and an antalgic gait. Laboratory and imaging results include a normal anteroposterior lumbar radiograph and an MRI of the spine.	Diagnosis	Epidural abscess secondary to tuberculous spondylitis
			Disease characteristics	Cough
			Disease characteristics	It most commonly affects the cervical region
Case 78	Psychiatry	A 39-year-old man presents with diarrhea, weight loss, mood shifts, and a history of rapid speech and fluctuating mood, with normal physical examination findings. Laboratory tests including complete blood count, electrolyte levels, cholesterol panel, liver function tests, and a CT scan of the abdomen are normal, and toxicology screening is negative for barbiturates and opioids.	Diagnosis	A hypomanic episode
			Diagnosis	Bipolar disorder must include episodes defined as depression and mania or hypomania, whereas the mood symptoms in cyclothymic disorder do not qualify as depression or mania or hypomania
			Disease characteristics	Preliminary research suggests that bipolar disorder might be associated with changes in metabolism and microbial function
Case 79	Internal Medicine	A 39-year-old woman presents with fever, rash near the ear, facial twitches, tongue extrusions, orofacial dyskinesia, and a generalized seizure, along with	Diagnosis	Systemic lupus erythematosus mimicking tardive dyskinesia

		hypertension, pharyngeal erythema, oral ulcers, cervical adenopathy, bibasilar rales, and mild stocking-glove sensory changes. Laboratory results show mild leukocytosis, elevated erythrocyte sedimentation rate, normal creatinine and thyroid panel, and urinalysis with numerous WBCs, few red cell casts, and proteinuria, while a noncontrast head CT scan is normal.	Disease characteristics	All of the above
			Treatment	Combinations of dopamine and steroids
Case 80	Paediatrics	A 3-day-old boy presents with feeding intolerance, persistent bilious vomiting, hypotonia, hyporeactivity, dehydration, grayish skin coloration, abdominal distension, and bloody mucus on rectal examination. Laboratory and imaging results include an abdominal radiograph and an upper gastrointestinal contrast study, though specific findings from these tests are not provided in the text.	Diagnosis	Malrotation with midgut volvulus
			Examination	Ladd procedure
			Treatment	Upper gastrointestinal contrast study
Case 81	Paediatrics	A 3-month-old boy presents with severe failure to thrive, malnutrition, absence of subcutaneous fat, and bloated abdomen, with vital signs showing normal heart and lung function but low weight and head circumference below the 3rd percentile. Laboratory results reveal severe hypercalcemia, low normal phosphate, low alkaline phosphatase, elevated urine phosphoethanolamine, and high vitamin B6, while imaging shows irregular metaphyses, severe metaphyseal flaring, osteopenia, and bilateral hydronephrosis with possible uretero-pelvic junction obstruction.	Diagnosis	Hypophosphatasia
			Examination	Ultrasonography
			Disease characteristics	Hypercalcemia and decreased alkaline phosphatase
Case 82	Paediatrics	A 3-year-old boy presents with fever, cough, drowsiness, and moderately increased work of breathing with bilateral fine crackles on chest examination. Laboratory results show anemia with a hemoglobin level of 8.7 g/dL, leukopenia with a white blood cell count of 4.51×10^3 cells/ µL, and a chest radiograph reveals bilateral diffuse infiltrates.	Diagnosis	Burkholderia pseudomallei
			Disease characteristics	HIV
			Disease characteristics	B pseudomallei is fastidious and has special nutritional requirements
Case 83	Minor	A 40-year-old man presents with recurrent painful nodules on his buttocks, gluteal cleft, and thighs, with indurated plaques, erythematous nodules, sinus tracts, and scarring observed on examination. A punch biopsy reveals an epithelial-lined sinus tract with keratin debris and neutrophils, fibrotic dermis with mixed inflammatory infiltrate, and negative tissue cultures.	Diagnosis	Hidradenitis suppurativa
			Disease characteristics	The primary etiology of hidradenitis suppurativa is follicular occlusion, followed by follicular rupture and a brisk inflammatory response
			Treatment	Surgery should be considered, along with pharmacologic therapy, when developing an individualized treatment plan in most patients with hidradenitis suppurativa
Case 84	Internal Medicine	A 41-year-old woman presents with shortness of breath, hematuria, lymphadenopathy, fine crackles at lung bases, and mild bilateral pitting pedal edema, along with sinus tachycardia on ECG. Laboratory results show elevated creatinine, proteinuria, anemia, positive D-dimer, elevated ESR, and imaging reveals bilateral pleural effusions, enlarged lymph nodes, and a small pericardial effusion.	Diagnosis	Systemic lupus erythematosus
			Disease characteristics	In 3 months
			Examination	Lymphadenopathy
Case 85	Internal Medicine	A 42-year-old man presents with a firm, nontender right ear mass in the conchal bowl, with no overlying edema or erythema, and a history of left-sided cervical neck mass and Horner syndrome. Histologic examination of the excised mass shows macrophages with engulfed lymphocytes, positive staining for S100 and CD68, and large histiocytic cells with abundant amphophilic cytoplasm and vesicular nuclei.	Diagnosis	Rosai-Dorfman disease
			Disease characteristics	Histiocytes with engulfed lymphocytes
			Disease characteristics	Macrophage colony-stimulating factor
Case 86	Internal Medicine	A 42-year-old woman presents with chronic leg and lower back pain, with a physical examination showing a healthy-appearing, nonobese female without adenopathy, edema, or tenderness. Laboratory results are normal, including metabolic panel and urinalysis,	Diagnosis	Renal cell carcinoma
			Examination	Partial nephrectomy
			Treatment	CT scanning with and without contrast

		and imaging with spinal MRI and CT scans was performed following structural issue exclusion.		
Case 87	Internal Medicine	A 42-year-old man presents with worsening dyspnea over 2 years, decreased exercise tolerance, and physical examination reveals a fixed split S2 and prominent P2, with normal vital signs except for an O2 saturation of 92% on room air. Laboratory tests are normal, arterial blood gas shows respiratory alkalosis, chest X-ray reveals clear lung fields with prominent central pulmonary arteries, ECG shows tall R waves in V1 and inverted T waves in V1 to V4, II, III, and aVF, and CT angiogram of the chest is provided.	Diagnosis	Chronic thromboembolic pulmonary hypertension
			Examination	Pulmonary angiography
			Treatment	Pulmonary thromboendarterectomy
Case 88	Internal Medicine	A 42-year-old man presents with fatigue, intermittent low back pain worsening at night, mild conjunctival pallor, and minimal pedal edema, with no significant findings on physical examination of the back or joints. Laboratory results show normocytic normochromic anemia, elevated ESR, elevated serum creatinine and calcium, decreased serum albumin, mild proteinuria, and normal chest and thoracolumbar spine radiographs, with an MRI of the spine pending.	Diagnosis	Multiple myeloma
			Diagnosis	The initial treatment of choice for younger adults with MM is a combination of three different agents, including bortezomib; dexamethasone; and one other agent, such as an immunomodulatory drug
			Treatment	MM can be diagnosed without the presence of M protein on SPEP
Case 89	Internal Medicine	A 42-year-old woman presents with gradual-onset headache, frequent vomiting, and cognitive defects, with physical examination showing a reactive right pupil and no lesions in the left orbital fossa. Laboratory results reveal anemia, leukocytosis, elevated ESR, and MRI shows a hyperintense lesion in the posterior frontal region with surrounding edema, while histopathology indicates a tumor with marked nuclear pleomorphism and frequent mitotic activity.	Diagnosis	Metastatic melanoma
			Disease characteristics	A diligent clinical history is important for identifying an extracranial primary source
			Examination	Contrast-enhanced MRI
Case 90	Internal Medicine	A 42-year-old woman undergoing a renal transplant evaluation presents with normal vital signs and an unremarkable physical examination, including a symmetric breast examination without palpable lumps or discharge. Laboratory results show elevated blood urea nitrogen and creatinine levels, microcytic anemia with a hemoglobin of 8.2 g/dL, and a normal mammogram.	Disease characteristics	Dracunculiasis
			Disease characteristics	Development of deep venous thrombosis
			Disease characteristics	Small water fleas are the etiologic agent of the disease
Case 91	Internal Medicine	A 42-year-old Hispanic woman presented with fever, chills, intermittent dyspnea, productive cough, thick greenish nasal discharge, hyperemic conjunctiva, and bilateral harsh bronchial breathing with rales at the lung bases. Laboratory results showed elevated WBC count, neutrophilia, low albumin, elevated liver enzymes, and ANCA titer of 1:80, while imaging revealed left lower-lobe infiltrate with atelectasis, developing right lower-lobe infiltrate, and bilateral cavitary lesions on chest x-ray.	Diagnosis	Granulomatosis with polyangiitis
			Examination	Lung or kidney biopsy
			Disease characteristics	Subacute proptosis
Case 92	Internal Medicine	A 43-year-old woman presents with a 1-month history of painful, discolored toes, showing nonblanching purpuric lesions and superficial ulceration on physical examination. Laboratory tests including CBC, metabolic panel, inflammatory markers, and autoantibodies are normal, while a punch biopsy reveals perivascular lymphocytic infiltrate, superficial dermal hemorrhage, and lichenoid tissue reaction.	Diagnosis	Chilblains
			Diagnosis	All of the above
			Disease characteristics	Blistering with repeated cold exposure
Case 93	Internal Medicine	A 44-year-old man presents with weakness, fatigue, and slurred speech, with physical examination showing a minor forehead abrasion and nonfocal neurologic findings. Laboratory results reveal severe hyponatremia, hypoglycemia, and a pituitary adenoma on imaging, with subsequent tests indicating low cortisol, low testosterone, and elevated prolactin levels.	Diagnosis	Adrenal insufficiency
			Treatment	Normal (0.9%) saline infusion
			Disease characteristics	Stalk effect
Case 94	Minor	A 44-year-old man presents with progressive bilateral lower extremity numbness and weakness, occasional sharp back pain, and reduced pinprick sensation from the midcalf and below bilaterally, with an equivocal left	Diagnosis	Epidural cavernous hemangioma
			Examination	Extramedullary, increased signal on T2-weighted

		Babinski response. MRI reveals a posterior epidural mass at the T12-L1 level with homogeneous signal characteristics and no involvement of adjacent structures, while laboratory findings are normal.		images, and postcontrast enhancement
			Diagnosis	Epidural abscess
Case 95	Minor	A 44-year-old woman presents with a left occipital headache, photophobia, phonophobia, left ear hyperacusis, left facial asymmetry, and decreased sensation in the left V2/V3 distribution, along with 3+ bilateral patellar reflexes. Serum findings are unremarkable, and brain MRI with contrast and EMG were performed, showing findings similar to the provided image.	Diagnosis	Bell palsy
			Disease characteristics	Overuse of muscles of mastication
			Treatment	Steroids alone
Case 96	Internal Medicine	A 44-year-old woman with seropositive rheumatoid arthritis and allergy-induced asthma presents with progressive dyspnea and cough, with physical examination showing slightly distant breath sounds toward the lung bases. Laboratory tests are normal, while chest CT reveals right middle lobe and lingular bronchiectasis, bronchial wall thickening, and multiple predominantly solid airway-centered nodules, with bronchoscopy showing no bacterial growth or malignancy.	Diagnosis	Nontuberculous mycobacterial infection
			Disease characteristics	TNF inhibitor therapy should not be resumed, even after completion of antimicrobial therapy
			Treatment	In the United States, NTM pulmonary infections are more prevalent than tuberculosis
Case 97	Major Surgery	A 45-year-old man presents with gradual, nontender neck swelling over 6 months, with a fluctuant mass in the lower neck that moves with swallowing and tongue protrusion, and no cervical lymphadenopathy. Laboratory tests, including thyroid function, are normal, and imaging with ultrasound and CT of the neck is performed.	Diagnosis	Thyroglossal duct cyst
			Treatment	They can move with tongue protrusion
			Disease characteristics	Sistrunk procedure for cyst excision
Case 98	Internal Medicine	A 45-year-old man with a history of HIV presents with progressive shortness of breath, productive cough, fever, cachexia, and coarse bibasilar crackles on examination. Laboratory and imaging results show respiratory alkalosis, hypoxia, a CD4 count of 38 cells/μL, HIV-1 RNA of 1,710,000 copies/mL, and chest imaging reveals diffuse bilateral interstitial markings and ground-glass opacities.	Diagnosis	Pneumocystis jiroveci pneumonia
			Examination	Prophylaxis should be started and discontinued once the patient's CD4 count is above 200 cells/μL for 3 months
			Treatment	Chest x-ray
Case 99	Internal Medicine	A 45-year-old man presents with chronic sinus congestion, runny nose, occasional coughing, and mild headaches, with physical examination showing slightly pinkish and watery eyes and nose. Laboratory tests reveal elevated eosinophils at 8%, and a sinus radiograph shows mild congestion without structural abnormalities, while a throat culture shows no growth of organisms.	Diagnosis	New-onset allergies
			Examination	A trial of a newer-generation antihistamine
			Treatment	Sensitivity to one or more allergens on a pinprick test
Case 100	Minor	A 45-year-old woman presents with a 2-day history of a mildly pruritic rash that started in her axillae and groin, spreading to her body, accompanied by a fever of 102°F, fine crackles in the left lower lung field, and hundreds of nonfollicular pustules on erythematous bases. Laboratory results show elevated white blood cell count, neutrophils, eosinophils, CRP, and ESR, with blood cultures positive for Mycoplasma pneumoniae, and a punch biopsy revealing spongiform subcorneal pustules and perivascular neutrophil infiltration.	Diagnosis	Acute generalized exanthematous pustulosis
			Diagnosis	Fever
			Disease characteristics	Penicillin V
Case 101	Internal Medicine	A 45-year-old woman presents with confusion, sedation, numbness on the left side of her face, unusual sensation in her left eyeball, fast heartbeat, night sweats, and occasional hallucinations, with physical examination revealing mild distress, tachycardia, slight saccades motion impairment in the left eye, mild residual crackles in the lungs, and modest gait impairment. Laboratory results show a white blood cell count of 15.4×10^9/L and elevated blood urea nitrogen at 40 mg/dL, while imaging includes a normal chest x-ray, normal head CT scan, and ECG showing tachycardia.	Diagnosis	Medication toxicity
			Disease characteristics	CYP3A4
			Disease characteristics	Oseltamivir
Case 102	Internal Medicine	A 45-year-old woman presents with bleeding gums, bruises on forearms, petechial rash, and conjunctival hemorrhages following a febrile illness, with physical	Diagnosis	Dengue hemorrhagic fever
			Disease characteristics	Supportive care with fluid resuscitation, antipyretics,

		examination showing large bruises and gingival hemorrhage upon minor trauma. Laboratory results reveal anemia with hemoglobin at 8 g/dL, severe thrombocytopenia with a platelet count of $11\times10^3/\mu L$, leukopenia with a white blood cell count of $1.8\times10^3/\mu L$, and normal coagulation studies, while imaging and cultures are unremarkable.		and blood product replacement as needed
			Treatment	The patient is experiencing his second dengue infection with a different serotype
Case 103	Internal Medicine	A 46-year-old man presents with diffuse musculoskeletal pain, morning stiffness, joint swelling, tenderness, erythema, warmth, and a nonpruritic erythematous rash in the intragluteal cleft, along with scaly patches on the scalp. Laboratory results show a hemoglobin level of 11.8 g/dL with normal CBC, hepatic and renal chemistry, urinalysis, RF, and ANA, while radiographs reveal soft tissue swelling and an erosion at the left fourth DIP joint.	Diagnosis	Psoriatic arthritis
			Disease characteristics	Juxta-articular osteoporosis
			Disease characteristics	DIP joint and wrist arthritis
Case 104	Major Surgery	A 46-year-old man presents with fever, left-sided neck swelling extending to the anterior chest wall, drowsiness, confusion, dehydration, malnutrition, tachycardia, tachypnea, and a temperature of 104°F, with physical examination revealing reddish skin discoloration, crepitus, and pitting edema. Laboratory results show leukocytosis (WBC count of 35,000 cells/µL), mild anemia (hemoglobin 9 g/dL), elevated creatinine (2.5 mg/dL), and elevated liver enzymes, while imaging reveals soft tissue swelling with air on CT scan and no tuberculosis on chest radiograph.	Diagnosis	Necrotizing fasciitis
			Disease characteristics	Wound care and plastic surgery
			Treatment	> 25%
Case 105	Internal Medicine	A 47-year-old man with a history of alcohol-induced chronic pancreatitis presents with lightheadedness, fatigue, vague abdominal discomfort, profuse hematochezia, tachycardia, hypotension, pale conjunctiva, and dark-red stool on rectal examination. Laboratory results show severe anemia with a hemoglobin level of 3.9 g/dL, normal liver enzymes, and imaging reveals bleeding from the second part of the duodenum.	Diagnosis	Hemosuccus pancreaticus
			Disease characteristics	In cases of hemosuccus pancreaticus, active bleeding from the ampulla of Vater is rarely seen because of the intermittent nature of the bleeding
			Disease characteristics	Chronic pancreatitis
Case 106	Minor	A 47-year-old man presents with diplopia, mild bilateral lower-extremity tingling, moderately impaired gait, ataxic gait with a wide base, impaired lateral gaze of the right eye, 0+ patellar and Achilles reflexes, and diminished light touch sensation in the lower extremities. Laboratory and imaging results show normal brain MRI, normal glucose and WBC levels in cerebrospinal fluid with a mild increase in protein, a WBC count of 13,000 cells/µL, and normal comprehensive metabolic panel and urinalysis.	Diagnosis	Miller Fisher syndrome
			Examination	Anti-GQ1b
			Disease characteristics	Horizontal gaze palsy
Case 107	Internal Medicine	A 47-year-old man presents with progressive dyspnea, intermittent productive cough, and painful weepy cutaneous nodules on the right forearm, with physical examination showing decreased air entry and mild hepatomegaly. Laboratory results reveal mild elevation of aminotransferases, positive C-ANCA, low serum alpha-1 antitrypsin levels, and pulmonary function tests indicating reduced FEV1 and DLCO, while imaging shows hyperlucent areas in bilateral lower lobes.	Diagnosis	AAT deficiency (AATD)
			Examination	AAT phenotype determined by IEF
			Treatment	All of the above
Case 108	Internal Medicine	A 48-year-old man presents with dyspnea, cyanotic skin, facial and arm swelling, hoarseness, right upper eyelid ptosis, and reduced breath sounds in the right lung. Laboratory tests are normal, ECG shows no acute changes, and imaging includes a chest radiograph and CT scan.	Diagnosis	Superior vena cava syndrome
			Diagnosis	Small cell carcinoma
			Treatment	Chemotherapy alone
Case 109	Internal Medicine	A 48-year-old man presents with intermittent fever, pain, and tender nodules on his thumbs, along with weakness, malaise, diarrhea, weight loss, anorexia, and vomiting, and physical examination shows pale nail beds and normal vital signs except for elevated blood pressure. Laboratory results reveal hemoglobin of 12.2 g/dL, hematocrit of 37%, white blood cell count of 6.7 $\times10^3/\mu L$, platelet count of 150 $\times10^3/\mu L$, creatinine of 1.15 mg/dL, BUN of 13 mg/dL, and an erythrocyte	Diagnosis	Infectious endocarditis
			Disease characteristics	Gentamicin and vancomycin
			Treatment	Staphylococcus aureus

		sedimentation rate of 47 mm/hr, with normal electrocardiography findings.		
Case 110	Internal Medicine	A 48-year-old man with cirrhosis presents with sudden abdominal distension, periumbilical ecchymosis, scleral icterus, and intermittent disorientation. Laboratory results show a significant drop in hematocrit, thrombocytopenia, elevated INR, high bilirubin, low potassium, and an abdominal CT reveals a nodular liver, splenomegaly, and dense hemorrhagic ascites.	Disease characteristics	Portal hypertension
			Disease characteristics	All of the above
			Disease characteristics	Paracentesis can be repeated at a remote site; persistently hemorrhagic fluid is consistent with intraperitoneal hemorrhage
Case 111	Minor	A 49-year-old man presents with progressive memory loss, gait impairment, myoclonic jerks, dysarthria, and confusion, along with spasticity, hyperreflexia, and extensor plantar responses on examination. Laboratory tests are unremarkable, EEG shows diffuse low voltage with slowing and polyspikes, and MRI reveals gross cerebral atrophy with asymmetric diffusion restriction in the cerebral cortex and left basal ganglia.	Diagnosis	Creutzfeldt-Jakob disease
			Disease characteristics	Zoonotic
			Disease characteristics	PRNP gene on chromosome 20
Case 112	Minor	A 49-year-old man with a history of cervical spine fracture, diabetes, and hearing loss presents with increasing pain at the site of a previous right femoral fracture, showing diffuse tenderness, mild hip flexion limitation, severe knee flexion limitation, muscle stiffness, and atrophy. Laboratory results reveal a slightly elevated alkaline phosphatase level, normal erythrocyte sedimentation rate, and normal complete blood count, while radiographs of the femur and pelvis are provided.	Diagnosis	Benign osteopetrosis
			Diagnosis	Plain radiography
			Examination	Bone marrow failure
Case 113	Paediatrics	A 4-year-old girl presents with frequent urination, excessive thirst, enuresis, fatigue, irritability, pallor, abdominal pain, and multiple episodes of emesis, with physical examination revealing severe dehydration, somnolence, dry mucosae, sunken eyes, and delayed capillary refill. Laboratory results show a venous pH of 6.95, glucose level of 877 mg/dL, hematocrit of 50%, venous pCO2 of 5 mm Hg, venous bicarbonate of <5 mEq/L, anion gap of 34 mmol/L, and serum osmolality of 312 mosm/kg, leading to a diagnosis of severe diabetic ketoacidosis.	Diagnosis	Deep vein thrombosis
			Disease characteristics	Placement of a CVC
			Disease characteristics	Vascular injury
Case 114	Internal Medicine	A 50-year-old man presents with hematemesis, recurrent epigastric pain, mild jaundice, and hepatosplenomegaly, with a blood pressure of 90/60 mm Hg and pulse of 100 beats/min. Laboratory results show leukopenia, anemia, thrombocytopenia, elevated liver enzymes, and hyperbilirubinemia, while an abdominal ultrasound reveals hepatosplenomegaly without fluid collection.	Diagnosis	Portal vein thrombosis
			Disease characteristics	35% of patients
			Treatment	3 months
Case 115	Minor	A 50-year-old man with AIDS presents with progressive right leg weakness, burning pain, cramps, and paresthesia in the left leg, with physical examination showing 0/5-1/5 strength in the right leg and decreased proprioception and vibratory sense in the right foot. Laboratory results reveal leukopenia, a low CD4 count of 4 cells/mm³, a high HIV viral load, positive serum cytomegalovirus antibodies, and MRI of the thoracic spine shows an abnormal T2-weighted hyperintense signal at the T3-4 level predominantly on the right side.	Diagnosis	Brown-Sequard syndrome
			Disease characteristics	CSF PCR
			Examination	Ipsilateral weakness; loss of vibratory sense and of proprioception; contralateral loss of pain and of temperature sensation
Case 116	Internal Medicine	A 50-year-old woman presents with persistent cough, dyspnea, moderate sputum production, occasional mild hemoptysis, frequent heavy epistaxis, bilateral rhonchi, mild wheezing, and multiple superficial nonbleeding vessels on the nasal septum. Laboratory results show an increased white blood cell count of 15.6×10³/µL and microcytic, hypochromic anemia with a hemoglobin of 9.2 g/dL, while a chest x-ray reveals no effusions or infiltrates.	Diagnosis	Osler-Weber-Rendu syndrome
			Disease characteristics	Epistaxis
			Treatment	It is mostly supportive
Case 117	Internal Medicine	A 51-year-old man with HIV presents with right suprapubic pain radiating to the testicles, nausea,	Diagnosis	Waldenström macroglobulinemia

		vomiting, neck pain, dizziness, swollen lymph nodes, bone pain, fatigue, ischuria, dysuria, and physical examination reveals tachycardia, hepatomegaly, and tenderness in the left lower quadrant and suprapubic area. Laboratory and imaging results show normocytic anemia, thrombocytopenia, serum monoclonal M gammopathy, elevated kappa and lambda free light chains, monoclonal bands in urine, elevated serum viscosity, and a bone marrow biopsy revealing plasmacytoid cells positive for CD138 with a monoclonal population of B cells.	Diagnosis	Organomegaly
			Disease characteristics	Non-Hodgkin lymphoma
Case 118	Internal Medicine	A 52-year-old man with a history of alcoholism presents with severe soreness, mandibular deviation, a large opening at the left retromolar area, purulent discharge, weight loss, nocturnal fever, and palpable cervical lymph nodes. Radiographic examination shows an extensive radiolucent area in the mandible, laboratory findings reveal elevated ESR and a positive PPD test, and microbial culture of the discharge is positive for acid-fast bacilli after 5 weeks.	Diagnosis	Tuberculous osteomyelitis
			Disease characteristics	TB of the maxilla
			Treatment	Follow-up should be individually tailored to the response to therapy and overall health of the patient
Case 119	Minor	A 52-year-old man presents with headache, nausea, blurry vision, confusion, and drowsiness, with physical examination revealing obesity, drowsiness, dry oropharynx, and retinal hemorrhages and exudates without papilledema. Laboratory tests show no significant findings, and imaging includes an ECG.	Diagnosis	Hypertensive emergency
			Treatment	Nitroglycerin
			Treatment	Labetalol
Case 120	Internal Medicine	A 52-year-old man presents with fatigue, night sweats, weight loss, and moderate splenomegaly, with physical examination revealing a palpable spleen 7 cm below the left costal margin. Laboratory results show leukocytosis with a WBC count of 42,000 cells/μL, anemia with hemoglobin at 10.8 g/dL, and elevated lactate dehydrogenase at 690 IU/L, while peripheral smear and bone marrow findings are provided in images.	Diagnosis	Chronic myeloid leukemia
			Treatment	Bosutinib has been approved by the US Food and Drug Administration (FDA) for first-line use in chronic-phase CML
			Treatment	Ponatinib
Case 121	Internal Medicine	A 53-year-old man with a history of multiple myeloma presents with a 1-week history of melena, hematemesis, and lethargy, and physical examination shows he is clinically well with no active GI bleeding but evidence of melena on rectal examination. Laboratory results reveal anemia with a hemoglobin level of 8.5 g/L, low mean corpuscular volume indicating iron deficiency, mild dehydration, and normal liver function tests, while esophagogastroduodenoscopy is performed after stabilization.	Diagnosis	Extraosseous spread of multiple myeloma
			Disease characteristics	Sickle cell anemia
			Disease characteristics	The incidence is higher in farmers and horticulturists.
Case 122	Internal Medicine	A 53-year-old woman presents with diffuse abdominal pain, visual blurriness, xerostomia, bilateral ptosis, dysphonia, dysarthria, and bilateral cranial nerve VI paresis, along with sinus tachycardia and later bradycardia. Laboratory tests are normal, abdominal CT shows dilated loops of bowel without obstruction, brain CT is normal, and arterial blood gas analysis reveals moderate respiratory acidosis.	Diagnosis	Foodborne botulism
			Disease characteristics	It is indicated for infants up to 1 year of age who have a diagnosis of botulism
			Treatment	Neurotoxins A, B, and E
Case 123	Internal Medicine	A 53-year-old woman presents with worsening low back pain and an occasional dry cough, with physical examination showing normal vital signs and no acute distress. Laboratory results are within normal ranges except for elevated alkaline phosphatase, while imaging reveals hyperinflated lungs with flattened diaphragms and multiple destructive lesions in the vertebral bodies at T8, T9, L1, and L3.	Diagnosis	Metastatic cancer
			Examination	Alpelisib
			Treatment	Bilateral breast MRI
Case 124	Internal Medicine	A 55-year-old man presents with recurrent sharp right flank pain radiating to the groin, hematuria, and mild costovertebral angle tenderness on the right side. Laboratory results show hematuria with normal creatinine, elevated serum ionized calcium, increased 24-hour urinary calcium, and ultrasound reveals a 13 mm stone in the right renal pelvis, with parathyroid scintigraphy performed.	Diagnosis	Parathyroid adenoma
			Disease characteristics	Solitary adenoma
			Diagnosis	Hypocalcemia
Case 125	Minor		Diagnosis	Rosacea (papulopustular)

		A 55-year-old woman presents with a 6-month history of dome-shaped erythematous to violaceous papules and superficial pustules on her cheeks, accompanied by occasional warmth, burning, and flushing, with no abnormalities on other skin areas. Laboratory results show a complete blood count and complete metabolic panel within the reference range.	Disease characteristics	It involves nodular hypertrophy of sebaceous glands
			Treatment	Treat the patient with doxycycline 100 mg twice daily
Case 126	Internal Medicine	A 55-year-old woman presents with severe, progressive shortness of breath, chest pressure, wheezing, labored respirations, inability to speak in full sentences, bilateral crackles, and 1+ pitting edema, with vital signs showing tachycardia and hypertension. Laboratory results reveal a pH of 7.27, partial oxygen pressure of 54 mm Hg, partial carbon dioxide pressure of 63 mm Hg, and chest radiography shows pulmonary edema, while ECG indicates a tachycardic rhythm with a preexisting left bundle branch block.	Diagnosis	Atrial flutter
			Treatment	Coronary artery catheterization
			Treatment	Furosemide
Case 127	Internal Medicine	A 56-year-old man presents with exertional dyspnea and difficulty arising from a chair, with physical examination showing decreased breath sounds on the right lung base and symmetric proximal muscle weakness. Laboratory and imaging results reveal elevated creatinine kinase at 1488 U/L, right lower-lobe consolidation on chest CT, oropharyngeal dysphagia on swallowing fluoroscopy, and MRI of the left thigh showing extensive edema, with muscle biopsy indicating scattered necrotic and regenerating fibers and scant inflammatory infiltration.	Diagnosis	Chronic GVHD (cGVHD)-associated myositis
			Disease characteristics	Steroids
			Treatment	Both proximal and distal muscles can be affected
Case 128	Minor	A 56-year-old woman presents with worsening paresthesias in her arms, difficulty lifting boxes, exhaustion when climbing stairs, occasional coughing and choking while eating, and physical examination reveals slightly decreased strength in her biceps, triceps, and proximal lower extremities, with slightly diminished reflexes. Electrodiagnostic studies show small compound muscle action potentials with decreased amplitude, which increase with repetitive stimulation.	Diagnosis	Lambert-Eaton myasthenic syndrome
			Examination	Bronchoscopy
			Treatment	Plasmapheresis
Case 129	Internal Medicine	A 56-year-old woman with a history of pulmonary sarcoidosis presents with progressive dyspnea, decreased exercise tolerance, and physical examination findings of a loud P2 and decreased air entry at lung bases, with normal vital signs except for low oxygen saturation. Laboratory tests are normal, blood gas shows mild respiratory alkalosis with hypoxemia, chest X-ray reveals bilateral hilar adenopathy, and pulmonary function tests indicate restrictive lung disease with unchanged results over six months.	Diagnosis	Pulmonary hypertension
			Examination	Long-term antibiotic therapy
			Treatment	Right-heart catheterization
Case 130	Internal Medicine	A 57-year-old man presents with fever, tachycardia, and ongoing gum bleeding, with physical examination showing normal skin and lymph nodes. Laboratory findings reveal pancytopenia with severe thrombocytopenia, normal bone marrow cytogenetics, elevated anticardiolipin IgM, and normal imaging studies, leading to a diagnosis of immune thrombocytopenia treated with corticosteroids and eltrombopag.	Diagnosis	Autoimmune myelofibrosis
			Disease characteristics	It is a benign condition and has a favorable prognosis
			Disease characteristics	Erythroid and megakaryocytic hyperplasia
Case 131	Minor	A 57-year-old man presents with gradually worsening lower-extremity pain, fatigue with ambulation, diminished sensation in a stocking distribution, and a wide-based gait, along with diminished but palpable posterior tibial and dorsalis pedis pulses. Imaging reveals mild to moderate degenerative scoliosis, loss of lumbar lordosis, disc height loss, grade 1 anterolisthesis of L2-L3, and severe central canal stenosis at L2-3 and L3-4.	Diagnosis	Neurogenic intermittent claudication (NIC)
			Disease characteristics	Lower-extremity pain and/or fatigue that is worsened by walking and improved by sitting
			Treatment	Short-term improvement in walking tolerance as a result of decreased radicular pain from inflamed lumbosacral nerve roots
Case 132			Diagnosis	Isolated bone plasmacytoma

	Internal Medicine	A 57-year-old man presents with vague left shoulder pain, mild tenderness over the left scapula, and full range of motion, with normal vital signs and no skin lesions. Laboratory studies, including CBC and serum electrolytes, are normal, while imaging reveals multiple well-circumscribed lytic lesions in the scapula, and a biopsy shows a clear margin with a narrow zone of transition to normal bone.	Diagnosis	High doses (> 4500 cGy) of local radiation therapy
			Disease characteristics	Plain radiography showing a single destructive bone lesion, in addition to a fine-needle aspiration or core biopsy revealing infiltration by plasma cells
Case 133	Internal Medicine	A 57-year-old woman presents with a 3-day history of colicky lower-abdominal pain and bloody diarrhea, with physical examination revealing mild abdominal distension, positive bowel sounds, and strongly guaiac-positive stool. Laboratory results show low hemoglobin, low hematocrit, low potassium, elevated amylase and lipase, and a normal abdominal contrast-enhanced CT scan.	Diagnosis	Intussusception
			Disease characteristics	Adenocarcinoma
			Treatment	Surgical resection without preoperative reduction
Case 134	Internal Medicine	A 58-year-old man with a history of hypertension, chronic hepatitis C, and end-stage renal disease presents with weakness, mild bilateral knee pain, and a rash characterized by scattered erythematous, hyperpigmented papules on both legs. Laboratory results show elevated creatinine levels (rising from 2.1 to 3.1 mg/dL), supratherapeutic tacrolimus levels, elevated liver enzymes, positive antinuclear antibody, high HCV RNA, low C4, and a renal biopsy was performed.	Diagnosis	Cryoglobulinemia
			Treatment	Weakness, purpura, and arthralgia
			Disease characteristics	DAA therapy
Case 135	Internal Medicine	A 59-year-old man with a history of hypertension develops sudden-onset shortness of breath on postoperative day 4 after a subtotal colectomy, with physical examination showing a regular pulse of 110 beats/min, respiratory rate of 30 breaths/min, oxygen saturation of 92% on room air, and mildly decreased breath sounds at the right lung base. Laboratory findings are normal, arterial blood gas shows pH 7.45, pCO2 32 mm Hg, pO2 62 mm Hg, and a chest x-ray reveals bibasilar subsegmental atelectasis, leading to intubation for hypoxemic respiratory distress.	Diagnosis	Massive pulmonary embolism
			Treatment	Subcutaneous LMWH for 1 month
			Treatment	IVC filter insertion
Case 136	Internal Medicine	A 59-year-old woman with a history of rheumatoid arthritis presented with a pruritic, painful rash on her elbows, nonblanching nodules, ulcerations, and dry gangrene, later developing febrile altered mental status. Laboratory results showed positive rheumatoid factor, elevated ESR of 91 mm/hr, CRP of 17.1 mg/L, WBC count of $13.1\times10^9/L$, hemoglobin of 9.6 g/dL, and hematocrit of 30.8%, with wound biopsy revealing osteomyelitis.	Diagnosis	Rheumatoid vasculitis
			Disease characteristics	Corticosteroids and immunosuppressants
			Treatment	Immune complex (antigen antibody) mediated
Case 137	Paediatrics	A 5-year-old girl presents with an 8-month history of progressive malodorous nasal discharge, partial nasal obstruction, sleep disturbances, and snoring, with physical examination showing bilateral nonpurulent nasal discharge and normal tympanograms. Laboratory results reveal a culture positive for Pseudomonas aeruginosa, and a CT scan of the nose and paranasal cavities is obtained.	Diagnosis	Rhinolithiasis
			Examination	Endoscopic examination under general anesthesia
			Diagnosis	This is formed around a foreign body in the nose, and the findings on CT are very typical for this
Case 138	Paediatrics	A 5-year-old girl presents with a 2- to 3-week history of high fever, nonproductive cough, occasional posttussive emesis, and right shoulder pain, with physical examination showing mild cervical adenopathy and clear lungs. Chest radiograph reveals right upper-lobe opacification and increased lucency, with CT of the chest clarifying the diagnosis.	Diagnosis	Abscess
			Treatment	Vancomycin plus piperacillin/tazobactam
			Examination	Obtain CT of the chest
Case 139	Paediatrics	A 5-year-old girl presents with severe headache, projectile vomiting, left-sided hemiparesis, hemianesthesia, pallor, icterus, and a systolic murmur, with a palpable spleen and stupor alternating with irritability. Laboratory findings show hemoglobin of 6.5 g/dL, WBC count of 24,000 cells/mcL, platelet count of 500,000 cells/mcL, normal PT/PTT and routine chemistry, with blood smear indicating sickle cells,	Diagnosis	Sickle cell anemia with stroke
			Examination	TCD ultrasonography
			Treatment	Treatment is lifelong

		HPLC consistent with sickle cell disease, and brain CT showing cerebral infarction.		
Case 140	Internal Medicine	A 60-year-old man presents with crampy, left-sided abdominal pain, fever, and tenderness in the left lower quadrant with voluntary guarding, but no rebound tenderness. Laboratory results show an elevated white blood cell count with neutrophil predominance, unremarkable urinalysis, and abdominal CT imaging reveals findings consistent with diverticulitis.	Diagnosis	Acute diverticulitis
			Examination	MRI of the abdomen
			Disease characteristics	All of the above
Case 141	Minor	A 60-year-old woman presents with abnormal behavior, irritability, disorientation, forgetfulness, unsteadiness, incontinence, and intermittent myoclonic jerks, with physical examination showing slurred speech, brisk reflexes, and impaired memory. Laboratory tests, imaging, and CSF analysis are normal, while EEG shows periodic sharp wave complexes.	Diagnosis	Creutzfeldt-Jakob disease
			Disease characteristics	Sporadic Creutzfeldt-Jakob disease
			Disease characteristics	It is increased by repeated studies
Case 142	Internal Medicine	A 61-year-old man presents with crampy abdominal pain, diarrhea with blood, fatigue, and mild shortness of breath, and physical examination reveals pallor, tachycardia, abdominal distension with rebound tenderness, and pitting edema. Laboratory results show anemia, leukocytosis, thrombocytosis, elevated inflammatory markers, low albumin, and flexible sigmoidoscopy reveals erythematous, friable colonic mucosa with ulcerations and crypt abscesses, without granulomas.	Diagnosis	Inflammatory bowel disease
			Disease characteristics	Pseudopolyps
			Disease characteristics	High-grade fever
Case 143	Major Surgery	A 61-year-old woman presents with severe upper abdominal pain radiating to the right shoulder, nausea, syncope, and diffuse abdominal tenderness with guarding and rebound tenderness in the right upper quadrant. Laboratory tests show borderline low hemoglobin and normal aminotransferase, bilirubin, and lipase levels, while imaging includes unremarkable chest and abdominal radiographs and a CT of the abdomen and pelvis.	Diagnosis	Spontaneous rupture of hepatoma
			Diagnosis	All of the above
			Diagnosis	Alcoholic cirrhosis
Case 144	Internal Medicine	A 61-year-old woman with a history of diabetes, hypertension, and obesity presents with nonhealing venous ulcers and new black eschars on her thighs, surrounded by erythema and induration, without fever or systemic symptoms. Laboratory results show anemia with a hemoglobin level of 10 g/dL, elevated platelet count of 492×10^3 cells/µL, normal metabolic panel, elevated ESR at 80 mm/hr, and biopsy indicating acute and chronic inflammation with small-vessel microthrombi at the ulcer base.	Diagnosis	Cryoglobulinemia
			Disease characteristics	Underlying lymphoproliferative disorder
			Disease characteristics	Eczematous patches
Case 145	Internal Medicine	A 61-year-old woman with a history of breast cancer and multiple sclerosis presents with painful constipation, smaller-caliber stools, and urinary difficulties, with a physical examination revealing a nontender, solid mass obstructing the rectal outlet. Colonoscopy shows a mass at the rectal sigmoid junction causing significant obstruction, and imaging reveals hypermetabolic activity in the pelvis with rectal wall thickening and hydronephrosis.	Diagnosis	Metastatic lobular breast cancer
			Examination	Biopsy is necessary to determine the specific type of treatment needed
			Treatment	Chemoradiation
Case 146	Minor	A 62-year-old man presents with a 1-year history of involuntary movements of the left half of the face, associated with pulsatile tinnitus and vertigo, and physical examination reveals periodic facial movements with intact cranial nerves and grade II hypertensive retinopathy. Laboratory tests show normal blood counts and organ function with trace proteinuria and abnormal lipid profile, while imaging reveals a severely tortuous and dilated vertebrobasilar system and chronic microvascular angiopathy without arteriovenous malformation.	Diagnosis	Cranial arterial dolichoectasia
			Disease characteristics	Seventh
			Disease characteristics	Meningitis
Case 147	Internal Medicine	A 62-year-old man presents with progressively increasing shortness of breath, fatigue, anorexia, weight loss, nonproductive cough, and left thigh pain, with physical examination revealing pallor, cachexia, tachypnea, reduced air entry at the right lung base, and	Diagnosis	Bronchogenic carcinoma
			Examination	Transbronchial biopsy
			Disease characteristics	Metastatic disease

		bilateral coarse crepitations. Laboratory results show anemia with a hemoglobin level of 9.8 g/dL, and imaging reveals abnormalities on chest radiograph and CT scan.		
Case 148	Minor	A 62-year-old man with a history of hypertension, coronary artery disease, and end-stage renal disease presents with worsening right shoulder pain, swelling over the acromioclavicular joint, and impaired shoulder movement. Radiograph shows a high-riding humerus and advanced humeral head deformity with a large soft-tissue mass, while laboratory tests reveal normal C-reactive protein, erythrocyte sedimentation rate, and complete blood count.	Diagnosis	AC joint cyst
			Examination	Right shoulder hemiarthroplasty
			Treatment	MRI
Case 149	Internal Medicine	A 64-year-old man presents with left-sided arm and leg weakness, difficulty walking, and decreased sensation on the right side, with physical examination showing 3/5 strength in the left extremities and coordination difficulties. Laboratory tests are normal, and imaging reveals lesions in the brain.	Diagnosis	Lung cancer metastasis to the brain
			Disease characteristics	Some treatments for Hodgkin lymphoma are associated with a significantly increased risk for lung cancer
			Treatment	Palliative external-beam radiation is recommended for patients who present with diffuse distant metastasis to the brain
Case 150	Internal Medicine	A 64-year-old man presents with right lower back pain, normal vital signs, and a physical examination showing no distress or abnormalities. Laboratory tests are normal, while MRI reveals a complex lytic lesion in the right iliac wing, and PET scan shows a heterogeneous mass in the liver and a lesion in the right iliac wing with elevated standardized uptake values.	Diagnosis	Metastatic breast cancer
			Disease characteristics	Younger age (<30 years)
			Disease characteristics	Estrogen receptor positive, HER2-negative
Case 151	Internal Medicine	A 64-year-old man presents with throat swelling, difficulty swallowing, drooling, swollen lips, tongue, and throat, and features of hypertensive heart disease in biventricular failure, requiring a tracheostomy. Laboratory results show elevated creatinine, ammonia, ALT, AST, lactic acid, white blood cell count, potassium, and glucose levels, with normal C1-INH and complement assays.	Diagnosis	Drug-induced angioedema
			Disease characteristics	Black women
			Disease characteristics	A history of previous angioedema, even if the cause is not known, is considered a contraindication for lisinopril
Case 152	Minor	A 64-year-old man presents with decreased motivation, fatigue, frequent constipation, and a slow, shuffling gait, along with a flat affect, dry skin, and moderate cogwheel rigidity in both upper extremities. Laboratory results show a normal complete blood cell count and a glycosylated hemoglobin level of 5.6%.	Diagnosis	Parkinson's disease
			Disease characteristics	Bradykinesia is associated with neurodegeneration in the basal ganglia, sensory areas of the cerebral cortex, and cerebellum
			Disease characteristics	The neurodegenerative process of Parkinson's disease may cause depression, and living with the symptoms of Parkinson's disease may exacerbate depression
Case 153	Minor	A 64-year-old woman presents with blurry vision, a wide-based gait, and off-balance heel-to-toe walking, along with moderate rigidity of the upper extremities and a positive Babinski sign on the left side. Laboratory and imaging results are not provided in the case details.	Diagnosis	Progressive supranuclear palsy
			Disease characteristics	Pseudobulbar affect
			Treatment	Discontinue carbidopa-levodopa
Case 154	Internal Medicine	A 64-year-old woman presents with shortness of breath at rest, nonproductive cough, lower extremity swelling, jugular venous distention, bilateral crackles, an S3 gallop, and moderate bilateral pitting edema. Laboratory and imaging results show elevated BNP at 4,237 pg/mL, troponin I at 0.42 ng/mL, ECG with new T-wave inversion, chest X-ray indicating moderate pulmonary edema and bilateral pleural effusion, echocardiography revealing hypokinesis with an	Diagnosis	Acute-on-chronic heart failure secondary to Takotsubo cardiomyopathy
			Treatment	Furosemide
			Treatment	Warfarin

		ejection fraction of 30-35%, and coronary angiography showing mild coronary artery obstructions.		
Case 155	Internal Medicine	A 64-year-old woman presents with symptomatic anemia, dyspnea on exertion, chest pain, and recurrent painless rectal bleeding, with physical examination revealing pallor, cachexia, tachypnea, orthostatic changes, and dark red blood in the rectum. Laboratory results show severe anemia with a hemoglobin level of 3.6 g/dL, and imaging via anterograde single-balloon enteroscopy reveals active bleeding from a visible vessel 160 cm from the incisors, with normal surrounding mucosa.	Diagnosis	Dieulafoy lesion
			Disease characteristics	1%-5%
			Disease characteristics	The most common presenting symptom is melena alone
Case 156	Psychiatry	A 65-year-old man presents with delusional thoughts, believing he is a prisoner of war, and exhibits poor insight and dangerous decision-making, with a physical examination showing a BMI of 35.9 kg/m² and blood pressure of 138/104 mm Hg. Laboratory results reveal a total bilirubin level of 1.8 μmol/L, blood urea nitrogen of 21 mg/dL, creatinine of 1.5 mg/dL, and a white blood cell count of 12.9 x 10^9/L, while an ECG shows marked left axis deviation and incomplete right bundle branch block, and a CT scan of the head is unrevealing.	Diagnosis	Schizophrenia
			Disease characteristics	Rapid symptom onset
			Disease characteristics	Increased cholinergic activity in the striatum
Case 157	Internal Medicine	A 65-year-old man presents with worsening dyspnea on exertion, chest tightness, wheezing, and coughing, accompanied by mild respiratory distress, moderate retractions, pursed-lipped breathing, diminished breath sounds, prolonged expiratory phase, faint expiratory wheezes, distant heart sounds, and trace ankle edema. ECG findings reveal rightward axis deviation, P-pulmonale, and low QRS voltage.	Diagnosis	Chronic obstructive pulmonary disease (COPD)
			Disease characteristics	Ventricular tachycardia
			Disease characteristics	Left ventricular hypertrophy
Case 158	Internal Medicine	A 65-year-old man presents with a hugely distended abdomen, decreased appetite, nausea, mild diffuse abdominal pain, and difficulty breathing, with physical examination showing dry oral mucosa and tympanic bowel sounds. Laboratory results reveal leukocytosis, elevated blood urea nitrogen, and creatinine, while imaging shows significant abdominal distension with findings suggestive of bowel obstruction.	Diagnosis	Acute colonic pseudo-obstruction
			Treatment	Neostigmine
			Treatment	Bowel rest, intravenous fluids replacement, and correction of electrolytic abnormalities
Case 159	Minor	A 65-year-old man presents with sudden, severe right-sided retro-orbital pain, diplopia, right eyelid drooping, and facial sensory loss, with examination revealing right-sided partial ptosis and restricted eye movements. Laboratory tests, including CBC, ESR, liver and renal function, and imaging such as MRI of the brain and orbit, are normal, with negative results for autoimmune and infectious markers.	Diagnosis	Tolosa-Hunt syndrome
			Disease characteristics	CN XI
			Disease characteristics	Unknown
Case 160	Internal Medicine	A 65-year-old man with uncontrolled hypertension, diabetes, and a history of infective endocarditis presents with shortness of breath, livedo reticularis, necrotic ulcers, inspiratory rales, a systolic murmur, and 2+ foot swelling. Laboratory results show anemia, elevated potassium, low bicarbonate, high blood urea nitrogen, elevated creatinine, low calcium, high phosphate, elevated parathyroid hormone, 2+ proteinuria, and renal ultrasound reveals a single right kidney measuring 9.6 cm.	Diagnosis	Diabetic kidney disease
			Treatment	All of the above
			Disease characteristics	Intravenous iron (to achieve saturation > 20%), then epoetin alfa if hemoglobin is < 10 g/dL
Case 161	Internal Medicine	A 65-year-old woman presents with increasing confusion, decreased coordination, visual agnosia, and a left facial droop, along with decreased breath sounds in the right lower lobe and a systolic ejection murmur. Laboratory and imaging results reveal a sodium level of 133 mEq/L, elevated white blood cell count, hemoglobin of 8.6 g/dL, troponin of 3.94 ng/mL, a 5.5-cm mass in the right lower lobe with pleural effusion, left and right occipital lobe infarcts, and valvular masses on echocardiography.	Diagnosis	Nonbacterial thrombotic endocarditis
			Treatment	Unfractionated heparin
			Treatment	Surgical excision of the vegetation
Case 162	Psychiatry	A 66-year-old man presented with a Colles fracture, tremor, diaphoresis, skin picking, auditory hallucinations, and agitation, with physical examination	Diagnosis	Alcohol withdrawal delirium
			Treatment	They occur only in the first 12-48 hours

		revealing tremors, dry mucous membranes, ecchymoses, spider angiomata, irregular heart rhythm, and suspected mild hepatomegaly. Laboratory results showed hypokalemia, low magnesium, elevated bicarbonate, high blood urea nitrogen, elevated liver enzymes, and atrial fibrillation with tachycardia on EKG, and he experienced a tonic-clonic seizure on the third day of admission.	Disease characteristics	Diazepam therapy
Case 163	Minor	A 66-year-old woman presents with decreased vision in the right eye, distortion, and a history of central scotoma in the left eye, with examination showing 20/80 vision in the right eye, 20/400 in the left, mild nuclear sclerotic cataracts, and subretinal hemorrhage. Imaging reveals increased hyperfluorescence with leakage in the right macula, late staining of subretinal fibrosis in the left eye, and optical CT showing elevation of the retinal pigment epithelium and disruption of the photoreceptor layer in the right eye, with subretinal fibrosis and cystic changes in the left eye.	Diagnosis	Choroidal neovascularization associated with angioid streaks
			Disease characteristics	Osteosarcoma
			Disease characteristics	VEGF
Case 164	Internal Medicine	A 67-year-old man presents with palpitations, lightheadedness, and transient loss of consciousness, with a normal physical examination and cardiovascular evaluation. Laboratory results show normal findings except for mildly elevated bilirubin, and imaging reveals mild left ventricular hypertrophy with impaired ventricular relaxation, while ambulatory cardiac monitoring indicates episodes of arrhythmia followed by sinus rhythm and dizziness.	Diagnosis	Cardiac arrhythmia
			Treatment	A permanent pacemaker implant
			Treatment	Switch to oral anticoagulation (OAC)
Case 165	Internal Medicine	A 67-year-old man presents with waxing and waning left-sided low back pain, chronic heartburn, and a normal physical examination, except for red blood cells in the urine. Laboratory tests show normal electrolyte levels and complete blood cell count, while imaging reveals calcification in the left kidney suggestive of a possible kidney stone, with no signs of osteoporosis on a bone density test.	Diagnosis	Kidney stones due to overuse of calcium carbonate
			Disease characteristics	Bone demineralization
			Disease characteristics	Absence of infection
Case 166	Internal Medicine	A 67-year-old woman presents with progressive bilateral lower extremity edema, confusion, orthostatic hypotension, and dry mucus membranes, with physical examination showing +1 pitting edema and occasional bradycardia. Laboratory results reveal normocytic anemia, elevated brain natriuretic peptide, low early morning cortisol, and imaging shows normal sinus rhythm on EKG, negative CT for pulmonary embolism, and intact ejection function on echocardiography.	Diagnosis	Primary adrenal insufficiency
			Diagnosis	No further testing
			Treatment	Hydrocortisone and fludrocortisone
Case 167	Internal Medicine	A 68-year-old man presents with severe pain and swelling in the left first MTP joint, right knee and medial malleolar pain, and painful skin nodules on the lower extremities, with physical examination showing an edematous and tender left first MTP joint and multiple erythematous nodules. Laboratory results reveal a white blood cell count of 12,000 cells/mm³, elevated lipase (>16,000 U/L) and amylase (>4700 U/L) levels, normal antinuclear antibody and immunoglobulin G4 levels, and joint aspirate showing purulent material with a white blood cell count of 6800 cells/mm³ predominantly neutrophilic, while imaging includes CT scans of the ankle	Diagnosis	Pancreatitis, polyarthritis, and panniculitis syndrome
			Disease characteristics	Treatment of the underlying etiology
			Treatment	Typical radiographic findings include multiple osteolytic lesions
Case 168	Major Surgery	A 68-year-old woman presents with nausea, vomiting, diffuse abdominal pain, mild abdominal distension, and tenderness, particularly in the right upper quadrant, with normal vital signs and no guarding or rebound. Laboratory results show normal white blood cell count, hemoglobin, and bilirubin levels, with elevated alanine aminotransferase and aspartate aminotransferase levels, while abdominal MRI findings are not described in the text.	Diagnosis	Metastatic invasive ductal carcinoma
			Treatment	Surgery with adjuvant radiation, hormonal therapy, and systemic chemotherapy
			Treatment	Negative on ink

Case 169	Major Surgery	A 70-year-old man presents with generalized abdominal pain, distention, and deteriorating mental status, accompanied by fever, hypotension, tachycardia, pallor, poor capillary refill, and absent bowel sounds. Laboratory results show leukocytosis, anemia, elevated ALT, bilirubin, CRP, creatinine, and urea, while imaging reveals a large air-filled bowel loop in the left hemithorax, distended bowel loops, eventration of the left hemidiaphragm, and multiple abscesses between bowel loops and within the pelvis.	Diagnosis	Complicated inguinal hernia with the appendix inside the hernial sac (Amyand hernia)
			Disease characteristics	The diagnosis is most often made intraoperatively
			Treatment	Hernioplasty is contraindicated if the appendix is perforated
Case 170	Major Surgery	A 70-year-old woman presents with progressive abdominal pain, mild distention, and two episodes of vomiting, with physical examination revealing fullness and mild tenderness in the epigastric and left upper quadrant regions. Laboratory results show elevated blood glucose, normal metabolic panel, and CBC, while imaging reveals a nonspecific bowel gas pattern on plain radiographs and an abdominal CT scan is performed due to increasing pain.	Diagnosis	Left paraduodenal hernia
			Diagnosis	Adhesive bands
			Examination	Abdominal CT scan
Case 171	Minor	A 71-year-old man presents with nocturia, slow urinary stream, and a sense of incomplete emptying, with a digital rectal examination revealing an asymmetric prostate gland. Laboratory results show a PSA level of 2.4 ng/mL, and imaging via bladder ultrasonography reveals a postvoid residual urine volume of 25 mL with no abnormalities.	Diagnosis	Benign prostatic hypertrophy
			Treatment	Initiate luteinizing hormone-releasing hormone (LHRH) agonist therapy
			Treatment	Any of the above
Case 172	Internal Medicine	A 72-year-old man with a history of COPD, diabetes, hypertension, atrial fibrillation, and prior stroke presents with sudden, constant, diffuse abdominal pain, nausea, bilious emesis, and guaiac-positive stool, with physical examination showing mild diffuse abdominal tenderness. Laboratory results reveal an elevated WBC count with bandemia, while CT of the abdomen and pelvis is performed.	Diagnosis	Pneumatosis intestinalis
			Disease characteristics	Abdominal pain
			Examination	CT
Case 173	Internal Medicine	A 72-year-old woman presents with worsening lower back pain, a history of osteoarthritis, and an unremarkable physical examination except for well-healed surgical scars. Laboratory results show hypercalcemia with a calcium level of 14 mg/dL and elevated creatinine at 1.4 mg/dL, while imaging reveals multiple bony lesions on MRI and CT of the chest, abdomen, and pelvis.	Examination	Perform a biopsy of one of the liver lesions
			Disease characteristics	Complete blood count every year
			Examination	Coronary artery disease
Case 174	Internal Medicine	A 74-year-old immunocompromised woman presented with a 2-month history of unilateral eyelid swelling and redness, with physical examination revealing an erythematous, swollen, tender, and warm left eyelid, while the right eye appeared normal and there were no systemic symptoms. Laboratory tests including HIV and tuberculosis were negative, complete blood count and comprehensive metabolic panel were normal, and an eyelid biopsy with Grocott methenamine silver stain was performed.	Diagnosis	Disseminated histoplasmosis
			Disease characteristics	Choroidal neovascularization
			Disease characteristics	Intracellular, uniform oval bodies seen in macrophages; itraconazole
Case 175	Internal Medicine	A 74-year-old man presents with easy bruising, fatigue, generalized weakness, febrile neutropenia, and respiratory distress, with physical examination revealing diminished breath sounds and rhonchi in the right upper lung field. Laboratory and imaging results show pancytopenia, a dense right upper-lobe consolidation with narrowing of the bronchus, mediastinal lymphadenopathy, and a necrotic endobronchial lesion obstructing the right upper lobe, confirmed by bronchoscopy and biopsy.	Diagnosis	Endobronchial mucormycosis
			Disease characteristics	Diabetes
			Examination	Histologic examination
Case 176	Internal Medicine	A 75-year-old man presents with dyspnea, dry cough, left-sided chest pain, and respiratory distress, showing labored breathing, peripheral cyanosis, and absent breath sounds in the left hemithorax. Laboratory and imaging results reveal a left-sided tension pneumothorax on chest radiograph, with blood gas	Diagnosis	Reexpansion pulmonary edema
			Disease characteristics	After drainage of the pneumothorax, chest radiography shows unilateral florid edema of the reexpanded lung

		analysis showing a pH of 7.30, PCO2 of 35.4 mm Hg, PO2 of 52.8 mm Hg, and bicarbonate of 17.2 mEq/L.	Treatment	Maintaining good diuresis and providing supplemental oxygen and ventilatory support
Case 177	Minor	A 75-year-old woman with fever, rash, skin pain, headache, sore eyes, and cervical and axillary lymphadenopathy presented with erythematous macules that blanched with diascopy and purpuric lesions on the lower legs. Laboratory results showed atypical lymphocytosis, elevated ALT at 1375 U/L, white blood cell count > 12,000 cells/µL, eosinophil count > 900 eosinophils/µL, negative hepatitis serologies, and a biopsy revealed necrotic keratinocytes with a lymphohistiocytic infiltrate and occasional eosinophils.	Diagnosis	Drug eruption
			Diagnosis	Human herpesvirus 6
			Disease characteristics	All of the above
Case 178	Internal Medicine	A 76-year-old woman presents with intense diffuse abdominal pain, nausea, dark brown liquid bowel movements, irregular heart rhythm, slight expiratory wheezing, and mild abdominal tenderness, with vital signs showing tachycardia and low oxygen saturation. Laboratory tests reveal a hemoglobin level of 12.1 g/dL and subtherapeutic INR of 1.4, while imaging shows no free fluid in the abdomen, hyperinflated lung fields, and no acute abdominal abnormalities on CT angiography.	Treatment	Emergency vascular surgery consultation
			Examination	CT angiography
			Disease characteristics	Pain out of proportion to physical exam
Case 179	Internal Medicine	A 78-year-old man presents with a 1×1 cm indurated nodular mass on the left lateral margin of his tongue, accompanied by two areas of ulceration, but no tenderness, bleeding, or drainage. Panoramic radiograph shows minimal bone destruction around a blade implant in the left mandible, and a biopsy specimen has been sent for histopathologic diagnosis.	Diagnosis	Actinomycosis
			Disease characteristics	Antibiotic therapy
			Treatment	All of the above
Case 180	Minor	A 78-year-old man with moderate benign prostatic hypertrophy presented with urinary retention, shaking chills, fever, and fluctuating delirium, with unremarkable physical and neurologic examinations except for altered mental status. Laboratory results showed normal complete blood count and metabolic panel, urinalysis with white blood cells, positive leukocyte esterase and nitrite, and urine culture with Klebsiella, Escherichia coli, and Proteus, leading to treatment with ciprofloxacin, followed by relapse and eventual death from Clostridium difficile pancolitis after treatment with ertapenem.	Diagnosis	Progression of vascular dementia
			Disease characteristics	Renal failure
			Disease characteristics	Bronchoscopy
Case 181	Internal Medicine	A 79-year-old woman with a pacemaker presents with a regular heart rate of 60 beats/min, blood pressure of 135/75 mm Hg, no peripheral edema, and a soft S4 heart sound, with a well-healed pacemaker site. Laboratory and imaging results show normal fasting blood sugar, electrolytes, and creatinine at 118 µmol/L, with an ECG indicating sinus rhythm and intermittent ventricular pacing, and pacemaker interrogation revealing three asymptomatic atrial high-rate episodes over a week, 6 months prior.	Diagnosis	Subclinical atrial fibrillation
			Treatment	Watchful waiting
			Treatment	Monitor and treat with an anticoagulant if atrial fibrillation is detected or if longer, device-detected episodes are documented
Case 182	Minor	A 7-year-old girl presented with a persistent rash on her face and extremities since infancy, characterized by erythematous, crusted plaques with serous discharge and excoriation. Laboratory findings revealed a markedly low serum zinc level of 28.22 µg/dL, and histopathology showed psoriasiform hyperplasia, epidermal pallor, and intraepidermal bullae formation.	Diagnosis	Acrodermatitis enteropathica
			Diagnosis	All of the above
			Treatment	All of the above
Case 183	Paediatrics	A 9-year-old girl presents with several episodes of vomiting, bilateral throbbing headaches, unsteady gait, and horizontal diplopia, with physical examination revealing an unsteady, wide-based gait and abnormal finger-to-nose coordination. Laboratory tests are unremarkable, and MRI of the brain is performed.	Diagnosis	Pontine glioma
			Disease characteristics	Infratentorial
			Disease characteristics	The tumor is impinging on the ophthalmic artery
Case 184	Internal Medicine	A 60-year-old man presents with severe abdominal pain in the epigastrium and right hypochondrium, fever, jaundice, mild bilateral lower limb edema, and dehydration, with tenderness in the abdominal examination and a negative Murphy sign. Laboratory	Diagnosis	Chronic pancreatitis associated with diverticulosis
			Disease characteristics	Osteoporosis

		results show leukocytosis, anemia, elevated liver enzymes, high bilirubin levels, and renal impairment, while imaging reveals dilation of the biliary radicals and common bile duct without gallstones.	Disease characteristics	Abdominal pain
Case 185	Internal Medicine	A 40-year-old man presents with sudden-onset palpitations and dyspnea, a heart rate of 165 beats/min, and a rapid, irregular heart rhythm on examination, but no chest pain or other distressing symptoms. Laboratory tests, including CBC, serum electrolytes, cardiac enzymes, and coagulation panel, are normal, as is the chest radiograph, while ECG shows a rapid heart rate.	Diagnosis	Atrial fibrillation
			Diagnosis	Metoprolol
			Treatment	Infection
Case 186	Psychiatry	A 20-year-old college student presents with moderate to severe acne, regular menstrual periods, and a desire for birth control, appearing hesitant and timid during the examination, with normal vital signs and no abnormalities on physical examination. Laboratory tests show normal electrolyte levels, a normal complete blood cell count, a negative pregnancy test, and negative sexually transmitted disease testing, leading to referrals for psychological counseling and gynecological evaluation.	Diagnosis	Dependent personality disorder
			Examination	Brain imaging can be used in research to help identify structural and functional patterns associated with personality disorders
			Disease characteristics	They are both cluster C disorders that cause disruptions in maintaining healthy relationships
Case 187	Internal Medicine	A 54-year-old man presents with diffuse pruritus, scleral icterus, jaundiced skin, and a dark bronze skin color, with a palpable and mildly tender liver. Laboratory results show elevated total bilirubin, alpha-fetoprotein, aspartate aminotransferase, alanine aminotransferase, and ferritin levels, while imaging reveals moderate ascites, portal gastropathy, cirrhotic liver morphology, and a 10.6-cm hepatic mass, with HFE genetic testing indicating a homozygous C282Y mutation.	Diagnosis	Hepatocellular carcinoma
			Disease characteristics	Cirrhosis
			Examination	MRI
Case 188	Internal Medicine	A 14-year-old boy presents with severe hypertension, headache, sweating, flushing, blurred vision, nausea, dizziness, and mild abdominal tenderness, appearing anxious and diaphoretic. Laboratory results show elevated metanephrine and normetanephrine levels, with an abdominal CT revealing a mass in the right adrenal gland, while the brain CT is unremarkable.	Diagnosis	Pheochromocytoma
			Disease characteristics	VHL syndrome
			Treatment	Alpha-blockade followed by beta-blockade
Case 189	Minor	A 66-year-old woman with a history of type 2 diabetes, stage IV chronic kidney disease, and hypertension presents asymptomatic with a BMI of 35.1 kg/m², blood pressure of 194/65 mm Hg, and sinus bradycardia with second-degree Mobitz type 2 AV block. Laboratory results show elevated potassium at 5.7 mEq/L, BUN at 49 mg/dL, creatinine at 3.9 mg/dL, and eGFR at 13 mL/min/1.73 m², with an unremarkable chest radiograph.	Diagnosis	Bradycardia, renal failure, atrioventricular nodal blockade, shock, and hyperkalemia syndrome
			Treatment	Calcium, beta agonist, insulin, and sodium bicarbonate intravenous solution
			Treatment	Administer digitalis antibodies
Case 190	Minor	A 60-year-old man presents with a 2-week history of gross hematuria, normal vital signs, and bruising on the upper extremities, with no abdominal or flank tenderness. Laboratory results show anemia with a hemoglobin level of 12.0 g/dL, an INR of 3.2, urinalysis with grossly bloody urine and >30 RBCs/HPF, and ultrasound revealing no masses, hydronephrosis, or stones, while cystoscopy findings are pending further evaluation.	Diagnosis	Urothelial cell carcinoma
			Disease characteristics	Smoking
			Examination	Cystoscopy
Case 191	Internal Medicine	An 80-year-old man presents with an 8-year history of a gradually worsening, occasionally pruritic rash covering 30%-40% of his body, characterized by reddish-brown scaling patches and thin plaques with some areas resembling "cigarette paper." A shave biopsy reveals a lymphocytic infiltrate concentrated at the dermoepidermal junction with numerous T lymphocytes within the epidermis, and minimal spongiosis.	Diagnosis	Mycosis fungoides
			Disease characteristics	PUVA therapy
			Treatment	Atypical lymphocytes lined up at the dermoepidermal junction and present in the epidermis
Case 192	Internal Medicine	A 72-year-old man presents with personality changes, confusion, occasional slurred speech, irritability, and difficulty chewing, with physical examination revealing slight swelling and redness inside the left side of his	Diagnosis	Localized mouth or tooth infection
			Disease characteristics	Loss of teeth

		mouth and a swollen left cheek. Laboratory results show a normal complete blood cell count and urinalysis without blood, infection, glucose, or protein.	Disease characteristics	Reduced mobility and neglected hygiene are common consequences of dementia, and can potentially affect oral health
Case 193	Internal Medicine	A 59-year-old man with a history of hypertension, hypokalemia, type 2 diabetes, hyperlipidemia, asthma, sleep apnea, and GERD presents with persistent hypertension and hypokalemia despite medication, with a physical examination showing no acute distress and normal findings. Laboratory results reveal a low potassium level of 3.4 mmol/L, an elevated urine albumin/creatinine ratio of 47 mg/g, high cholesterol and triglyceride levels, and an elevated aldosterone-to-renin ratio of 75.5 with an aldosterone level of 13.9 ng/dL.	Diagnosis	Primary hyperaldosteronism
			Examination	Saline infusion test
			Treatment	Spironolactone
Case 194	Internal Medicine	A 71-year-old man with a history of multiple myeloma presents with severe epigastric pain after eating, accompanied by erythematous plaques and vesicles on his abdomen and back, and a crusted plaque on the right popliteal fossa. Laboratory results show elevated aspartate aminotransferase and alanine aminotransferase levels, with normal bilirubin, alkaline phosphatase, and amylase levels, and imaging reveals no gallstones or liver disease, and a normal pancreas.	Diagnosis	Hepatitis secondary to disseminated herpes zoster
			Examination	CD4+ T cells
			Disease characteristics	VZV DNA quantitative PCR
Case 195	Internal Medicine	An 81-year-old man presents with bilateral nonhealing ulcers on his legs, characterized by multiple red to violaceous nodules draining purulent material, with underlying edema and no palpable lymphadenopathy. Skin biopsy reveals superficial and deep granulation tissue with acute suppurative granulomatous inflammation, and acid-fast bacillus stain highlights multiple organisms, with tissue cultures becoming positive on day 6.	Diagnosis	Atypical mycobacterial infection
			Disease characteristics	Prior kidney transplant
			Treatment	Proper sterilization of equipment
Case 196	Minor	A 53-year-old man with type-2 diabetes and hypertension presents with progressively worsening back and neck pain, stiffness, and a stooped posture, with physical examination revealing loss of lumbar curvature, exaggerated dorsal spine curvature, and restricted spinal movement. Laboratory tests show normal hemoglobin and ESR, slightly elevated fasting blood sugar, and normal renal function, while imaging includes lateral radiographs of the lumbosacral and cervical spine and an anteroposterior pelvis radiograph.	Diagnosis	Diffuse idiopathic skeletal hyperostosis
			Disease characteristics	Flowing ossification overlying the anterolateral aspect of 4 contiguous vertebrae
			Disease characteristics	DISH may be complicated by spinal cord compression, dysphagia, and difficulty performing endoscopic procedures
Case 197	Internal Medicine	An 18-year-old male with a history of occasional marijuana use presents with sudden-onset sharp chest pain radiating to the neck, worsened by inspiration, and associated with shortness of breath, but no fever or cough, and physical examination shows normal heart and lung sounds. Laboratory results reveal a slightly elevated white blood cell count, normal metabolic panel, negative urine toxicology, and normal ECG, while chest X-rays are provided for further evaluation.	Diagnosis	Pneumomediastinum
			Examination	Crunching, crackling, or bubbling sounds that are synchronous with the heartbeat on auscultation
			Disease characteristics	Posteroanterior and lateral views
Case 198	Major Surgery	A 38-year-old woman presents with a 2-year history of a tender, firm, nonmobile abdominal wall mass, predominantly premenstrual pain, menorrhagia, and dysmenorrhea, with physical examination revealing a mass to the left of her umbilicus. Laboratory findings are normal, and MRI shows a 3.2×2.4×1.8 cm mass in the left abdominal rectus muscle, interpreted as abdominal wall fibromatosis, with histology showing tan-yellow, soft, lobular, fibrous adipose tissue.	Diagnosis	Endometriosis
			Examination	MRI
			Disease characteristics	Dysmenorrhea
Case 199	Paediatrics	A neonate presents with dysmorphic facial features including a tall forehead, low hairline, ocular hypertelorism, epicanthal folds, low nasal bridge, low-set ears, micrognathia, short neck, broad hands and feet, and hypotonia. Laboratory and imaging results show a normal chromosomal analysis (46, XY) and no	Diagnosis	Fetal valproate syndrome
			Disease characteristics	Maternal VPA ingestion
			Diagnosis	All of the above

		malformations of the heart or internal organs on ultrasound.		
Case 200	Paediatrics	A 6-day-old full-term male infant presents with a midline, purple-red, slightly raised dermal lesion at the L3-L4 spinal segment, measuring 1.5 cm×2 cm with a central dermal pit, and normal neurological examination findings. Spinal canal ultrasonography at 8 days shows the conus medullaris terminating at L3 with no evidence of a tethered cord or subdermal abnormality, and the lesion enlarges to 6 cm×3 cm by 28 days.	Diagnosis	Infantile hemangioma
			Examination	All of the above
			Disease characteristics	Bronchospasm
Case 201	Major Surgery	An 83-year-old man with a history of atherosclerosis and hypercholesterolemia presents with occasional dull abdominal pain and a pulsating sensation, with physical examination revealing mild wheezing, a systolic murmur, a soft abdominal bruit, and a widened distal abdominal aorta. Laboratory results show elevated cholesterol and triglyceride levels, while imaging confirms a 4.4 cm dilation of the distal abdominal aorta with thrombotic or atherosclerotic buildup, but no aneurysm in the middle abdominal aorta.	Diagnosis	Abdominal aortic aneurysm
			Disease characteristics	Determining the size of aneurysm
			Diagnosis	Pulsating sensation
Case 202	Minor	A 33-year-old nonverbal woman with profound intellectual impairment presents with episodes of eye-blinking, staring, unresponsiveness, rigid posture, ataxic gait, and stereotypic hand movements, alongside a history of scoliosis and coxa valga. Imaging reveals a left-sided coxa valga and posterior spinal fusion from T3 to the sacrum with intact hardware and no significant scoliosis.	Diagnosis	Rett syndrome
			Disease characteristics	All of the above
			Disease characteristics	Profound intellectual disability
Case 203	Internal Medicine	A 59-year-old man presented with sudden intense right flank and lumbar pain, orthopnea, dyspnea on exertion, peripheral edema, and physical examination revealed pale, diaphoretic appearance, mild nonpitting bilateral lower extremity edema, and costovertebral angle tenderness. Laboratory tests showed elevated fasting glucose and leukocyte count, while imaging revealed slightly flattened diaphragmatic domes and large lung volumes without cardiomegaly or effusions, and ECG indicated normal sinus rhythm with poor R-wave progression and left ventricular hypertrophy.	Diagnosis	Pseudoallergic reaction
			Treatment	A 64-year-old woman who presents with bilateral pedal edema, bilateral pleural effusions, and elevated jugular venous distention pressure and whose initial echocardiogram was of poor quality
			Treatment	All of the above
Case 204	Minor	A 61-year-old man presents with fever, dyspnea, productive cough, and worsening arthralgias, along with decreased breath sounds in the right lung base, fine rales, and digital clubbing. Laboratory tests show leukocytosis and elevated ESR and CRP, while imaging reveals right pleural effusion, mediastinal lymphadenopathy, right lower-lobe consolidation, and emphysema, with negative rheumatologic serology.	Diagnosis	Hypertrophic osteoarthropathy
			Disease characteristics	Radionuclide studies
			Examination	Elevated erythrocyte sedimentation rate
Case 205	Minor	A 17-year-old male presents with a 10-day history of a painful, spreading facial rash with multiple vesicular lesions and bilateral submandibular lymph gland enlargement. Laboratory results show a normal white blood cell count, hemoglobin, hematocrit, and platelet count.	Diagnosis	Polymerase chain reaction (PCR)
			Treatment	All of the above
			Disease characteristics	Acyclovir
Case 206	Major Surgery	A 27-year-old woman presents with hot flashes, amenorrhea, fatigue, and a swelling sensation in her neck, with physical examination revealing mild orthostasis and a diffuse, nontender thyroid swelling. Laboratory results show slightly elevated serum potassium and eosinophil count, elevated TSH and anti-TPO antibody levels, and a high FSH level, with normal prolactin, testosterone, and free thyroxin levels, and a negative pregnancy test.	Diagnosis	Autoimmune oophoritis
			Examination	Other autoimmune glandular disorders
			Examination	Estrogen/progesterone withdrawal test
Case 207	Minor	A 77-year-old retired teacher presents with dizziness, imbalance, nausea/vomiting, and a constant diffuse throbbing headache for 2 weeks, with physical examination showing no focal neurological deficits and mild distress. Laboratory studies are unremarkable, MRI reveals mild to moderate hydrocephalus, and CSF analysis shows elevated opening pressure, high protein,	Diagnosis	Leptomeningeal disease
			Treatment	FALSE
			Disease characteristics	IgVH mutated status

		low glucose, and nucleated cells, with headache improvement after lumbar puncture.		
Case 208	Internal Medicine	A 54-year-old woman presents with a 5-day history of worsening diarrhea, weakness, palpitations, and an occasional productive cough, with physical examination revealing dry mucous membranes, tachycardia, and moderate abdominal tenderness. Laboratory results show hyponatremia, hypokalemia, hypochloremia, elevated bicarbonate, and blood urea nitrogen, with a chest radiograph showing no acute abnormalities and a pending CT of the abdomen and pelvis.	Diagnosis	SARS-CoV-2 infection
			Treatment	Start baricitinib, dexamethasone, and remdesivir
			Treatment	14 days
Case 209	Internal Medicine	A 52-year-old woman presents with frequent heartburn, painful swallowing, regurgitation, hoarseness, and mild oropharyngeal redness, alongside a BMI of 33.64. Upper endoscopy reveals LA grade C erythematous inflammation with mucosal breaks in the distal esophagus, normal eosinophil counts, mild chronic gastritis, and no Helicobacter pylori detected.	Diagnosis	Erosive esophagitis
			Treatment	Repeat upper endoscopy in 8-12 weeks to confirm mucosal healing
			Treatment	Proton pump inhibitor (PPI) medication such as omeprazole
Case 210	Minor	A 39-year-old woman presents with a severe occipital headache, vomiting, high blood pressure, and significant nuchal rigidity, but no fever or photophobia, and is in mild distress due to pain. Laboratory analyses are normal, and a noncontrast cerebral CT scan is performed.	Diagnosis	Subarachnoid hemorrhage
			Disease characteristics	Noncontrast brain CT scan
			Examination	Sudden, rapid onset of headache
Case 211	Internal Medicine	A 23-year-old man presents with malaise, fatigue, weight loss, diminished appetite, painful tongue erosions with white exudate, altered mental status, generalized tonic-clonic seizures, dense left hemiparesis, and seventh cranial nerve palsy. Laboratory results show anemia with hemoglobin of 9.4 g/dL, elevated erythrocyte sedimentation rate of 62 mm/hr, and a brain CT scan reveals abnormalities suggestive of increased intracranial pressure.	Diagnosis	Central nervous system (CNS) toxoplasmosis
			Disease characteristics	Primary CNS lymphoma
			Disease characteristics	All of the above
Case 212	Minor	A 29-year-old man presents with a 4-month history of weak urinary stream, urinary straining, and increased frequency, with a severe score on the AUA Symptom Index, but no fever, chills, or dysuria, and physical examination shows a patent urethral meatus and clear urine. Laboratory results indicate urinalysis with 1+ blood, 3-10 RBCs per HPF, and rare bacteria, while imaging via uroflow study shows a start/stop stream pattern and near-complete bladder emptying, with negative tests for sexually transmitted infections and no growth on urine culture.	Diagnosis	Urothelial bladder cancer
			Disease characteristics	Painless gross hematuria
			Disease characteristics	Any carcinoma in situ (CIS)
Case 213	Major Surgery	A 30-year-old woman presents with severe right upper quadrant abdominal pain, bilateral shoulder and wrist pain, fever, chills, and a nearly resolved rash with residual petechial macules and papules, accompanied by a limited range of motion in painful joints. Laboratory tests show a white blood cell count of 9000 cells/µL with elevated neutrophils, normal urinalysis and metabolic panel, and imaging reveals increased perihepatic enhancement concerning for perihepatitis and minimal bilateral tubal scarring, with negative findings for cholecystitis, hepatic inflammation, and pelvic abscesses.	Diagnosis	Fitz-Hugh-Curtis syndrome
			Diagnosis	C trachomatis
			Disease characteristics	Infertility
Case 214	Internal Medicine	A 60-year-old man presents with new-onset pain in both feet, intermittent joint pain in his hands, and diminished sensation to light touch and pinprick in his distal upper and lower extremities, with normal physical examination findings except for hypertension. Laboratory results show normal electrolytes and complete blood cell count, a glycated hemoglobin value of 6.7%, and electromyography and nerve conduction velocity studies indicating peripheral neuropathy in the hands and feet bilaterally.	Diagnosis	Diabetic neuropathy
			Disease characteristics	Pain
			Disease characteristics	Microvascular damage
Case 215	Internal Medicine	A 50-year-old man presents with a 2-day history of fever and persistent left upper quadrant pain following a minor blow, with physical examination showing tenderness and mild edema in the left upper quadrant	Diagnosis	Splenic abscess
			Examination	None of the above
			Disease characteristics	Ultrasonography

		but no rebound or rigidity. Laboratory results reveal an elevated white blood cell count with a left shift, while a CT scan is performed for further evaluation.		
Case 216	Internal Medicine	A 20-year-old man presents with unexplained bruising on his thighs and arms, frequent headaches, and a blood pressure of 150/90 mm Hg, with physical examination showing multiple bruises of varying colors and no other abnormalities. Laboratory and imaging results reveal normal complete blood cell count, electrolyte levels, urinalysis, liver panel, prothrombin time/partial thromboplastin time, lupus anticoagulant test, von Willebrand factor test, clotting factor tests, factor VII and IX tests, and genetic testing for factor V Leiden disease.	Diagnosis	Bruising due to aspirin overuse
			Disease characteristics	Warfarin generally has a higher bleeding risk than aspirin
			Disease characteristics	Underlying psychiatric conditions
Case 217	Psychiatry	A 9-year-old boy presents with suicidal and homicidal behavior, anxiety, hallucinations, and a history of violent actions, with physical examination revealing a slender, poorly nourished appearance, articulation deficit, and flat emotions. Audiograms show mild right conductive hearing loss at certain frequencies, while electroencephalography findings are normal.	Diagnosis	Brief reactive psychosis
			Disease characteristics	A sharp rise is seen in suicide incidence among adolescents and teens as compared with children
			Disease characteristics	Malingering
Case 218	Minor	A 56-year-old woman presents with progressive left hip pain rated as "8 out of 10," which worsens at rest, with a physical examination showing normal vital signs, no acute distress, and unremarkable musculoskeletal findings including normal strength and sensation. Laboratory tests including a complete blood cell count with differential and a comprehensive metabolic panel are within normal limits, and no imaging results are provided.	Diagnosis	Cancer
			Disease characteristics	Positive for CK7 and GATA-3; negative for CK-20
			Treatment	Fulvestrant plus CDK 4/6 inhibitor
Case 219	Major Surgery	A 19-year-old man presents with diffuse right-sided chest pain, shortness of breath, tachycardia, and mildly decreased breath sounds on the right, with no leg edema or fever. Laboratory results show worsening anemia and stable elevated leukocytosis, while imaging reveals an expanding, loculated right pleural effusion with high-density material and layering at the pigtail catheter site.	Diagnosis	Hemothorax
			Treatment	VATS
			Treatment	Transfusion with packed red blood cells
Case 220	Internal Medicine	A 17-year-old male presents with a 2-day history of severe headache, subjective fevers, night sweats, bilious emesis, and splenomegaly, with physical examination showing a temperature of 103.0°F and mild abdominal tenderness. Laboratory results reveal elevated liver enzymes (ALT 110 U/L, AST 159 U/L), elevated bilirubin (total 2.7 mg/dL, direct 0.9 mg/dL), low magnesium (1.2 mg/dL), and thrombocytopenia (platelet count 34×10^3 cells/µL), with a blood smear obtained for further evaluation.	Diagnosis	Malaria
			Diagnosis	To monitor the patient for QTc prolongation
			Treatment	P falciparum
Case 221	Internal Medicine	A 66-year-old man presents with progressive dyspnea, nonproductive cough, finger clubbing, and bibasilar "Velcro crackles," with an oxygen saturation of 96% on room air. Laboratory and imaging results show an elevated ANA titer of 1:160, restrictive defect with impaired gas exchange on pulmonary function testing, and HRCT revealing bilateral peripheral and basal predominant irregular interlobular septal thickening and traction bronchiectasis/bronchiolectasis, leading to a clinical diagnosis of probable usual interstitial pneumonia pattern.	Diagnosis	Idiopathic pulmonary fibrosis
			Treatment	Inhaled treprostinil
			Treatment	Either nintedanib or pirfenidone
Case 222	Internal Medicine	A 57-year-old man presents with falling, bruising, multiple ecchymoses on extremities and trunk, abnormal extraocular movements, and a wide-based unsteady gait. Laboratory results show mild anemia with macrocytosis, elevated AST and ALT with an AST/ALT ratio of 2.1:1, elevated bilirubin, and a CT scan of the head reveals generalized atrophy without acute bleeding.	Diagnosis	Multiple vitamin deficiencies
			Treatment	The diagnosis is made clinically on the basis of the history and physical examination
			Diagnosis	Patients with any sign or symptom consistent with WE and evidence of nutritional compromise
Case 223			Diagnosis	Pseudohypoglycemia

	Internal Medicine	A 28-year-old man presents with episodic heart palpitations, tremors, fatigue, and confusion, with a physical examination showing a well-built, muscular body and unremarkable findings except during an episode where his symptoms resolved after consuming orange juice and crackers. Laboratory results reveal normal blood glucose levels during episodes, normal thyroid and cortisol levels, and a lipid panel showing elevated total cholesterol and LDL, while imaging was not mentioned.	Disease characteristics<br>Disease characteristics	Raynaud phenomenon<br>Relief of the symptoms after eating something
Case 224	Internal Medicine	A 64-year-old woman presents with worsening groin and hip pain, marked tenderness over the ischial tuberosities, greater trochanters, both groins, and symphysis pubis, limited and painful hip range of motion, and later develops shoulder pain and stiffness with limited arm elevation. Initial blood tests show normal ESR and CRP with negative rheumatoid factor, normal hip radiographs, but later tests reveal elevated ESR while rheumatoid factor remains negative.	Diagnosis<br>Disease characteristics<br>Disease characteristics	Polymyalgia rheumatica<br>Osteoarthritis of the hip joint<br>Shoulder and hip girdles
Case 225	Psychiatry	A 14-year-old girl presents with loss of interest in school, trouble sleeping, weight loss, withdrawal from social activities, and tearfulness, with physical examination showing her to be sullen but alert and oriented, without hallucinations or delusions. Laboratory tests, including blood and urine drug screenings and a pregnancy test, were normal, and a psychiatric evaluation revealed no family history of mental illness or signs of abuse, but noted her concerns about disappointing her family and a desire to be alone.	Diagnosis<br>Diagnosis<br>Disease characteristics	Major depressive disorder<br>1 year<br>All of the above
Case 226	Minor	A 68-year-old woman presents with recurrent urinary urgency, frequency, and dysuria, with physical examination revealing a severely atrophic vulva and thin vaginal tissues. Laboratory results show urine microscopy positive for leukocyte esterase, nitrites, and bacteria, with a culture indicating more than 100,000 CFU/mL of pan-sensitive Escherichia coli.	Diagnosis<br>Treatment<br>Treatment	Recurrent UTIs<br>Vaginal estrogen<br>Nitrofurantoin 100 mg daily
Case 227	Internal Medicine	A 60-year-old woman presents with nausea, vomiting, diarrhea, abdominal pain, and worsening substernal chest pain radiating to her back, with physical examination showing mild epigastric tenderness and well-healing discoid lupus lesions. Laboratory results reveal anemia, thrombocytopenia, elevated creatinine, liver enzymes, bilirubin, troponin, and LDH, with schistocytes on peripheral smear, positive hepatitis C, and imaging showing perinephric fat stranding without obstruction.	Diagnosis<br>Disease characteristics<br>Treatment	Lupus-associated thrombotic thrombocytopenic purpura<br>Plasma exchange and high-dose steroids<br>Normocytic anemia
Case 228	Major Surgery	A 65-year-old woman experienced upper abdominal pain, left-shoulder discomfort, dizziness, and abdominal tenderness with distension after a colonoscopy, later developing tachycardia and hypotension. Laboratory results showed a hemoglobin drop from 11.7 g/dL to 6.5 g/dL, and imaging was performed to evaluate her symptoms, leading to a blood transfusion and transfer to a higher-care facility.	Diagnosis<br>Disease characteristics<br>Treatment	Delayed splenic rupture<br>Postpolypectomy syndrome<br>Bowel rest, antibiotics, and outpatient follow-up
Case 229	Internal Medicine	A 66-year-old woman with a history of factor V Leiden thrombophilia presents with DVT after a flight, mild anemia, increased bowel movements, and abdominal bloating, with physical examination showing mild generalized abdominal tenderness and normal bowel sounds. Laboratory results reveal hemoglobin of 8 g/dL, hematocrit of 25%, elevated C-reactive protein at 110 mg/dL, and colonoscopy shows continuous edematous, hyperemic, and friable mucosa in the rectosigmoid.	Diagnosis<br>Treatment<br>Examination	Ulcerative colitis<br>2 years<br>Oral 5-ASA and 5-ASA suppository combination
Case 230	Major Surgery	A 68-year-old woman presents with progressively worsening abdominal pain, decreased appetite, and nausea, with physical examination revealing tenderness in the epigastric region but no guarding or rebound. Imaging shows moderate inflammatory changes around the tail of the pancreas, focal dilation of the distal pancreatic duct, a 6.7-cm pancreatic body mass, and	Diagnosis<br>Treatment<br>Treatment	Breast cancer metastatic to the pancreas<br>Elacestrant<br>HR+/HER2 3+ (IHC) metastatic breast cancer

		hypermetabolic lesions on PET, while biopsy reveals poorly differentiated carcinoma positive for GATA3, CK7, ER, and PR.		
Case 231	Minor	A 30-year-old man presents with intermittent headache, difficulty walking with a tendency to fall on the left side, double vision, short neck, blue sclera, increased skull diameter, and impaired coordination on the left side. Laboratory findings show vitamin D deficiency and raised alkaline phosphatase, while imaging reveals osteoporosis with a T-score of -4.9 SD, osteoporotic fractures, and MRI shows superiorly displaced basiocciput with upward migration of the cervical cord and foramen magnum into the cranial cavity.	Diagnosis	Basilar impression
			Disease characteristics	Neck flexion
			Treatment	Surgery
Case 232	Internal Medicine	A 41-year-old African American man presents with several months of fatigue, worsening shortness of breath, diffuse intermittent abdominal cramping, and an 8-lb weight loss, with physical examination revealing a soft, nontender abdomen and guaiac-positive brown stool. Laboratory results show low hemoglobin (8.4 g/dL), low mean corpuscular volume (69 fL), high platelet count (530×10^3 cells/ L), low iron level (31 µg/dL), low iron saturation (7%), low ferritin (10 ng/mL), elevated aspartate aminotransferase (45 U/L), and alkaline phosphatase (184 U/L), with imaging via ECG showing normal sinus rhythm	Diagnosis	Colorectal cancer
			Disease characteristics	Colonoscopy at age 31 years
			Disease characteristics	Liver
Case 233	Major Surgery	A 70-year-old woman presents with generalized weakness, midline lower abdominal pain, hypotension, tachycardia, abdominal wall bruising, and a firm, tender abdomen with hypoactive bowel sounds. Laboratory results show leukocytosis, anemia, hyperkalemia, renal insufficiency, and anion gap metabolic acidosis, while imaging reveals a large hypodense mass posterior to the left rectus abdominis muscle and a collapsed inferior vena cava.	Diagnosis	Abdominal compartment syndrome
			Disease characteristics	Bladder pressure recordings
			Examination	A 58-year-old man with ascites secondary to liver cirrhosis
Case 234	Internal Medicine	A 30-year-old woman presents with progressive dyspnea, palpitations, bilateral clubbing, peripheral cyanosis, necrotic ulcers, and hepatosplenomegaly, along with a pansystolic murmur and severe tricuspid regurgitation. Laboratory and imaging results show normocytic normochromic anemia, thrombocytopenia, right ventricular enlargement, severe tricuspid regurgitation with vegetation, dilated right pulmonary artery, and chronic thrombosis in both deep venous systems.	Diagnosis	Erythema nodosum
			Disease characteristics	All of the above
			Treatment	Erythema nodosum
Case 235	Minor	A 28-year-old man presents with intermittent lower abdominal pain, loose stools, myalgia, low-grade fever, mild dehydration, and significant tenderness in the left iliac fossa and suprapubic regions with rebound and guarding. Laboratory tests show elevated white blood cell count and C-reactive protein, normal radiographs, CT indicating inflammation deep to the pubic symphysis, and MRI findings, with blood cultures positive for Staphylococcus aureus.	Diagnosis	Osteomyelitis pubis
			Examination	S aureus
			Diagnosis	Radiologically guided needle aspiration and culture of the pubic symphysis
Case 236	Minor	A 26-year-old woman presents with confusion, agitation, sweating, abnormal involuntary eye movement, hypertension, tachycardia, fever, and bilateral hyperreflexia. Laboratory findings show leukocytosis with elevated neutrophils and troponin, while imaging reveals no acute intracranial abnormalities on head CT.	Diagnosis	Serotonin syndrome
			Examination	Dantrolene
			Treatment	Thorough history and physical and neurologic examinations are the basis for diagnosis of serotonin syndrome
Case 237	Internal Medicine	A 63-year-old woman with persistent acid reflux despite PPI use, intermittent epigastric pain, early satiety, and a 15-lb weight loss over 3 months, shows mild epigastric tenderness on examination. Imaging reveals a questionable mass in the ascending colon, enlarged lymph nodes, hypermetabolic osseous lesions, and hypermetabolic activity in the distal stomach, with a colonoscopy confirming a mass near the hepatic flexure.	Diagnosis	Breast cancer with metastasis
			Disease characteristics	A CDK 4/6 inhibitor plus an AI
			Treatment	CDH1
Case 238	Minor		Diagnosis	Acetaminophen overdose

		A 64-year-old man with a history of COPD, alcohol abuse, and depression presented with diffuse jaundice, scleral icterus, and abdominal fluid wave, and was intubated due to obtundation. Laboratory results showed elevated AST and ALT, thrombocytopenia, elevated troponin, and creatinine, with imaging revealing increased hepatic echogenicity, cirrhosis, perihepatic ascites, and lung base opacities, leading to a diagnosis of multisystem organ failure and a poor prognosis.	Disease characteristics	The Rumack-Matthew nomogram can be used for chronic ingestions
			Treatment	All of the above
Case 239	Internal Medicine	A 76-year-old man presents with sudden epigastric abdominal pain, pallor, tachycardia, rapid shallow breathing, and a rigid abdomen with hyperactive bowel sounds, and his stool is guaiac positive. Laboratory results show mild anemia and slight azotemia, while imaging includes unremarkable electrocardiogram findings except for sinus tachycardia and plain abdominal radiographs.	Diagnosis	Pneumoperitoneum from duodenal ulcer perforation
			Examination	Upright chest radiography
			Treatment	Surgical management, broad-spectrum antibiotics, pain control, IV hydration
Case 240	Internal Medicine	A 55-year-old man presents with high-grade fever, diffuse colicky abdominal pain, loose stools with blood, generalized tonic-clonic seizure, confusion, and bilateral basal crackles, with physical examination revealing febrile state, low oxygen saturation, and abdominal tenderness with shifting dullness. Laboratory results show anemia, leukocytosis, thrombocytopenia, prolonged prothrombin time, metabolic acidosis, and positive fibrin degradation products, while imaging reveals multiple brain hemorrhages, hepatosplenomegaly, and ascites, with blood culture indicating growth of long gram-positive bacilli.	Diagnosis	Anthrax
			Examination	Isolation of gram-positive bacilli with a bamboo stick appearance
			Treatment	Intravenous ciprofloxacin plus clindamycin plus antitoxin
Case 241	Internal Medicine	A 27-year-old man presents with hyperemesis, hematemesis, epigastric pain, diarrhea, myalgia, night sweats, rigors, sinus tachycardia, dehydration, and tenderness in the upper chest and neck. Laboratory results show leukocytosis, hypokalemia, slightly elevated creatinine, deranged liver function with elevated gamma-glutamyltransferase, and ECG reveals prolonged QT interval with ST depression, leading to a diagnosis of multiorgan failure due to sepsis.	Diagnosis	Boerhaave syndrome
			Disease characteristics	Vomiting
			Examination	Unilateral pleural effusion, usually left-sided
Case 242	Internal Medicine	A 16-year-old girl presents with sudden severe abdominal pain after a "bear hug," accompanied by a diffuse urticarial rash, tenderness, and a firm mass over the liver edge. Laboratory results show leukocytosis with elevated eosinophils, elevated bilirubin, AST, and ALT levels, while imaging reveals a large hypoechogenic zone in the liver with irregular margins and free fluid around the liver.	Diagnosis	Hepatic hydatid cyst rupture
			Disease characteristics	Albendazole and mebendazole are options for the medical treatment of echinococcosis in patients with contraindications for surgery
			Disease characteristics	Intrabiliary rupture
Case 243	Major Surgery	A 50-year-old man with hypertension, type 2 diabetes, and end-stage renal disease on peritoneal dialysis presents with epigastric pain, dyspnea, fever, tachycardia, and diffuse abdominal tenderness. Laboratory results show elevated white blood cell count, troponin, brain-type natriuretic peptide, sodium, potassium, chloride, blood urea nitrogen, and creatinine, with chest radiography indicating a mild pleural effusion and ECG showing nonspecific T-wave changes.	Diagnosis	Acute peritonitis
			Diagnosis	Dialysate culture positive for fungal infection
			Treatment	Staphylococcus epidermidis
Case 244	Internal Medicine	A 47-year-old man presents with severe, bandlike epigastric pain radiating to the back, worsened by lying flat, accompanied by mild nausea, and physical examination reveals exquisite tenderness in the epigastric and bilateral upper quadrants with rebound tenderness and guarding. Laboratory results are normal, and imaging shows no free air under the diaphragm on chest X-ray, normal abdominal ultrasound, and CT images are provided for further evaluation.	Diagnosis	Perforated peptic ulcer
			Examination	H pylori infection
			Disease characteristics	Endoscopy
Case 245	Internal Medicine	A 45-year-old man presents with a right-sided varicocele and physical examination reveals a temperature of 99.7 °F, blood pressure of 124/74 mm	Diagnosis	Renal cell carcinoma
			Disease characteristics	Thyroid-stimulating hormone measurement

		Hg, and pulse of 84 beats/min. Laboratory results show a hemoglobin level of 9.2 g/dL, MCV of 74 fL, calcium level of 11.1 mg/dL, positive urinalysis for blood, and a CT scan reveals a right renal mass with renal vein thrombosis and scattered pulmonary nodules.	Examination	Tobacco use
Case 246	Internal Medicine	A 49-year-old man with a history of alcohol abuse and pancreatitis presents with 3 days of intermittent periumbilical abdominal pain, nausea, vomiting, and diarrhea, appearing uncomfortable with tenderness in the epigastric and periumbilical regions. Laboratory results show elevated white blood cell count, platelet count, liver enzymes, amylase, and lipase, while CT imaging of the abdomen/pelvis was performed.	Diagnosis	Superior mesenteric vein thrombosis
			Disease characteristics	Bowel rest and fluid resuscitation should be promptly initiated
			Treatment	All of the above
Case 247	Internal Medicine	A 33-year-old man presents with altered mental status, agitation, paranoia, extreme diaphoresis, convulsions, dilated pupils, hypotension, tachycardia, tachypnea, hypoxia, and hyperthermia at 108.1°F. Laboratory and imaging results show a wide-complex tachycardia on cardiac monitoring, later identified as narrow-complex tachycardia on a 3-lead rhythm strip, with a presumptive diagnosis of heatstroke due to cocaine toxicity.	Diagnosis	Hyperkalemia
			Disease characteristics	The patient must have neurologic impairment.
			Disease characteristics	Before treating for heatstroke, the clinician should first rule out all other diagnoses.
Case 248	Paediatrics	An 11-year-old girl presents with painful right facial swelling, recurrent fever, severe leg pain, fatigue, a hard fixed mass in the mandible, pale conjunctivae, and a mild systolic murmur. Laboratory results show anemia with hemoglobin at 8.2 g/dL, elevated liver enzymes, and a maxillofacial CT reveals an enhancing lytic mass in the right mandible, while biopsy shows small round cells positive for S-100, vimentin, and CD99.	Diagnosis	Ewing sarcoma
			Diagnosis	t(11;22) by RT-PCR
			Diagnosis	Acute mastoiditis
Case 249	Internal Medicine	An 18-year-old woman presents with generalized abdominal pain, nausea, increased abdominal girth, low-grade fever, and a large, immobile, tender abdominal mass with marked ascites. Laboratory results show anemia, thrombocytopenia, elevated CA-125, and imaging reveals a complex pelvic mass with ascites, while biopsy and culture confirm Mycobacterium tuberculosis infection.	Diagnosis	Genital tuberculosis with peritonitis
			Treatment	Biopsy of mass
			Examination	Culture with examination on Ziehl-Neelsen staining
Case 250	Internal Medicine	An 18-year-old woman presents with a syncopal episode during exertion, normal vital signs, and an unremarkable physical examination. Laboratory tests, including CBC, chemistry panel, pregnancy test, and toxicology screen, are normal, as is the chest radiograph, while the ECG is shown above.	Diagnosis	Long QT syndrome
			Disease characteristics	Genetic screening
			Diagnosis	All of the above
Case 251	Internal Medicine	An 80-year-old man with a history of COPD experiences exertional shortness of breath and brief episodes of lightheadedness, with physical examination revealing distant breath sounds, frequent skipped heartbeats, and an accentuated second heart sound. Laboratory workup shows a blood pressure of 80/46 mm Hg during an episode of lightheadedness, and ECG indicates atrial fibrillation with rapid ventricular response.	Diagnosis	Nonsustained ventricular tachycardia
			Disease characteristics	Regularity of the RR interval
			Examination	The presence of hemodynamic stability should be regarded as diagnostic of supraventricular tachycardia with aberrancy
Case 252	Internal Medicine	An 82-year-old woman with dementia presents with abdominal pain, massive abdominal distention, dry tongue, reduced skin turgor, and high-pitched bowel sounds, with a history of severe COPD, hypertension, osteoarthritis, and constipation. Laboratory tests show normal white blood cell count, hemoglobin of 13.6%, CRP of 4 mg/L, sodium of 134 mEq/L, potassium of 4.1 mEq/L, urea of 30.1 mEq/L, and creatinine of 1.5 mg/dL, while an abdominal radiograph reveals a greatly dilated loop of sigmoid bowel extending into the upper abdomen with absence of rectal gas.	Diagnosis	Sigmoid volvulus
			Disease characteristics	Sigmoidopexy
			Treatment	Most cases of sigmoid volvulus recur; although immediate surgical treatment is rarely necessary, patients should follow up with a general surgeon promptly after a period of inpatient observation
Case 253	Minor	An 84-year-old woman presents with a painless, intensely itchy blistering rash on her right hand, arm, upper chest, and abdomen, with tense bullae and vesicles on an erythematous base, and no mucous membrane involvement or Nikolsky sign. Laboratory	Diagnosis	Bullous pemphigoid
			Disease characteristics	Steroids
			Treatment	Tense blisters

		tests show mild eosinophilia, normal erythrocyte sedimentation rate, antinuclear antibody titer, and C-reactive protein level, with blood urea nitrogen at 28 mg/dL and creatinine at 2.1 mg/dL.		
Case 254	Major Surgery	An 85-year-old man presents with sudden-onset severe abdominal pain radiating to the left inguinal region, light-headedness, nausea, and tenderness in the left iliac fossa and costovertebral angle, with vital signs showing a heart rate of 105 beats/min and blood pressure of 110/90 mm Hg. Laboratory results reveal unremarkable electrolytes, complete blood cell count, and coagulation panel, but arterial blood gas analysis shows metabolic acidosis, leading to an urgent CT scan and transfer for immediate intervention.	Diagnosis	Ruptured abdominal aortic aneurysm
			Treatment	Endoleak
			Treatment	>5.5 cm
Case 255	Paediatrics	An 8-day-old boy presents with tachypnea, difficulty feeding, and a heart rate of 296 bpm, but appears non-distressed with normal oxygen saturation and clear lung sounds. The ECG shows a rapid heart rate consistent with supraventricular tachycardia.	Diagnosis	Supraventricular tachycardia
			Treatment	Adenosine
			Disease characteristics	Idiopathic
Case 256	Internal Medicine	A 43-year-old man presents with severe intermittent headaches, subjective fever, chills, weakness, abdominal pain, reduced appetite, early satiety, and significant weight loss, with physical examination revealing tachycardia, protuberant abdomen, tenderness in the epigastrium and right upper quadrant, and palpable hepatomegaly. Laboratory results show leukocytosis, anemia, hypoalbuminemia, elevated alkaline phosphatase, and CT imaging of the abdomen reveals multiple low-attenuating liver lesions with enlarged lymph nodes in the gastric and peripancreatic regions.	Diagnosis	Metastatic gastric cancer
			Disease characteristics	Gastric cancer tends to show nonspecific symptoms early on and it develops slowly over many years.
			Disease characteristics	Diet rich in vegetables and fruits
Case 257	Internal Medicine	A 34-year-old woman presents with right ear pain, dizziness, throat discomfort, burning sensation in the left arm and leg, right-sided incoordination, hiccups, right-sided ptosis and miosis, nystagmus, and decreased sensation on the right face and left body. Laboratory tests are normal, a noncontrast CT scan of the head is normal, and MRI of the brain shows a lesion in the right lateral medulla.	Diagnosis	Right-sided lateral medullary stroke (ie, Wallenberg syndrome)
			Examination	Ipsilateral decreased temperature sensation on the face
			Disease characteristics	MRI head scan and MR angiography
Case 258	Internal Medicine	A 43-year-old man presents with dyspnea, dysphagia, muffled voice, and foul-smelling breath, with physical examination revealing erythematous posterior pharynx, poor dentition, and swelling around the left neck and mandibular angle. Laboratory results show mild leukocytosis with neutrophilic predominance, and imaging reveals parapharyngeal and retropharyngeal swelling with airway deviation.	Diagnosis	Parapharyngeal/retropharyngeal cellulitis and abscess formation
			Treatment	Third-generation cephalosporins
			Treatment	A combination of dissociatives-sedatives, anesthetics, and adjunctive agents
Case 259	Internal Medicine	A 37-year-old athletic woman with a history of anxiety disorder presents with chest-pounding palpitations, severe dyspnea, lightheadedness, and chest heaviness, appearing pale, diaphoretic, lethargic, and in mild respiratory distress, with an irregular tachycardic heart rate of 170-300 beats/min and blood pressure of 80/46 mm Hg. Initial ECG shows an irregular, wide complex tachycardia at 224 beats/min, which converts to normal sinus rhythm at 58 beats/min after synchronized cardioversion.	Diagnosis	Wolff-Parkinson-White syndrome with atrial fibrillation
			Treatment	Digoxin
			Treatment	Explain your suspicions based on the baseline ECG and arrange electrophysiology testing and possible radiofrequency ablation with the cardiology service
Case 260	Internal Medicine	A 14-year-old athletic boy with type 1 diabetes and Hashimoto thyroiditis presents with multiple fractures over two years, normal physical examination, and no history of bruising or increased joint flexibility. Laboratory results show low calcium and vitamin D levels, normal PTH, and DEXA scan reveals osteopenia with Z-scores of -0.8 at the femoral neck, -1 at the hip, and -1.8 at the lumbar spine.	Diagnosis	Vitamin D deficiency
			Treatment	Vitamin D at 50,000 IU weekly for 6 weeks
			Examination	Tissue transglutaminase immunoglobulin (Ig)A level with a concurrent IgA level
Case 261	Psychiatry	A 36-year-old man presents with agitation, disheveled appearance, superficial forearm cuts, ecchymosis on the forehead, tachycardia, and labile affect, reporting	Diagnosis	Borderline personality disorder
			Diagnosis	Decreased need for sleep

		feelings of emptiness and a history of self-mutilation. Laboratory and imaging results show normal ECG, head CT, urine toxicology, urinalysis, complete blood count, metabolic profile, and thyroid-stimulating hormone levels.	Treatment	Medications may be useful adjunctively but may not maintain their effectiveness
Case 262	Minor	A 55-year-old man presents with a new-onset lazy eye characterized by profound left-sided ptosis and lateral downward deviation of the left eye, with normal right eye movements and no diplopia. Laboratory tests, including an electrolyte panel and complete blood cell count with differential, are normal, and an ice test for ptosis is negative.	Diagnosis	Brain tumor
			Examination	The diagnosis is based on location, appearance on imaging, and pathology tests
			Examination	There are some differentiating features, but neuroimaging is necessary to confirm the diagnosis
Case 263	Minor	An 8-year-old boy presents with severe right knee pain, swelling, fever, and inability to bear weight, with physical examination showing a warm, mildly effused knee and limited flexion due to pain. Laboratory results reveal elevated white blood cell count, C-reactive protein, and erythrocyte sedimentation rate, while ultrasound shows fluid in the suprapatellar bursa, and MRI is ordered for further evaluation.	Diagnosis	Pyomyositis
			Disease characteristics	History of recent injury to the muscle
			Treatment	Lack of an abscess
Case 264	Minor	A 38-year-old man presents with altered mental status, hypothermia, bradycardia, and respiratory distress, with physical examination revealing rhonchi and dullness in the right lower lung field, cold skin, and delayed capillary refill. Laboratory results show a small elevation in white blood cell count, normal metabolic panel, and chest radiograph indicating right lower lobe consolidation consistent with pneumonia.	Diagnosis	Hypothermia
			Disease characteristics	All of the above
			Disease characteristics	Conduction
Case 265	Paediatrics	A 4-year-old unvaccinated girl with developmental delay presents with wheezing, a temperature of 99.1°F, and expiratory wheezing with occasional crackles on lung examination. Chest X-ray findings are not explicitly described in the text.	Diagnosis	Tracheoesophageal fistula
			Disease characteristics	Chest pain
			Treatment	The patient should undergo emergent endoscopic removal of the battery as soon as possible, regardless of NPO status
Case 266	Internal Medicine	An 80-year-old woman presents with new anemia, increased fatigue, minimal pallor, and chronic bone pain, with a physical examination showing postsurgical and radiation changes but no localized tenderness. Laboratory findings reveal a hemoglobin level of 10 g/dL, a sedimentation rate of 88 mm/hr, and peripheral smears are provided for further evaluation.	Diagnosis	Myelophthisic anemia
			Treatment	Paclitaxel
			Treatment	Denosumab every 4 weeks
Case 267	Internal Medicine	A 63-year-old woman presents with a chronic dry cough, hemoptysis, chest pain, shortness of breath, dyspnea, significant lower back pain, and physical examination reveals enlarged supraclavicular lymph nodes, wheezing, atelectasis, and increased work of breathing. Laboratory and imaging results include a chest radiograph, which was ordered but not detailed in the provided information.	Diagnosis	Lung cancer
			Disease characteristics	Poor diet
			Disease characteristics	Squamous cell carcinoma
Case 268	Psychiatry	A 24-year-old woman with a history of schizoaffective disorder presents with severe anxiety, depression, low energy, lack of motivation, and psychomotor agitation, with a mental status examination revealing anxious mood, circumstantial to tangential thoughts, and focusing deficits. Laboratory tests including complete blood count, metabolic panel, thyroid function, vitamin D, folate, and thiamine levels are normal, and her recent physical examination shows no significant findings.	Diagnosis	Bipolar disorder type I, most recent episode mixed, severe
			Disease characteristics	A past diagnosis of schizoaffective disorder
			Disease characteristics	40%
Case 269	Minor	A 52-year-old woman presents with persistent aching pain in the right forearm and palm, painful numbness and tingling in the thumb and fingers, and reduced sensation on the palmar aspect of the thumb, index, and middle fingers, with tenderness and mild paresthesia upon tapping over the medial forearm. Radiographs of the elbow, forearm, and wrist are normal, and routine blood tests show no abnormalities.	Diagnosis	Pronator syndrome
			Disease characteristics	Carpal tunnel syndrome
			Examination	Tenderness over the medial proximal forearm

Case 270	Internal Medicine	An 84-year-old woman presents with asymptomatic hyperkeratotic patches on her legs and a red nodule with central ulceration on her right shoulder, with a medical history of hypertension, polycythemia vera, and gastritis. Laboratory tests show no abnormalities, cultures reveal coagulase-negative Staphylococcus and Pseudomonas aeruginosa, skin biopsy indicates neoplastic cells positive for CD3+, CD8+, CD30+, and imaging shows subcentimetric lymph nodes and increased pathologic metabolism in specific areas.	Diagnosis	Primary cutaneous aggressive epidermotropic CD8+ cytotoxic T-cell lymphoma
			Disease characteristics	Lung and central nervous system
			Treatment	Gemcitabine
Case 271	Internal Medicine	A 51-year-old man with well-controlled HIV presents with rectal pain, hematochezia, tenesmus, and weight loss, with physical examination revealing mild tenderness, an anal fissure, and external hemorrhoids. Laboratory results show mild anemia and undetectable HIV viral load, while imaging and colonoscopy reveal irregular rectal thickening, lymphadenopathy, and a fungating mass, with biopsy indicating ulcerated mucosa and viral nuclear inclusions.	Diagnosis	Herpes simplex virus infection
			Diagnosis	HHV-6
			Examination	Herpes antibodies
Case 272	Internal Medicine	A 35-year-old man with adult-onset asthma presents with nasal obstruction, smelling loss, postnasal drainage, cough, rhinorrhea, facial pressure, and large nasal polyps, with mild expiratory wheezing on lung auscultation. Laboratory and imaging results show no allergy skin reactions, an FEV1 of 75%, normal chest radiography, and a CT scan revealing severe pansinusitis with complete to near-complete opacification of the paranasal and nasal cavities, along with mild osteitis.	Diagnosis	Nasal polyposis related to aspirin-exacerbated respiratory disease
			Disease characteristics	Type 2 inflammation
			Disease characteristics	Nasal obstruction and anosmia are associated with nasal polyposis
Case 273	Minor	A 23-year-old man presents with bilateral progressive hearing loss, tinnitus, vertigo, headache, blurred vision, ataxia, subcutaneous swelling on the face and back, bilateral facial palsy, and papilledema. Laboratory tests are normal, while MRI reveals bilateral cerebellopontine angle masses with obstructive hydrocephalus.	Diagnosis	Neurofibromatosis type 2 (NF2)
			Disease characteristics	Cranial nerve VIII
			Disease characteristics	Bilateral vestibular schwannomas
Case 274	Minor	A 74-year-old man with a history of myasthenia gravis and chronic corticosteroid use presents with severe left hip pain, bilateral knee pain, left groin pain, decreased range of motion in the left hip, and crepitus in both knees, with a well-healed scar from recent hip replacement. Laboratory tests show normal free testosterone, vitamin D, creatinine, calcium, magnesium, albumin, thyroid-stimulating hormone, and alkaline phosphatase levels, with no monoclonal spike on serum protein electrophoresis, while imaging reveals severe arthritic changes, a shattered left femoral head, and a right proximal femur fracture.	Disease characteristics	Bisphosphonate use
			Treatment	Prior radiation exposure
			Treatment	Teriparatide
Case 275	Internal Medicine	A 62-year-old man presents with sudden-onset palpitations, dizziness, blackouts, bilateral pitting pedal edema, periorbital edema, macroglossia, and ecchymoses, with physical examination revealing inspiratory crepitations and a distended abdomen. Laboratory results show normocytic anemia, elevated serum creatinine, low serum protein and albumin levels, high NT-proBNP and troponin I levels, proteinuria with a 24-hour urinary protein loss of 7.1 g, and imaging reveals marked mesenteric edema and bilateral bulky kidneys with loss of corticomedullary differentiation.	Diagnosis	Systemic amyloidosis
			Disease characteristics	All patients with AL amyloidosis who are not eligible for stem cell transplant should receive combination therapy
			Treatment	Cardiac involvement is the most common cause of mortality in amyloidosis
Case 276	Psychiatry	A 45-year-old woman presents with difficulty concentrating described as "brain fog," daytime anxiety, and increased fatigue, with a physical examination showing she is alert, oriented, and in no apparent distress, with normal vital signs and no abnormalities noted in skin, thyroid, respiratory, cardiovascular, abdominal, or neurological examinations. Laboratory tests reveal normal electrolyte levels, complete blood cell count, iron, vitamin B12, and thyroid function, with lipid levels showing total cholesterol at 160 mg/dL,	Diagnosis	Sleep deprivation due to caffeine overuse
			Disease characteristics	Improved cardiovascular profile
			Disease characteristics	Insomnia and anxiety

		LDL at 110 mg/dL, triglycerides at 170 mg/dL, and HDL at 40 mg/dL.		
Case 277	Internal Medicine	A 70-year-old man with a history of GERD, type 2 diabetes, hypertension, and hyperlipidemia presents with significant abdominal bloating, crampy discomfort, and diarrhea, with a mildly distended but non-tender abdomen on examination. Laboratory results show elevated A1c, low HDL, high LDL, macrocytic anemia, low vitamin B12, and imaging reveals mild fatty liver, while endoscopy and colonoscopy show mild chronic gastritis and normal colonic and duodenal histology.	Diagnosis	Small intestinal bacterial overgrowth
			Examination	Carbohydrate breath test
			Treatment	Oral rifaximin 550 mg three times daily
Case 278	Minor	A 70-year-old man presents with progressive lower-extremity pain, muscle weakness, urinary incontinence, severe leg pain, muscle atrophy, and purplish skin discoloration, with absent ankle movements and reflexes, and absent proprioception, vibratory, and temperature sensation below the hip. Laboratory tests are normal except for elevated ESR and CRP, CSF analysis shows mild protein elevation, and imaging reveals contrast enhancement in the cauda equina nerve roots and lumbar plexus with muscular edema, while electrophysiologic studies show near-absent sensory and motor responses in the lower extremities.	Diagnosis	Lumbosacral radiculoplexus neuropathy
			Disease characteristics	Multifocal motor neuropathy
			Treatment	Multifocal motor neuropathy
Case 279	Internal Medicine	A 17-year-old boy presents with mild anterior chest-wall pain and mild ecchymosis after a collision, with normal physical examination findings except for initial low oxygen saturation corrected with supplemental oxygen. Laboratory results show a mildly elevated white blood cell count, normal troponin I level, and a negative urine drug screen, while imaging reveals sinus tachycardia with mild right-axis deviation on ECG and mild pulmonary and periportal edema on chest CT.	Diagnosis	Commotio cordis
			Treatment	Use of an automated external defibrillator (AED) within 3 minutes of impact
			Examination	A small elevation in his cardiac enzymes but otherwise normal findings
Case 280	Internal Medicine	A 53-year-old woman with a history of hypertension, hyperlipidemia, tobacco use, and obesity presents with a 1-week history of chest pain, dyspnea, weakness, dizziness, and syncope, with physical examination showing elevated blood pressure, jugular venous distention, and obesity. Laboratory results reveal elevated liver enzymes and normal cardiac biomarkers, while imaging shows a dilated aortic arch, and a bedside echocardiogram indicates mild left ventricular hypertrophy with preserved ejection fraction; she later suffers cardiac arrest with ventricular fibrillation, is resuscitated, and admitted for further evaluation.	Diagnosis	Prinzmetal angina or variant angina
			Treatment	ICD
			Treatment	Nonselective beta-blockers
Case 281	Internal Medicine	A 60-year-old man with a history of diabetes, hypertension, and recent respiratory infection presents with worsening retrosternal chest pain radiating to the right shoulder and neck, relieved by bending forward, and physical examination reveals normal heart sounds without murmurs or rubs. Laboratory results show a slightly elevated creatine kinase level, normal troponin I, and ECG findings are provided for further evaluation, with serial cardiac enzyme measurements remaining negative.	Diagnosis	Acute pericarditis
			Examination	PR elevation in lead aVR
			Disease characteristics	Viral/idiopathic etiology
Case 282	Internal Medicine	A 35-year-old man was found in cardiac arrest with a GCS score of 6, symmetrical miosis, and track marks on his forearms, later becoming agitated and demanding discharge after naloxone administration. Laboratory and imaging results include a normal ECG and bradypnea at 6 breaths per minute, with no other abnormalities noted.	Diagnosis	Opioid poisoning
Case 283	Internal Medicine	A 49-year-old Nigerian man with untreated HIV, hypertension, and type 2 diabetes mellitus presents with a blood pressure of 169/103 mm Hg and no rashes, skin	Examination	The patient may have HIV-2 infection and confirmation is required

		lesions, or lymphadenopathy on physical examination. Laboratory results reveal a positive HIV-1/2 antigen/antibody combination immunoassay, a CD4 cell count of 752 cells/mm³, and an undetectable HIV-1 RNA by RT-PCR.	Disease characteristics	HIV-2 is susceptible to NNRTIs
			Disease characteristics	HIV-2 progresses more slowly than with HIV-1
Case 284	Internal Medicine	A 37-year-old man presents with substernal chest pain, painless yellowish papules on his elbows and eyelid, and a milky appearance of the retinal arteries. Laboratory results show a hemoglobin A1c of 9.8%, elevated total cholesterol at 417 mg/dL, triglycerides at 5077 mg/dL, elevated troponin T at 421 ng/L, and an ECG with biphasic T-wave inversions, with cardiac catheterization revealing a 95% occluded mid-left anterior descending artery.	Diagnosis	Lipoprotein lipase deficiency
			Examination	A review of the medication list and the social history
			Examination	A fractionated lipoprotein panel
Case 285	Internal Medicine	A 33-year-old man presents with chest pain, shortness of breath, tachycardia, fever, and low oxygen saturation, with physical examination showing clear lungs and normal heart sounds. Laboratory results reveal slight leukocytosis, normal metabolic panel, troponin, and coagulation profile, while imaging shows small bilateral pleural effusions, lower lobe atelectasis, and CT pulmonary angiography is performed.	Diagnosis	Pulmonary embolism
			Examination	CT pulmonary angiography
			Treatment	Inferior vena cava filter
Case 286	Internal Medicine	A 50-year-old man with a history of stage IIIB cecal adenocarcinoma presents with pressure-like, burning chest pain radiating to the left jaw and arm, improving with rest, and exhibits normal vital signs and unremarkable physical examination findings. Laboratory results show normal complete blood count, creatinine at 1.3 mg/dL, high-sensitivity troponin rising from 8 to 13 ng/L, NT-proBNP at 64 pg/mL, elevated D-dimer at 1.32 µg/mL, with imaging revealing no acute abnormalities on chest X-ray, negative CT for pulmonary embolism, and coronary angiogram showing nonobstructive coronary artery disease with mild luminal irregularities	Diagnosis	Chemotherapy-induced coronary vasospasm
			Treatment	Obtain a 12-lead ECG
			Examination	Calcium channel blocker
Case 287	Internal Medicine	A 68-year-old woman with diabetes presents with substernal chest pressure, progressive dyspnea at rest, tachypnea, accessory muscle use, distended neck veins, irregular heart rhythm, and faint bilateral lung crackles. Laboratory results are pending, and the initial ECG shows irregularities; oxygen saturation is 82% on room air.	Diagnosis	Mobitz type II atrioventricular block
			Treatment	Mobitz type I (Wenckebach) atrioventricular block; no pharmacotherapy and outpatient follow-up
			Diagnosis	Mobitz II with 6:5 conduction block
Case 288	Internal Medicine	A 13-year-old boy presents with epigastric pain, vomiting, malaise, polyuria, weight loss, mild distress, dry oropharynx, epigastric tenderness, and a nodular eczematous lesion on the gluteal region. Laboratory results show elevated blood urea nitrogen, serum creatinine, and calcium levels, low phosphorus and parathyroid hormone levels, marked hypercalciuria, and renal biopsy reveals tubulointerstitial nephritis with tubular calcium deposits.	Diagnosis	Sarcoidosis
			Disease characteristics	Endogenous synthesis of 1,25-dihydroxyvitamin D
			Treatment	Corticosteroids
Case 289	Internal Medicine	A 10-year-old boy presents with chronic splenomegaly, left-sided abdominal pain, and is small for his age, with physical examination revealing a palpable spleen 10 cm below the left costal margin and mild cervical lymphadenopathy. Laboratory results show anemia, thrombocytopenia, elevated eosinophils, and normal liver function tests, while imaging reveals a 15 cm spleen with tortuous and dilated splenic vasculature.	Diagnosis	Chronic malaria
			Examination	Atovaquone-proguanil
			Treatment	IgM titer >2 standard deviations above the mean
Case 290	Psychiatry	A 22-year-old college athlete presents with mood fluctuations, alternating between euphoric and depressive phases, with normal physical examination except for tenderness along the bilateral medial tibial borders. Laboratory tests including urine toxicology, complete blood cell count, metabolic panel, and thyroid panel are normal, and imaging shows a hip stress fracture.	Diagnosis	Cyclothymic disorder
			Examination	Presence of frequent episodes of hypomanic symptoms and depressive symptoms for the past 3.5 years
			Treatment	Aripiprazole
Case 291	Minor		Diagnosis	Narcolepsy

		A 27-year-old woman presents with constant, mild headaches associated with sleepiness and reports being too tired for leisure activities, with a normal physical examination and no mood abnormalities. Laboratory tests, including a comprehensive metabolic panel and thyroid function tests, are normal, brain MRI is normal, and sleep studies reveal a short sleep latency with rapid onset of REM sleep during naps.	Disease characteristics	Thyroid disease
			Disease characteristics	Narcolepsy may be associated with lower maximal oxygen uptake
Case 292	Internal Medicine	A 60-year-old woman with a history of asthma and cough-induced rib fractures presented with minimal tenderness of the mid-thoracic spine and a reduction in height by 1 inch, with an otherwise normal physical examination. Laboratory results showed normal erythrocyte sedimentation rate, hemoglobin, albumin, and calcium levels, while chest radiography was performed and bone density testing was pending.	Diagnosis	Osteoporosis
			Disease characteristics	Omeprazole use
			Examination	Compression fractures are recognizable on a lateral chest x-ray and usually identify the presence of osteoporosis
Case 293	Internal Medicine	A 77-year-old man presents with severe generalized abdominal pain, bilious vomiting, dehydration, prolonged capillary refill, cool extremities, and boardlike abdominal rigidity. Laboratory results show elevated white blood cell count, high C-reactive protein, hypernatremia, hypokalemia, elevated urea and creatinine, acidosis with low bicarbonate, and high lactate, while imaging includes erect chest and supine abdominal radiographs.	Diagnosis	Cholelithiasis
			Examination	Surgical relief of the obstruction, with gallstone removal and intestinal repair
			Treatment	CT scanning
Case 294	Paediatrics	A 28-month-old girl presents with irritability, decreased speech, clumsiness, unsteady ataxic gait, and intermittent jerky eye movements, with physical examination revealing intermittent jerking movements of her arms and legs and a low-pitched systolic murmur. Laboratory tests show a hemoglobin level of 11.2 g/dL with normal white blood cell and platelet counts, normal serum electrolytes, creatinine, liver enzymes, and urine VMA levels, while imaging including abdominal ultrasound, brain CT, and brain MRI show no abnormalities, and lumbar puncture results are normal.	Diagnosis	Opsoclonus-myoclonus ataxia syndrome
			Diagnosis	A combination of cyclophosphamide, a corticosteroid, and IVIG shows significant benefit
			Treatment	Children older than 4 years are rarely affected
Case 295	Internal Medicine	A 45-year-old man with a history of bipolar disorder presents with confusion, incontinence, slurred speech, lateral gaze nystagmus, resting tremor, truncal ataxia, hyperreflexia, and bilateral ankle clonus, along with vital signs showing hypotension, tachycardia, and fever. Laboratory results indicate leukocytosis, hyponatremia, low bicarbonate, elevated creatinine, high BUN, hyperglycemia, elevated lactate, and a high lithium level, with a normal head CT and sinus tachycardia on ECG, while urine tests show low specific gravity and ketones.	Diagnosis	Lithium-induced nephrogenic diabetes insipidus
			Treatment	Strict intake and output documentation
			Disease characteristics	Cerebellar dysfunction, extrapyramidal symptoms, brainstem dysfunction, and dementia
Case 296	Minor	A 52-year-old woman with a history of right-sided inflammatory breast cancer presents with a 5-day history of headaches, lethargy, confusion, and blurred vision, showing a Glasgow coma scale score of 8, with blurred optic disks bilaterally. Laboratory tests reveal a normal complete blood count and metabolic panel, positive urinalysis for leukocytes, nitrates, and bacteria, and a CT scan of the head is performed.	Diagnosis	Obstructive hydrocephalus
			Diagnosis	The sylvian and interhemispheric fissures are widened.
			Treatment	Ventriculoperitoneal shunt
Case 297	Minor	A 43-year-old man presents with severe itching and a rash in both axillae, characterized by erythematous papules forming poorly demarcated plaques in friction areas, with mild conjunctival injection and pale nasal mucosa. Laboratory and imaging results are not provided in the case details.	Diagnosis	Allergic contact dermatitis
			Examination	Patient education
			Treatment	Potassium hydroxide (KOH) preparation
Case 298	Minor	An 80-year-old man presents with diffuse, symmetric yellow-orange plaques on his upper torso and arms, mild fatigue, and mild anemia and thrombocytopenia on examination. Laboratory results show normal cholesterol and triglyceride levels, but serum electrophoresis reveals an IgG kappa monoclonal gammopathy, leading to a diagnosis of smoldering multiple myeloma.	Diagnosis	Normolipemic plane xanthoma
			Disease characteristics	Serum protein electrophoresis and immunofixation
			Examination	Hepatic cholestasis

Case 299	Minor	A 45-year-old woman with a history of hypertension, type 1 diabetes, and Crohn's disease presents with a 2-cm slightly raised lesion with a hyperpigmented border and eschar on her right leg and a moist, ulcerated, weeping wound on her left leg. Laboratory studies show normal blood counts and metabolic panel, with slightly elevated ESR and CRP, and negative skin cultures.	Diagnosis	Pyoderma gangrenosum
			Examination	Topical corticosteroids
			Treatment	Biopsy now
Case 300	Internal Medicine	A 55-year-old woman with COPD, type 2 diabetes, and hypertension presents with fatigue, low-grade fever, tachycardia, dry mucous membranes, and diffuse abdominal tenderness with hyperactive bowel sounds, following a recent history of RSV-associated bronchitis and pneumonia treated with antibiotics. Laboratory results reveal leukocytosis with neutrophilia, mild hypokalemia, elevated blood urea nitrogen, low albumin, and stool testing for gastrointestinal pathogens and Clostridioides difficile toxin is pending.	Diagnosis	Clostridioides difficile colitis
			Treatment	Intravenous bezlotoxumab 10 mL/kg administered once during the course of antibiotic therapy
			Treatment	Oral fidaxomicin
Case 301	Internal Medicine	A 50-year-old man with a history of acute coronary syndrome presents with chronic diarrhea, mild colonic tenderness, and a mild fading rash, with vital signs showing blood pressure of 130/90 mm Hg and pulse of 80 beats/min. Laboratory results reveal an elevated erythrocyte sedimentation rate of 50 mm/hr and C-reactive protein of 4 mg/dL, with normal fecal calprotectin and stool analysis showing 4 pus cells per high power field.	Diagnosis	Drug-induced colitis
			Disease characteristics	PPIs
			Treatment	Dyspepsia
Case 302	Minor	A 38-year-old man presents with a severe right-sided temporal headache, diplopia, right-sided hemiparesis, and numbness, with examination revealing weakness of the left superior and inferior oblique muscles and right hemiparesis. Laboratory tests are normal, cerebrospinal fluid analysis shows no abnormalities, and imaging results are pending further interpretation.	Diagnosis	Hemiplegic migraine
			Disease characteristics	Hemiplegic migraine is typically associated with multiple forms of aura
			Treatment	Prophylaxis should be strongly considered in patients who present with hemiplegic migraine, regardless of headache frequency
Case 303	Minor	A 25-year-old man presents with gradual-onset horizontal diplopia, left lateral gaze nystagmus, left-sided relative afferent pupillary defect, and left optic disc pallor, with no ataxia or meningeal irritation. Laboratory tests are unremarkable except for positive CSF oligoclonal bands, and MRI shows T2 FLAIR high-signal areas in the pons and scattered white-matter hyperintensities, with delayed P100 on visual evoked potential.	Diagnosis	Unilateral internuclear ophthalmoplegia
			Disease characteristics	The medial longitudinal fasciculus (MLF)
			Disease characteristics	MS
Case 304	Minor	A 16-month-old girl presents with a 10-day history of dry cough, facial swelling, irritability, decreased appetite, periorbital swelling, tachypnea, diminished breath sounds, pink discoloration of hands and feet, bilateral pitting edema, and globally decreased deep tendon reflexes. Laboratory results show 2+ proteinuria with normal complete blood count and metabolic panel, while chest imaging reveals diffuse ground glass opacities without focal consolidation or effusion.	Diagnosis	Mercury poisoning
			Disease characteristics	Ingestion of mercury salt
			Treatment	Urine mercury level of 50 µg/L
Case 305	Internal Medicine	A 54-year-old woman presents with discolored toes, nonhealing ulcers, and small nontender nodules on the right foot, with physical examination revealing livedo reticularis and erythema of the feet. Laboratory tests show elevated C-reactive protein, while a biopsy of a nodule reveals fibrinoid necrosis of the vessel wall with mixed infiltrate and disruption of the internal elastic lamina, consistent with livedoid vasculopathy.	Diagnosis	Polyarteritis nodosa
			Disease characteristics	Fever and weight loss
			Examination	Fibrinoid necrosis of the vessel wall of a medium-sized artery, associated with a mixed infiltrate of neutrophils, lymphocytes, and histiocytes
Case 306	Minor	A 50-year-old woman with a history of migraines presents with episodic neurologic events including scotomas, vertigo, nausea, arm incoordination, brain fog, and slurred speech, with physical examination showing optic nerve obscuration, purplish skin discoloration, reduced sensation below the ankles, absent ankle reflexes, and minimal intention tremor.	Diagnosis	Demyelinating neuropathy associated with MAG antibodies
			Disease characteristics	Distal acquired demyelinating neuropathy and anti-MAG related neuropathy

		Laboratory tests reveal elevated IgM kappa and MAG antibody titers, normal lumbar puncture, normal brain and spine MRI, signal changes in the right proximal orbital optic nerve, and tilt table testing confirms autonomic insufficiency, while EMG shows prolonged sensory and motor latencies.	Disease characteristics	Shingles
Case 307	Minor	A 14-year-old girl presents with left ear pain, purulent bloody discharge, facial nerve palsy, and develops severe left orbital pain, double vision, and inability to abduct the left eye. Laboratory tests are normal, CT shows left-sided mastoiditis, CSF analysis is unremarkable, and MRI is performed to rule out venous sinus thrombosis.	Diagnosis	Gradenigo syndrome
			Disease characteristics	MRI
			Examination	Facial nerve palsy
Case 308	Minor	A 67-year-old man with Parkinson's disease presents with recent unintentional weight loss of 30 lb over 12 months, a BMI of 18 kg/m², dry flaky skin, normal heart and respiratory sounds, and increased muscle tone with a shuffling gait. Laboratory and imaging results are not provided in the case details.	Examination	Abdominal CT
			Disease characteristics	All of the above
			Treatment	Refer him to a dietitian
Case 309	Internal Medicine	A 42-year-old man presents with dull anterior precordial and retrosternal chest pain, increased during inspiration and movement, with a blood pressure of 160/102 mm Hg, a regular pulse of 103 beats/min, and an early diastolic murmur in the aortic region. ECG shows no evidence of myocardial injury, and serial cardiac isoenzyme tests are negative for myocardial damage.	Diagnosis	Proximal aortic dissection
			Treatment	Transesophageal echocardiography
			Examination	Heparin
Case 310	Internal Medicine	A 35-year-old woman presents with 2 months of gnawing epigastric pain referred to the back, severe postprandial dyspepsia, abdominal distention, and unintentional weight loss, with physical examination showing moderate epigastric pain and unilateral shifting dullness. Laboratory results reveal anemia with a hemoglobin level of 9 g/dL, elevated erythrocyte sedimentation rate of 120 mm/h, and elevated CA 19-9 level of 50 U/mL, while endoscopy shows circumferential luminal hypertrophy in the stomach with mild hyperemia and no gastric ulceration.	Diagnosis	Linitis plastica
			Disease characteristics	TS-1
			Treatment	Ascitic fluid
Case 311	Internal Medicine	A 55-year-old woman with hypothyroidism presents with progressively worsening dysphagia and odynophagia, with a sensation of food being stuck in her chest, but no nausea, vomiting, or weight loss, and a normal physical examination. Laboratory results are normal, CT shows mild distal esophageal wall thickening, barium esophagram reveals a small caliber esophagus, and EGD shows friable, inflamed mucosa with ulcerations and stenosis in the esophagus, leading to esophageal dilation for relief.	Diagnosis	Esophageal lichen planus
			Examination	Endoscopic dilation plus initiation of topical steroids
			Treatment	Endoscopy
Case 312	Internal Medicine	A 73-year-old woman presents with dysphagia, unintentional weight loss, fatigue, and weakness, with physical examination showing a thin, pale appearance but otherwise normal findings. Laboratory results indicate normocytic anemia with a hematocrit of 26% and hemoglobin of 9.5 g/dL, while imaging via a barium swallow study reveals an irregular area with narrowing of the distal esophagus.	Diagnosis	Esophageal cancer
			Examination	EGD with tissue samples of the lesion
			Treatment	Neoadjuvant chemoradiation therapy with esophageal resection
Case 313	Internal Medicine	A 23-year-old man with eczema, GERD, and seasonal allergies presents with intermittent dysphagia for solid foods, chest pain, and vomiting, and physical examination reveals an erythematous, vesicular rash on his hands and arms. Laboratory results show an elevated eosinophil count of 1200 cells/μL, and upper endoscopy reveals esophageal rings and longitudinal furrows with biopsies showing up to 45 eosinophils per high-power field, indicating eosinophilic esophagitis.	Diagnosis	Eosinophilic esophagitis
			Treatment	Repeat upper endoscopy in 8-12 weeks to reassess esophageal eosinophilia
			Treatment	Proton pump inhibitor
Case 314	Internal Medicine	A 60-year-old woman with uncontrolled diabetes, hypertension, and COPD presents with worsening dyspnea, fever, chest pain, hemoptysis, and tachycardia, requiring intubation and mechanical ventilation, with	Diagnosis	Influenza pneumonia with secondary bacterial pneumonia, complicated by myopericarditis

		physical examination showing hypoxia, supraventricular tachycardia, and rhonchorous breath sounds. Laboratory results reveal elevated creatinine, AST, WBC, lactate, and glucose levels, with a CT scan showing right lower lobe consolidation and ground-glass opacities, ECG indicating atrial fibrillation and ST elevation, and echocardiogram showing reduced ejection fraction and pericardial effusion.	Examination	A 35-year-old with diabetes mellitus
			Disease characteristics	Positive endomyocardial biopsy
Case 315	Internal Medicine	A 46-year-old man presents with progressively worsening breathlessness on exertion, night sweats, intermittent low-grade fever, and multiple areas of hyperpigmentation, with unremarkable cardiovascular and respiratory examinations. Laboratory results show elevated white blood cell count, platelet count, C-reactive protein, lactate dehydrogenase, erythrocyte sedimentation rate, and D-dimer, while imaging reveals a significant pericardial effusion, a multilobular substernal mass in the anterior superior mediastinum, and enlarged mediastinal lymph nodes.	Diagnosis	Thymoma
			Treatment	Myasthenia gravis
			Disease characteristics	Surgical excision
Case 316	Internal Medicine	A 63-year-old man with a history of repaired tetralogy of Fallot presents with exertional dyspnea, a grade 2/6 systolic ejection murmur, a grade 2/4 diastolic murmur, and trace pedal edema, with a blood pressure of 90/57 mm Hg and elevated jugular venous pressure. Laboratory results show elevated NT-proBNP at 1480 pg/mL, ECG with ventricularly paced rhythm and atrial fibrillation, echocardiography indicating decreased LV ejection fraction (25%-30%) with global hypokinesis, and catheterization revealing normal cardiac output and pulmonary pressure of 44/12 mm Hg.	Diagnosis	Cardiomyopathy due to coronary artery disease and sequelae of repaired tetralogy of Fallot
			Disease characteristics	Prolonged QRS duration
			Disease characteristics	As many as 30% of these patients develop LV dysfunction
Case 317	Internal Medicine	An 83-year-old man presents with moderate dyspnea, headache, mild respiratory distress, dry mucous membranes, jugular venous distension, bilateral crackles, 2+ pitting edema, and diminished sensation in a stocking and glove distribution. Laboratory results show elevated potassium, low carbon dioxide, high blood urea nitrogen, elevated creatinine, high glucose, elevated BNP, elevated CPK, and urinalysis with 3+ blood and protein, while imaging includes non-contrast CT scans of the head and neck, an ECG, point-of-care ultrasound of the lungs, echocardiography, and a chest radiograph.	Diagnosis	Congestive heart failure exacerbation
			Examination	Ventilation-perfusion scan of the chest
			Examination	B lines
Case 318	Paediatrics	A 17-day-old boy presents with respiratory distress, nonproductive cough, tactile fevers, poor feeding, intermittent cyanosis, and physical examination reveals pallor, respiratory distress with retractions, decreased air entry, dullness to percussion, and diffuse rhonchi on the right side. Laboratory findings show leukocytosis with no blood gas analysis available, and imaging reveals a motionless right hemidiaphragm on fluoroscopic evaluation.	Diagnosis	Diaphragmatic eventration
			Examination	Ultrasonography or fluoroscopy
			Treatment	A partially flattened hemidiaphragm
Case 319	Internal Medicine	A 21-year-old woman presents with 3 days of abdominal pain, dysuria, frequent urination, dark urine, and increased vaginal discharge, with physical examination revealing moderate suprapubic tenderness and numerous small erythematous papules and scattered pustules on the mons. Laboratory findings show a negative urine pregnancy test, urine analysis with 5-10 white blood cells, 25 red blood cells, few bacteria, positive leukocyte esterase, negative nitrites, and no trichomonads, clue cells, or yeasts on vaginal wet preparation.	Diagnosis	Cystitis
			Examination	Trimethoprim/sulfamethoxazole
			Treatment	Urine assay/sensitivity and GC/chlamydia assay
Case 320	Paediatrics	An 11-year-old girl with developmental delay presents with bilateral leg pain, ecchymosis, gingival hyperplasia, and pitting edema, appearing thin and pale with tachycardia. Laboratory results show severe anemia with hemoglobin at 5.9 g/dL, low iron levels, elevated reticulocyte count, and normal imaging of the tibia and fibula without evidence of thrombophlebitis.	Diagnosis	Scurvy
			Disease characteristics	A 47-year-old unhoused man with a history of alcohol abuse who presents with altered mental status, diffuse bruising on his legs, joint pain, and gum swelling

			Disease characteristics	Proptosis
Case 321	Internal Medicine	A 53-year-old man with obesity presents with substernal chest pain, a nagging dry cough, frequent fevers, night sweats, and mild bilateral pedal edema, with physical examination revealing tachycardia, pale appearance, and reduced air entry at the lung bases. Laboratory results show anemia, low sodium and potassium, elevated bicarbonate and blood urea nitrogen, normal troponin levels, and sinus tachycardia on ECG, with pending CT angiography results.	Diagnosis	Esophageal adenocarcinoma
			Examination	Esophageal perforation
Case 322	Minor	A 45-year-old woman was found unresponsive with agonal breathing, fixed pupils, and hypotension, showing no improvement in mental status after naloxone administration and requiring intubation. Laboratory results revealed elevated leukocyte count, creatinine, liver enzymes, lactic acid, ammonia, creatine phosphokinase, and troponin levels, with imaging showing no acute process but later chest radiography indicating bilateral lung opacities, leading to cardiac arrest and death on day 5.	Diagnosis	Calcium-channel blocker overdose
			Treatment	All of the above
			Treatment	Clonidine
Case 323	Internal Medicine	A 41-year-old man presents with a severe frontal headache, nausea, vomiting, subjective fevers, diplopia, photophobia, right eye ptosis, inferolateral deviation, dilated unreactive pupil, and bitemporal hemianopsia, with mild bilateral gynecomastia noted on examination. Laboratory results show leukocytosis with elevated neutrophils, CSF with high red and white blood cell counts, elevated protein, normal glucose, negative Gram stain, and imaging includes a CT and MRI of the brain.	Diagnosis	Pituitary tumor apoplexy
			Examination	All of the above
			Disease characteristics	MRI of the brain
Case 324	Major Surgery	A 73-year-old woman presents with fever, chills, night sweats, and rigors, appearing toxic with a temperature of 103°F, heart rate of 104 beats/min, stable blood pressure, and several nontender external hemorrhoids with strongly positive guaiac stool test. Laboratory results show a white blood cell count of 23.7×10^3 cells/μL with 98% neutrophils, hemoglobin of 8.2 g/dL, hematocrit of 24.6%, creatinine of 1.8 mg/dL, and blood cultures positive for alpha-hemolytic Streptococcus and Candida glabrata, while imaging reveals clear lung fields and repeat echocardiography	Diagnosis	Aortoenteric fistula
			Treatment	4-6 weeks
			Disease characteristics	Abdominal aortic aneurysm
Case 325	Internal Medicine	A 67-year-old nonverbal man presents with wheezing, increased work of breathing, chest rubbing, a systolic murmur, bilateral basilar crackles, wheezing, and pitting edema. Laboratory findings are unremarkable except for increased CO2 and lactate levels, and chest radiography shows nonspecific atelectasis changes.	Diagnosis	Foreign body aspiration
			Disease characteristics	Development of bronchiectasis or pneumonia
			Treatment	Right main stem bronchus
Case 326	Internal Medicine	A 45-year-old man presents with fever, sore throat, odynophagia, muffled voice, and tender cervical lymphadenopathy, with physical examination showing erythematous and edematous oropharynx without tonsillar exudates. Laboratory results reveal leukocytosis with neutrophil predominance, hypokalemia, and elevated liver transaminases, while imaging includes a neck radiograph prior to awake intubation and ICU transfer.	Diagnosis	Epiglottitis
			Examination	Visualization of the epiglottis on laryngoscopy
			Disease characteristics	A 45-year-old man with diabetes
Case 327	Internal Medicine	A 20-year-old active-duty male soldier presents with a 3-day history of a dark-red burning rash spreading from his sock line to his thighs and hands, accompanied by a sore throat, scratchy voice, and 1+ nonpitting edema in his hands and right ankle. Laboratory findings reveal urinalysis with 2+ protein and blood, 25-50 RBCs per high-power field, and skin biopsies showing small-vessel leukocytoclastic vasculitis with immunofluorescence indicating IgG, IgA, and C3 deposition.	Diagnosis	Henoch-Schönlein purpura
			Examination	Skin biopsy
			Disease characteristics	Most cases resolve within 1 month
Case 328	Internal Medicine	A 38-year-old man presents with bleeding gums, oral hemorrhagic blisters, and petechiae on his legs, with no	Diagnosis	Immune thrombocytopenic purpura

		history of trauma or systemic symptoms. Laboratory results show a platelet count of 15,000/µL, normal coagulation tests, negative HIV and hepatitis serologies, and a peripheral blood smear indicating decreased platelets and megakaryocytes.	Treatment	Observe the patient and obtain a repeat CBC in 1 to 2 weeks
			Treatment	Dexamethasone
Case 329	Internal Medicine	A 36-year-old man presents with fatigue, increased thirst, leg cramps, plethoric face, mild eye congestion, hypertension, 1+ pedal edema, and diminished air entry over the left lower lung, along with weight gain and facial flushing. Laboratory results show elevated blood glucose and glycated hemoglobin, low potassium, elevated fasting plasma cortisol, and ACTH levels, with lung imaging revealing left lower lobe atelectasis and opacity behind the heart, and failure of the high-dose dexamethasone suppression test.	Diagnosis	Cushing syndrome/endobronchial carcinoid with ectopic ACTH secretion
			Disease characteristics	Clinical syndrome of adrenal insufficiency
			Disease characteristics	Increasing hepatic glucose output
Case 330	Internal Medicine	A 32-year-old man presents with syncope, excessive sweating, tachycardia, elevated blood pressure, and mild anxiety, with a history of low blood glucose levels during fasting and after high-carbohydrate meals. Laboratory results reveal hypoglycemia with a fingerstick glucose level of 44 mg/dL, low insulin levels, normal C-peptide and proinsulin levels, elevated beta-hydroxybutyrate, and normal IGF-2, while imaging is not mentioned.	Diagnosis	Insulinoma
			Examination	3-6 months
			Treatment	68Ga-DOTA uptake test
Case 331	Internal Medicine	A 38-year-old woman presents with new-onset amenorrhea, mood swings, weight gain, easy bruising, and hypertension, with a physical examination showing no acute distress and normal thyroid size and texture. Laboratory results reveal low anti-müllerian hormone, low DHEA-S, low ACTH, elevated 24-hour free cortisol, and low potassium, while imaging shows a large left adrenal mass consistent with a lipid-rich adenoma.	Diagnosis	Cushing syndrome
			Examination	Lack of diurnal variation in blood cortisol levels
			Disease characteristics	Weight gain
Case 332	Internal Medicine	A 55-year-old man presents with nausea, vomiting, decreased libido, impotence, and appears apathetic and unwell, with fine perioral skin wrinkling, weak peripheral pulses, and a blood pressure of 100/70 mm Hg. Laboratory results show normochromic normocytic anemia and hyponatremia with a serum sodium of 126 mEq/L, while cranial MRI images are provided for further evaluation.	Diagnosis	Hypopituitarism due to empty sella syndrome
			Examination	Diuretic use
			Disease characteristics	Low free T4, normal TSH
Case 333	Internal Medicine	A 43-year-old man presents with recurrent episodes of hives, nausea, vomiting, cramping epigastric pain, and watery loose stools, with a normal physical examination. Laboratory results are unremarkable, but endoscopy reveals erythematous duodenopathy and duodenal biopsies show focal acute cryptitis with villous flattening, while a food diary suggests symptoms occur after eating red meat.	Diagnosis	Alpha-gal syndrome
			Treatment	Repeated alpha-gal IgE antibody measurement
			Treatment	Dietary elimination of pork, beef, lamb, and mammalian-derived products
Case 334	Minor	A 58-year-old construction site foreman presents with a gradual inability to maintain an erection over 2 years, moderate lower urinary tract symptoms, mild decreased libido, and physical examination shows a virilized male with normal-sized testes, moderately enlarged prostate, and obesity with a BMI of 32 kg/m². Laboratory results reveal a serum total testosterone level of 345 ng/dL, borderline high lipid levels, normal basic metabolic profile, and an erectile function domain score of 19 on the IIEF questionnaire.	Diagnosis	Organic erectile dysfunction
			Examination	Cardiac stress testing
			Treatment	All of the above
Case 335	Internal Medicine	An 18-year-old woman presents with 4 weeks of abdominal pain, bloating, excessive flatulence, and a 5-lb weight loss, with a soft, nontender abdomen and normal physical examination findings. Laboratory tests are unremarkable, but an upper endoscopy reveals prominent gastric folds, a nodular appearance in the antrum, and two duodenal ulcers.	Diagnosis	Helicobacter pylori infection
			Examination	Serology
			Treatment	Bismuth, tetracycline, metronidazole, PPI
Case 336	Minor	A 76-year-old man presents with right hemifacial spasms, diplopia, and impaired extraocular movements of the left eye, with a history of hemicranial headache	Diagnosis	Partial cranial nerve III palsy
			Disease characteristics	Compression of CN III

		and diplopia that have improved since hospitalization. Laboratory and imaging results include an unremarkable noncontrast head CT and mild bilateral carotid artery stenosis on Doppler study.	Treatment	Place an eye patch over the patient's unaffected eye
Case 337	Paediatrics	A 40-day-old male infant presents with irritability, fever, and a 4-cm indurated, nonfluctuant, tender swelling with erythema in the left subauricular and submandibular areas. Laboratory results show a peripheral leukocyte count of $10.2\times10^3/mm^3$ with 13% bands, and cerebrospinal fluid analysis reveals protein of 67 mg/dL, glucose of 59 mg/dL, leukocytes of $2/mm^3$, with negative Gram stain and agglutination studies.	Diagnosis	Cellulitis-adenitis syndrome
			Disease characteristics	All of the above
			Disease characteristics	Type III
Case 338	Internal Medicine	A 29-year-old woman presents with a 2-week history of fatigue, nausea, and muscle aches, with physical examination showing a temperature of 99.1°F, pulse of 95 beats/min, blood pressure of 102/60 mm Hg, and trace pedal edema. Laboratory results reveal elevated alanine aminotransferase at 221 U/L and aspartate aminotransferase at 60 U/L, with other routine labs within normal limits, and urine culture showing 20-50,000 colony-forming units/mL with no predominating organism.	Diagnosis	Acute hepatitis C
			Examination	All of the above
			Examination	Hepatitis C antibody and hepatitis C RNA
Case 339	Internal Medicine	A 70-year-old man presents with unintentional weight loss, worsening fatigue, and abdominal cramping, with physical examination revealing generalized abdominal distention and a palpable liver edge. Laboratory results show mild anemia with a hemoglobin level of 11.9 g/dL, and imaging via CT of the abdomen and pelvis reveals a 3.7-cm mass in the pancreas and moderate ascites.	Diagnosis	Primary pancreatic cancer
			Disease characteristics	BRCA2 mutation
			Examination	EUS
Case 340	Internal Medicine	A 79-year-old woman presents with left hip pain, difficulty walking, groin tenderness, and a left leg that is shortened and externally rotated. Laboratory results show a significantly depleted phosphate concentration, elevated alkaline phosphatase and parathyroid hormone levels, and imaging reveals a left femoral neck fracture.	Diagnosis	Oncogenic osteomalacia
			Examination	Low serum phosphate, raised urinary phosphate/creatinine ratio, normal serum calcium
			Examination	T2-weighted STIR MRI
Case 341	Paediatrics	A 9-year-old boy presents with intermittent fever, abdominal pain, failure to thrive, tiredness, weakness, slight pallor, and grade 1 digital clubbing, with physical examination showing slight tenderness in the left lumbar region. Laboratory results reveal anemia with hemoglobin of 10.7 g/dL, normal liver and kidney function tests, urine showing 3-4 WBCs per high power field, and a urine culture positive for over 100,000 CFU/mL of Escherichia coli sensitive to multiple antibiotics.	Diagnosis	Ureteropelvic junction obstruction
			Disease characteristics	Lack of peristalsis
			Examination	Any age group
Case 342	Internal Medicine	A 25-year-old woman presents with fever, diffuse pinkish rash, left lower-quadrant pain, nausea, vomiting, hypotension, tachycardia, tachypnea, and diffuse lung crackles, with physical examination showing a blanching rash and increased capillary refill. Laboratory results reveal a WBC count of 31.6×10^3 cells/µL, band neutrophils at 13%, creatinine at 2.4 mg/dL, and arterial blood gas showing pCO2 of 27 mm Hg, pO2 of 56 mm Hg, bicarbonate at 19 mEq/L, and a base deficit of 4.4 mmol/L, while chest radiography indicates changes consistent with	Diagnosis	Toxic shock syndrome
			Examination	All of the above are consistent with streptococcal TSS
			Treatment	Clindamycin plus vancomycin plus penicillin
Case 343	Paediatrics	A 3-year-old girl presents with fever, refusal to walk due to right leg pain, and localized tenderness over the L1-2 region of the back, with stable vital signs and normal neurologic examination. Laboratory results show elevated white blood cell count, erythrocyte sedimentation rate, and C-reactive protein, while plain x-rays are unremarkable and MRI scans are obtained for further evaluation.	Diagnosis	Psoas abscess
			Examination	MRI has been reported to be more sensitive than CT scanning
			Disease characteristics	The psoas muscle together with the iliacus muscle is referred to as the iliopsoas
Case 344	Internal Medicine	A 40-year-old male intravenous drug user presented with fever, shaking chills, a 3/6 diastolic murmur, and severe left leg pain, with physical examination revealing a pale, cool leg and no detectable pulses	Diagnosis	Fungal endocarditis
			Examination	Perform emergency valve replacement
			Treatment	Beta-D-glucan assay

		below the iliacs. Laboratory and imaging results showed a white blood cell count of 15,000 cells/µL with 90% polymorphonuclear leukocytes, creatinine level of 1.6 mg/dL, massive vegetation on the aortic valve, a large clot in the left femoral artery, and methicillin-resistant Staphylococcus aureus in blood cultures.		
Case 345	Minor	A 20-year-old woman presents with bilateral flank pain, episodic vomiting, malar angiofibromas, a leathery patch on her back, and periungual fibromas, with palpable cystic masses in the flanks. Laboratory results show anemia and urine abnormalities, while imaging reveals bilateral renal angiomyolipomas and subependymal calcified nodules in the brain.	Diagnosis<br>Disease characteristics<br>Disease characteristics	Tuberous sclerosis<br>Angiomyolipoma<br>Trunk and lower extremities
Case 346	Minor	A 32-year-old woman presents with several flesh-colored papules and tan oval macules on her face, trunk, and upper extremities, which are nontender, nonerythematous, and produce no discharge. Laboratory and imaging results are not provided in the case details.	Diagnosis<br>Diagnosis<br>Disease characteristics	Neurofibromatosis<br>Acute renal failure<br>Optic nerve tumors primarily occur in adolescents
Case 347	Psychiatry	A 19-year-old male presents with social withdrawal, poor hygiene, weight loss, flat affect, and paranoid delusions, including beliefs about being poisoned, alongside a family history of psychiatric hospitalization. Laboratory tests, including metabolic panel, CBC, thyroid function, liver tests, urinalysis, urine drug screen, and blood alcohol level, are normal, and an MRI of the head is unremarkable.	Diagnosis<br>Treatment<br>Treatment	Schizophrenia<br>Screening for diabetes<br>Family interventions
Case 348	Internal Medicine	A 33-year-old man presents with worsening dysphagia, inability to swallow saliva, and a history of esophageal reflux, with physical examination showing no acute distress or abnormal findings. Laboratory tests and chest radiographs are normal, and endoscopy reveals an esophageal foreign-body obstruction, leading to treatment with intravenous fluids, glucagon, and consultation for food bolus extraction.	Diagnosis<br>Disease characteristics<br>Examination	Eosinophilic esophagitis<br>Esophageal rings<br>Dysphagia is the most common presenting symptom
Case 349	Internal Medicine	A 24-year-old man presents with generalized weakness, progressive weight loss, intractable nausea, vomiting, and intermittent sharp epigastric pain, with physical examination showing a well-developed, malnourished individual in mild distress but otherwise normal findings. Laboratory tests are within normal limits, and imaging includes a CT of the abdomen and pelvis and upper gastrointestinal endoscopy, which are performed to evaluate the abdominal pain.	Diagnosis<br>Treatment<br>Treatment	Superior mesenteric artery syndrome<br>Gastric decompression<br>Laparoscopic duodenojejunostomy
Case 350	Internal Medicine	A 61-year-old woman with insulin-dependent diabetes, hypertension, hyperlipidemia, and a history of idiopathic pancreatitis presents with worsening intermittent epigastric pain, tenderness to deep palpation, and no other significant symptoms. Laboratory tests show normal serum amylase, lipase, liver enzymes, bilirubin, and CBC, with unremarkable chest radiography and ECG, while abdominal CT and MRCP imaging results are provided but not described.	Diagnosis<br>Examination<br>Treatment	Dorsal agenesis of the pancreas<br>MRCP or ERCP<br>Exogenous replacement of pancreatic enzymes
Case 351	Internal Medicine	A 52-year-old woman presents with severe abdominal pain, black vomit, bloody diarrhea, fever, tachycardia, hypotension, and abdominal tenderness with guarding. Laboratory results show leukocytosis, anemia, elevated creatinine and blood urea nitrogen, high erythrocyte sedimentation rate, elevated lactate dehydrogenase, and normal imaging findings.	Diagnosis<br>Disease characteristics<br>Disease characteristics	Mesenteric ischemia<br>Intracranial hemorrhage<br>Atherosclerosis
Case 352	Internal Medicine	A 24-year-old incarcerated Caribbean man presents with exertional dyspnea, fatigue, chronic nausea, and significant conjunctival pallor, with a soft pansystolic murmur over the tricuspid and aortic valves. Laboratory results show iron deficiency anemia with a hemoglobin level of 6.5 g/dL, low mean corpuscular volume, low ferritin, and normal colonoscopy and endoscopy except for nodular gastric mucosa, which was biopsied.	Diagnosis<br>Examination<br>Treatment	Idiopathic eosinophilic gastroenteritis<br>Demonstration of eosinophilic infiltration in the GI tract<br>Corticosteroids
Case 353	Internal Medicine	A 51-year-old woman presents with severe, diffuse abdominal pain occurring every 3-4 weeks, associated	Diagnosis	Primary epiploic appendagitis

		with nausea but no vomiting, and physical examination reveals a nontender, nondistended abdomen with normal bowel sounds. Laboratory tests including urinalysis, CBC, metabolic panel, amylase, and lipase are normal, and repeated abdominal and pelvic CT scans show no abnormalities.	Disease characteristics	Twisting, stretching, or kinking of the appendages, with impairment of the blood supply
			Disease characteristics	Abdominal pain
Case 354	Internal Medicine	A 48-year-old man presents with epigastric pain, nausea, nonbloody vomiting, anorexia, tachycardia, hypotension, and epigastric tenderness with decreased bowel sounds. Laboratory results show elevated white blood cell count, creatinine, blood urea nitrogen, glucose, lipase, amylase, triglycerides, and IgG4, while a CT scan reveals moderate peripancreatic edema and a septated cyst in the tail of the pancreas, consistent with acute pancreatitis.	Diagnosis	Triglyceride-induced pancreatitis
			Treatment	Fibrates
			Treatment	Insulin
Case 355	Internal Medicine	A 50-year-old woman with euthyroid Hashimoto thyroiditis presents with a goiter, brain fog, fatigue, and joint aches, with a physical examination showing a slightly firm thyroid gland of normal size and no palpable nodule. Laboratory and imaging results reveal a TSH level of 2.43 µIU/mL, elevated parathyroid hormone at 146 pg/mL, borderline elevated serum calcium, and a thyroid ultrasound showing a 1.1 cm hypoechoic nodule with unusual vascular patterns, while a technetium 99m sestamibi scan indicates isotope trapping in the right inferior thyroid lobe.	Diagnosis	Intrathyroidal parathyroid adenoma
			Examination	Sporadic parathyroid adenomas may carry a somatic MEN1 mutation, and about 30% have loss of heterozygosity of chromosome 11
			Disease characteristics	A feeding vessel, especially if associated with arborization of vascular flow within the nodule
Case 356	Minor	An 80-year-old man presents with gross hematuria, urinary hesitancy, suprapubic fullness, and tenderness, following a recent TURP procedure. Laboratory results show anemia with a hemoglobin level of 7.7 g/dL, elevated creatinine at 1.7 mg/dL, and imaging reveals bilateral hydronephrosis and a large bladder clot with a small hemorrhage.	Diagnosis	Clot retention at the prostatic fossa
			Examination	Retrograde ejaculation
			Disease characteristics	RBCs
Case 357	Major Surgery	A 32-year-old woman presents with diffuse hair loss on the scalp, increased hair growth on arms and face, amenorrhea, deepening voice, and clitoromegaly, with physical examination showing bitemporal hairline recession, vertex balding, and excessive hair on the upper extremities. Laboratory results reveal low luteinizing and follicle-stimulating hormones, elevated 17-hydroxyprogesterone, and significantly increased free and total testosterone levels.	Diagnosis	Steroid cell ovarian tumor
			Examination	Obtain levels of serum total testosterone, sex hormone binding globulin, DHEA-S, and beta-hCG
			Examination	Elevated testosterone and normal DHEA-S levels, and a negative beta-hCG test result
Case 358	Minor	A 19-year-old woman with a history of Wyburn-Mason syndrome presents with daily occipital headaches and severe weekly headaches accompanied by nausea and photophobia, with physical examination revealing a large subcutaneous AVM on the left side of her face, a right corneal AVM, facial numbness, and cervical muscle tenderness. Laboratory tests are normal, and a CT angiogram shows a large, complex facial AVM and one in the basal ganglia, with no vascular stenosis.	Diagnosis	Migraine without aura
			Treatment	Butalbital-APAP-caffeine
			Disease characteristics	Calcitonin gene-related peptide
Case 359	Minor	A 70-year-old woman with a history of chronic obstructive lung disease presents with increased coughing, shortness of breath, and later develops pain above the right heel, tenderness, redness, swelling around the Achilles tendon, and weakness to resisted plantar flexion of the right ankle. Laboratory and imaging results are not provided, but a gap is palpated in the middle third of the Achilles tendon, and she is unable to raise her right heel to stand on her toes on the right side.	Diagnosis	Achilles tendon rupture associated with fluoroquinolone administration
			Examination	Use of corticosteroids
			Disease characteristics	A positive Thompson test result
Case 360	Major Surgery	A 59-year-old woman presents with chest pain, dyspnea, and a gradual decline in platelet count post-femoral fracture surgery, with physical examination showing tachycardia and dry mucous membranes. Laboratory results reveal a platelet count drop to 33,000 cells/µL, elevated D-dimer and ferritin levels, prolonged partial thromboplastin time, and rouleaux	Diagnosis	Heparin-induced thrombocytopenia
			Treatment	Argatroban
			Treatment	Continue argatroban therapy until the platelet count is > 150,000 cells/µL and bridge to warfarin

		formation on peripheral smear, while CT angiography shows a segmental pulmonary embolism.		
Case 361	Internal Medicine	A 48-year-old woman presents with hoarseness, difficulty breathing, and a fixed, tender right supraclavicular lymph node, with physical examination revealing right-sided vocal cord paralysis. Laboratory tests are normal, while imaging shows FDG avidity in multiple lymph nodes and the left lung, diffuse FDG uptake in the left larynx, and a moderate pericardial effusion with right-sided chamber compression.	Diagnosis	Recurrent invasive ductal breast cancer
			Treatment	Fam-trastuzumab deruxtecan
			Treatment	Diarrhea
Case 362	Internal Medicine	A 46-year-old man with a history of head trauma presents with occasional dizziness, neck pain, and mild distress, later developing increased confusion and seizure, with physical examination showing normal heart and lung findings and no edema. Laboratory results reveal severe hyponatremia with a serum sodium level of 113 mEq/L, low plasma osmolality of 235 mOsm/kg, high urine osmolality of 1057 mOsm/kg, and a CT scan indicating brain edema.	Diagnosis	Syndrome of inappropriate antidiuretic hormone secretion
			Treatment	Urine sodium concentration of 13 mEq/L
			Diagnosis	Administer 100-mL bolus of 3% saline intravenously and repeat twice as needed to raise serum sodium by 6 mEq/L during the first 6 hours
Case 363	Internal Medicine	A 36-year-old man with HIV presents with hypotension, hypoxia, tachycardia, dyspnea, cachexia, and diffuse muscle wasting, with a firm and mildly distended abdomen but no facial swelling or urticaria. Imaging reveals a hyperdynamic heart, no right ventricular enlargement, a large amount of gastric fluid, and no pneumothorax or effusion, with pending laboratory results.	Diagnosis	Superior mesenteric artery syndrome
			Disease characteristics	Abdominal compartment syndrome
			Disease characteristics	Left renal vein
Case 364	Internal Medicine	A 75-year-old man presents with worsening lower abdominal pain, weight loss, bilateral lower-extremity swelling, abdominal distention, anuria, and numbness in the legs, with physical examination revealing hypertension, dry oral mucous membranes, abdominal distention, tenderness, and bilateral pitting edema. Laboratory results show hyponatremia, hypochloremia, low bicarbonate, severely elevated creatinine, and blood urea nitrogen, with a CT scan of the abdomen and pelvis obtained.	Diagnosis	Obstructive uropathy
			Disease characteristics	Right-sided flank pain with 102.1°F fever
			Treatment	Cetirizine
Case 365	Internal Medicine	A 24-year-old man presents with sudden right lower quadrant abdominal pain, nausea, and vomiting, with physical examination revealing significant tenderness in the right lower quadrant without guarding or rebound tenderness, and mild discomfort on percussion of the right costovertebral angle. Laboratory results show elevated creatinine, LDH, AST, and ALT levels, mild proteinuria, and normal troponin and ECG, while imaging with ultrasound and CT scan shows normal liver, gallbladder, kidneys, and appendix.	Diagnosis	Acute renal infarction
			Disease characteristics	High serum LDH level
			Examination	Cardioembolic source
Case 366	Minor	A 28-year-old man with systemic lupus erythematosus presents after a suicide attempt with pill ingestion, showing nausea, abdominal pain, fever, hypotension, tachycardia, and prolonged QTc on ECG, with a Glasgow Coma Scale score of 15/15. Laboratory results reveal hypokalemia (3.1 mmol/L), which worsened to 2.5 mmol/L, and imaging shows polymorphic ventricular tachycardia that spontaneously resolved before treatment with magnesium sulfate and diazepam.	Diagnosis	Hydroxychloroquine overdose
			Disease characteristics	As the intracellular shift corrects, the patient's serum potassium levels rise
			Treatment	Calcium gluconate
Case 367	Internal Medicine	A 46-year-old woman with a history of congenital splenomegaly, thrombocytopenia, asthma, bipolar disorder, migraines, and recurrent DVT presents with worsening lung infiltrates, fatigue, fevers, oral ulcerations, joint swelling, and Raynaud phenomenon, with physical examination revealing oral ulcerations, decreased breath sounds, crackles, and joint swelling with erythema. Laboratory results show leukopenia, thrombocytopenia, anemia, elevated inflammatory markers, positive antinuclear and dsDNA antibodies, low complement levels, positive lupus anticoagulant, and imaging reveals right-sided multifocal patchy	Diagnosis	Antiphospholipid syndrome nephropathy
			Examination	Warfarin
			Treatment	ADAMTS13 activity testing

		ground glass opacities and a multiloculated effusion, while renal biopsy indicates acute thrombotic microangiopathy		
Case 368	Internal Medicine	An 88-year-old woman presents with confusion, hallucinations, moaning, tachycardia, hypotension, diminished breath sounds, and a sacral decubitus ulcer with discharge. Laboratory results show leukocytosis, elevated creatinine, high blood urea nitrogen, hypercalcemia, elevated lactic acid, and troponin, while imaging reveals extensive left lower extremity DVT and a pulmonary embolism in the right lower lobe pulmonary artery.	Diagnosis	Primary hyperparathyroidism
			Examination	Albumin level
			Treatment	Intravenous fluids
Case 369	Internal Medicine	A 50-year-old woman presents with a pruritic migratory eruption on the trunk and proximal extremities, mild peripheral eosinophilia, and a history of hypothyroidism, IBS, and allergies, without arthralgia or neurologic symptoms. Laboratory testing is minimal due to her stable vital signs and history, with no additional lesions found on examination.	Diagnosis	Urticaria
			Treatment	Perform a biopsy
			Examination	A prescription for an EpiPen
Case 370	Minor	A 60-year-old woman presents with left-sided weakness, severe occipital headache, vomiting, feverish feeling, drowsiness, disorientation, meningismus, positive Brudzinski sign, decreased muscle strength on the left side, brisk reflexes, left hemianesthesia, and left homonymous hemianopsia. Laboratory findings show elevated leukocyte count, elevated erythrocyte sedimentation rate, hyponatremia, and cerebrospinal fluid with elevated protein, decreased glucose, and lymphocytic pleocytosis, while CT imaging reveals a hypodense area in the right middle cerebral artery distribution consistent with a cerebral infarction.	Diagnosis	Tuberculous meningitis with vasculitis
			Examination	Hypodense lesion with edema out of proportion to the mass effect on contrast-enhanced CT of the brain
			Examination	CSF ADA levels
Case 371	Internal Medicine	A 94-year-old man presents with altered mental status, confusion, bradycardia, hypothermia, and guaiac-positive stool, with physical examination showing sluggishly reactive pupils and faint distal pulses. Laboratory and imaging results reveal a bradycardic junctional rhythm with prolonged QT interval on ECG, hemoglobin of 8.5 g/dL, stable stage 4 chronic kidney disease, normal cardiac enzymes and urinalysis, mild vascular congestion on chest radiography, and no acute disease on head CT, with a blood glucose level dropping to 49 mg/dL.	Diagnosis	Myxedema coma
			Treatment	Stress-dose steroids
			Treatment	Intravenous levothyroxine bolus
Case 372	Internal Medicine	A 75-year-old man presents with fatigue, left upper quadrant pain, trace lower extremity edema, a 3/6 systolic murmur, and worsening vision, along with impairments of right eye adduction and left eye abduction. Laboratory findings reveal leukocytosis with atypical lymphocytes, a MAP2K1 mutation, and atypical hairy lymphoid cells in the CSF, while imaging shows marked splenomegaly and bilateral cerebral hemorrhaging.	Diagnosis	Hairy cell leukemia variant
			Disease characteristics	Rituximab
			Treatment	The absence of a BRAF V600E mutation is an indicator of HCL-v instead of typical HCL
Case 373	Minor	A 30-year-old man presents with acute weakness in all extremities, vision loss in the right eye, mild ocular pain, bladder dysfunction, spastic paraplegia, upper extremity diparesis, hyperreflexia, and a sensory level at C6/C7, with optic atrophy and mild disc pallor on funduscopic examination. Laboratory findings show a normal complete blood count, elevated erythrocyte sedimentation rate, normal liver and renal function tests, negative autoimmune and infectious serologies, and cerebrospinal fluid analysis reveals elevated protein and neutrophils, while MRI shows altered signals in the right optic nerve and patchy abnormal signals in the cervicothoracic spine.	Diagnosis	Neuromyelitis optica
			Disease characteristics	Periventricular white matter lesions
			Treatment	Interferon
Case 374	Internal Medicine	A 46-year-old woman presents with progressive weakness, dyspnea, leg swelling, dysphagia, loss of taste, hair loss, and a nontender ecchymotic rash, with physical examination revealing subconjunctival hemorrhaging, periorbital ecchymoses, tongue swelling, jugular venous distention, and bilateral edema.	Diagnosis	Cardiac amyloidosis
			Treatment	Tissue biopsy
			Examination	Angiotensin-converting enzyme inhibitors

		Laboratory and imaging results show elevated B-type natriuretic peptide, mildly elevated cardiac enzymes, cardiomegaly with bilateral pleural effusion and pericardial effusion on CT, mild proteinuria, and ECG with low-voltage complexes.		
Case 375	Internal Medicine	A 26-year-old man presents with a 2-month history of lower back pain and stiffness, worse in the morning and improving with activity, along with decreased lumbar spine flexion and mild chest expansion limitation. Laboratory results show a normal chemistry panel, slightly low platelet count, elevated erythrocyte sedimentation rate, positive HLA-B27, and lumbar spine radiographs are provided.	Diagnosis	Ankylosing spondylitis
			Disease characteristics	Thoracic kyphosis may be reduced by maintaining an erect posture and sleeping on a firm mattress
			Treatment	More than 90% of White patients with the disease are positive for the HLA-B27 gene
Case 376	Minor	A 36-year-old man with a history of type 1 diabetes presents with lingering dull ache pain at fracture sites in the left lower extremity following a waterskiing accident, with normal physical examination findings including normal gait and strength. Laboratory tests show normal electrolyte levels and complete blood cell count, while imaging reveals healing fractures without unexpected abnormalities, and electromyography and nerve conduction studies are normal.	Diagnosis	Fracture pain that does not qualify as chronic pain
			Treatment	Nonpharmacologic therapies can have some adverse effects and can achieve moderate efficacy
			Disease characteristics	Underlying diabetes
Case 377	Minor	A 51-year-old woman presents with progressive memory loss, difficulty with orientation, and anxiety, scoring 24/30 on the Mini-Mental State Examination with moderate deficits in orientation, memory, and visual-spatial skills. Laboratory tests are normal, while brain MRI shows generalized atrophy with notable bilateral hippocampal atrophy, and CSF analysis reveals elevated tau levels and a low amyloid beta (1-42) tau index.	Diagnosis	Alzheimer's disease
			Diagnosis	Atrophy on brain MRI
			Examination	Familial Alzheimer's disease
Case 378	Internal Medicine	A 58-year-old woman presents with bilateral sternoclavicular joint swelling, morning stiffness, dry eyes, and restricted shoulder movement, with significant knee and lower back pain. Laboratory tests show positive ANA with a nucleolar pattern, elevated CRP, and high ESR, while imaging reveals mild osteoarthrosis in the shoulder and knees, and MRI indicates active sacroiliitis with periarticular edema and mild erosion.	Diagnosis	Ankylosing spondylitis
			Treatment	High-dose NSAID therapy for 2 weeks is considered an adequate trial
			Treatment	TNF inhibitors are highly effective in reducing the inflammatory component of axial SpA, including peripheral synovitis, enthesitis, and uveitis, and in improving spinal mobility and function and overall quality of life
Case 379	Minor	A 49-year-old man with a history of osteopenia and HIV presents with a midshaft femoral fracture, HIV-treatment induced lipodystrophy, and a Cushingoid habitus, but normal vital signs and physical examination findings. Laboratory tests show normal blood counts, metabolic panel, hormone levels, and undetectable HIV viral load, while imaging reveals normal bone mineral density despite a decrease from previous levels.	Diagnosis	Alendronate therapy
			Disease characteristics	Decreased bone remodeling leading to the accumulation of microfractures
			Disease characteristics	The fracture is comminuted
Case 380	Internal Medicine	A 68-year-old woman presents with a 4-month history of intermittent epigastric pain, nausea, and vomiting, with physical examination revealing mild distress, a soft and moderately distended abdomen, mild tenderness in the epigastric region, palpable spleen, and decreased bowel sounds. Laboratory results show elevated serum lipase at 730 U/L, while imaging reveals an edematous and thickened jejunum and duodenum with adjacent free fluid and splenomegaly, similar to a previous CT scan.	Diagnosis	Acquired C1-inhibitor (C1-INH) deficiency
			Examination	Oropharynx
			Disease characteristics	C4 level
Case 381	Internal Medicine	A 36-year-old woman with type 1 diabetes and systemic lupus erythematosus presents with intractable nausea, occasional vomiting, diffuse abdominal pain, dry mucous membranes, decreased skin turgor, and paradoxical sinus arrhythmia. Laboratory results show hypokalemia, elevated blood urea nitrogen, creatinine, glucose, A1c, urine ketones, and positive cannabinoid	Diagnosis	Gastroparesis
			Treatment	Small, frequent meals (small-particle, low-fat diet)
			Treatment	Metoclopramide intranasal 15 mg up to four times a day as needed

		urine toxicology, while imaging reveals Los Angeles grade C esophagitis with no mechanical gastric outlet obstruction.		
Case 382	Internal Medicine	A 72-year-old man presents with facial and neck swelling, scleral and sublingual icterus, and unintentional weight loss, with physical examination revealing bilateral submandibular and lacrimal gland swelling. Laboratory tests show elevated liver enzymes, high bilirubin levels, and low albumin, while MRI indicates diffuse pancreatic enlargement and biopsy reveals lymphoid and plasmacytic infiltrate with storiform fibrosis, consistent with autoimmune pancreatitis.	Diagnosis	Immunoglobulin G4 related disease
			Disease characteristics	The most common intrinsic renal manifestation is tubulointerstitial nephritis
			Examination	Lymphoplasmacytic infiltration
Case 383	Internal Medicine	A 68-year-old man presents with severe diarrhea, neurogenic bladder, fatigue, 50-lb weight loss, postural dizziness, syncopal episodes, urinary urgency, nocturia, and physical examination reveals thin appearance, low blood pressure with orthostatic changes, and 2+ peripheral edema. Laboratory results show anemia, thrombocytopenia, low serum albumin, low total protein, elevated creatinine, bland urinalysis, and renal ultrasonography reveals a distended bladder, while serum immunofixation identified an IgA lambda monoclonal band.	Diagnosis	Amyloid light chain amyloidosis
			Disease characteristics	NT-proBNP, 24-hour urine protein, eGFR, and dFLC
			Examination	Nephrotic syndrome
Case 384	Minor	A 16-year-old boy presents with progressive quadriparesis, dysesthesia, flaccid hyporeflexia, and bilateral mute plantar response following a respiratory infection, later developing drowsiness and generalized tonic-clonic seizures. Laboratory and imaging results show normal blood counts and liver function, nerve conduction studies indicating demyelination, normal cervical spine MRI, brain MRI with bilateral asymmetric high signal intensity in subcortical and deep white matter, elevated CSF protein with lymphocytic pleocytosis, and EEG consistent with diffuse encephalopathy.	Diagnosis	Acute disseminated encephalomyelitis
			Treatment	High-dose IV corticosteroids
			Disease characteristics	Demyelination
Case 385	Minor	A 35-year-old man presents with jerky, nonpurposeful movements, a dancelike gait, memory impairment, and behavioral changes including anger and insomnia, with physical examination showing choreoathetoid movements and a flat affect. Laboratory tests are normal, but MRI of the brain reveals bilateral caudate atrophy with increased intercaudate distance.	Diagnosis	Huntington disease
			Disease characteristics	Tetrabenazine
			Treatment	Neuropsychiatric manifestations
Case 386	Minor	A 34-year-old woman presents with a 4-year history of psychotic behavior, neurocognitive decline, progressive motor regression, loss of speech, bowel and bladder incontinence, and spastic quadriparesis with hyperreflexia and bilateral extensor plantar response. Laboratory tests are largely unremarkable, while MRI of the brain shows abnormal signal intensity in the white matter of both cerebral hemispheres, predominantly in the posterior parietal and occipital lobes, with mild generalized cerebral atrophy.	Diagnosis	Leukodystrophy
			Disease characteristics	Occipital lobe
			Examination	Autosomal recessive
Case 387	Minor	A 42-year-old woman presents with shortness of breath, slurred speech, dysphagia, and bulbar muscle weakness, accompanied by ptosis and motor strength of 4/5, with normal reflexes and cerebellar testing. Laboratory results are unremarkable, CT and MRI of the brain show no abnormalities, and arterial blood gas reveals respiratory acidosis with pH 7.34, pCO2 48 mm Hg, and pO2 55 mm Hg.	Diagnosis	Myasthenia gravis
			Treatment	Prednisone with pyridostigmine
			Treatment	Intubate the patient
Case 388	Minor	A 57-year-old man presents with difficulty walking due to clumsiness, intermittent numbness and tingling in his extremities, decreased strength in his arms and legs, diminished reflexes, absent vibratory sensation up to his knees and elbows, decreased touch sensation in fingers and toes, and a positive Romberg sign. Laboratory and imaging results are not provided in the case details.	Diagnosis	Chronic inflammatory demyelinating polyneuropathy
			Examination	EMG
			Treatment	IV steroids
Case 389	Minor	A 78-year-old man presents with sudden inability to speak or swallow, wide-based gait, mild ataxia, brisk	Diagnosis	Foix-Chavany-Marie syndrome

		right-sided reflexes, right Babinski sign, bilateral cranial nerve deficits, anarthria, drooling, severe dysphagia, increased jaw jerk, and absent gag reflex. Laboratory tests are normal, ECG shows atrial fibrillation, and MRI reveals multiple bilateral infarcts in the frontal, parietal, and occipital lobes, along with cerebellar and cortical atrophy.	Diagnosis	Dissociation of automatic and voluntary movements of the bulbar muscles
			Disease characteristics	Poor, with need for percutaneous endoscopic gastrostomy (PEG) tube feeding
Case 390	Minor	A 72-year-old man with Parkinson's disease presents with episodic altered mental status, agitation, insomnia, visual and occasional auditory hallucinations, impaired short-term memory, and executive dysfunction, along with mild rigidity, bradykinesia, and postural instability. Laboratory tests are normal except for elevated cholesterol, and a CT scan shows scattered lacunar infarcts and mild cortical atrophy without ventricular dilatation.	Diagnosis	Dementia with Lewy bodies
			Disease characteristics	Recurrent visual hallucinations
			Treatment	Haloperidol
Case 391	Paediatrics	A 3-year-old boy presents with nightly fever, cervical lymphadenopathy, erythematous macular rash, joint pain, irritability, and mild hepatosplenomegaly, with physical examination revealing large cervical lymph nodes and reluctance to bear weight. Laboratory findings show leukocytosis, thrombocytosis, anemia, elevated ESR, CRP, transaminases, and ferritin > 10,000 ng/L, with negative infectious studies and normal echocardiography, leading to a diagnosis of incomplete Kawasaki disease and subsequent treatment with IVIG and aspirin.	Diagnosis	Macrophage activation syndrome
			Disease characteristics	In patients with SoJIA, MAS may be triggered by treatment for the disease
			Examination	Increased ferritin level
Case 392	Psychiatry	A 9-year-old boy with a history of asthma presents with anxiety symptoms, including nightmares, chest tightness, and difficulty breathing, particularly in social situations, despite normal physical examination findings. Laboratory and imaging results are not provided in the case details.	Diagnosis	Panic disorder without agoraphobia
			Diagnosis	Cognitive-behavioral therapy
			Treatment	Separation anxiety disorder
Case 393	Major Surgery	A 33-year-old woman presents with sudden dyspnea, abdominal pain, distention, and moderate tenderness, along with rapid shallow breathing and bilateral decreased breath sounds on examination. Laboratory results show leukocytosis, elevated hemoglobin and hematocrit, hyponatremia, and hyperkalemia, while imaging reveals bilateral pleural effusions and transvaginal ultrasound findings consistent with ovarian hyperstimulation syndrome.	Diagnosis	Ovarian hyperstimulation syndrome
			Disease characteristics	Cardiac tamponade
			Disease characteristics	Bimanual pelvic examination
Case 394	Internal Medicine	A 73-year-old woman presents with generalized weakness, lack of appetite, and is found to be frail, pale, and malnourished, with a heart rate of 106 beats/min, blood pressure of 156/76 mm Hg, and a grade II/VI systolic murmur, along with heme-positive stool. Laboratory results show hemoglobin of 6.8 g/dL, mean corpuscular volume of 63 fL, and platelets of 644,000/mm³, while imaging reveals asymmetric nodular wall thickening and abnormal dilation of the mid/distal jejunum, with a lesion identified on capsule endoscopy.	Diagnosis	Small bowel tumor
			Disease characteristics	Lobular breast carcinomas are more likely to cause them than ductal breast carcinomas
			Examination	Any of the above except proceed conservatively with continued observation without any intervention
Case 395	Major Surgery	A 37-year-old woman presented with multiple breast lumps, nipple retraction, peau d'orange, and palpable lymph nodes in the right axilla, with previous excisions of benign lumps and no family history of breast cancer. Imaging revealed marked skin thickening, multiple masses with prominent right axillary nodes, and numerous enhancing mass lesions on CEMRI, with a biopsy confirming fibroadenomas.	Diagnosis	Primary breast cancer
			Examination	Tru-Cut biopsy of the retroareolar region and the lymph node
			Examination	All of the above
Case 396	Internal Medicine	A 46-year-old woman presents with severe, constant abdominal pain, moderate distress, tachycardia, and diffuse abdominal tenderness, with a history of polycystic ovarian syndrome and previous deep venous thrombosis. Laboratory results show anemia, thrombocytosis, low iron saturation, and a positive t(9;22) cytogenetic analysis, while imaging reveals hemorrhagic products in the left pelvic region likely due to a ruptured ovarian cyst.	Diagnosis	Chronic myeloid leukemia
			Treatment	Assess for compliance and drug interactions
			Treatment	Ponatinib

Case 397	Minor	A 76-year-old man with a history of multiple basal cell carcinomas presents with an erythematous 6 mm lesion on his right lateral lower eyelid, with a central scar from a prior biopsy. Laboratory studies are normal, imaging shows no metastatic disease, and histopathology reveals intraepidermal basophilic atypical cells positive for cytokeratin 20, high-molecular-weight cytokeratin, synaptophysin, and BerEp4, but negative for CK5/6, S100, melan-A, carcinoembryonic antigen, and CD45.	Diagnosis	Merkel cell carcinoma in situ
			Disease characteristics	Epidermal Merkel cell carcinoma can demonstrate pagetoid upward migration, Pautrier-like microabscesses, and squamous or glandular differentiation
			Examination	Synaptophysin
Case 398	Internal Medicine	A 42-year-old woman presents with gum bleeding, progressive weakness, and bruising, with physical examination showing pale sclerae, ecchymotic patches, and petechiae. Laboratory results reveal moderate normocytic normochromic anemia, thrombocytopenia, elevated LDH, elevated beta-2 microglobulin, and a leukoerythroblastic blood film, while imaging includes a skull radiograph.	Diagnosis	Nonsecretory myeloma
			Disease characteristics	A high plasma cell labeling index
			Disease characteristics	Treatment options are similar to classic multiple myeloma
Case 399	Internal Medicine	A 55-year-old man presents with numbness, tingling, pain in extremities, progressive asymmetrical leg weakness, left foot drop, paraparesis with areflexia, and bilateral glove-and-stocking sensory loss. Laboratory and imaging results show normal CBC, liver and renal function tests, elevated CSF protein, chronic demyelinating polyneuropathy on nerve studies, and serum protein electrophoresis revealing a monoclonal protein spike with elevated IgM levels.	Diagnosis	Monoclonal gammopathy of undetermined significance (MGUS)
			Disease characteristics	IgM
			Diagnosis	Lytic bone lesions
Case 400	Internal Medicine	A 73-year-old woman on a low-carbohydrate diet presents with abdominal cramping, pain, constipation, and slight abdominal distension, with normal vital signs and no depression indicated by PHQ-9. Laboratory tests show normal electrolyte levels and complete blood cell count, while an abdominal CT scan reveals stool and gas throughout the colon without masses or dilation.	Diagnosis	Constipation
			Treatment	Modifying diet
			Disease characteristics	Bowel impaction and appendicitis
Case 401	Internal Medicine	A 35-year-old woman with Crohn's disease in remission presents with colicky abdominal pain, occasional vomiting, headaches, wheezing, and generalized abdominal tenderness without guarding or rebound tenderness. Laboratory results show elevated WBC count, ESR, CRP, and liver enzymes, while CT imaging reveals mild hepatomegaly, mild rectal and sigmoid submucosal inflammation, and increased large bowel thickness.	Diagnosis	Pneumatosis cystoides intestinalis
			Disease characteristics	Trichloroethylene
			Disease characteristics	Both can be treated with pirfenidone
Case 402	Paediatrics	A 10-year-old boy presents with painful, progressively enlarging swelling over the upper back and left chest, restricting left shoulder movement, with mild pallor and multiple nontender, hard bony swellings over the neck and upper back. Laboratory results are normal, with an erythrocyte sedimentation rate of 30 mm/hr, and imaging includes radiographs of the cervical spine, hand, pelvis, thigh, and knee, along with a CT scan of the swelling.	Diagnosis	Fibrodysplasia ossificans progressiva
			Diagnosis	Fibrodysplasia ossificans progressiva
			Examination	Skeletal survey (including radiographs of cervical spine, pelvis, limbs, and feet)
Case 403	Internal Medicine	A 60-year-old woman presents with painless macroscopic hematuria, malaise, and unintentional weight loss, with physical examination revealing a 2/6 systolic murmur and no gynecologic abnormalities. Laboratory tests show normal hemoglobin, platelet count, and electrolytes, urine cytology is positive for malignant cells, cystoscopy shows normal bladder mucosa, and CT of the abdomen and pelvis is performed.	Diagnosis	Urothelial carcinoma
			Disease characteristics	Disease-specific annual mortality is greater in men than in women.
			Examination	CT urography
Case 404	Internal Medicine	A 43-year-old woman with obesity presents with dyspnea, heart palpitations, chest tightness, and an intermittent productive cough, along with physical examination findings of bilateral decreased air entry and scattered wheezes, and skin dryness in flexural foldings. Laboratory results show blood eosinophils >10%, IgE level of 800 kU/L, and pulmonary function tests indicating obstructive lung disease with air trapping, while imaging reveals hyperlucent areas on bilateral	Diagnosis	Asthma
			Diagnosis	Optimized high-dose ICS-LABA treatment
			Treatment	Asthma that worsens if high-dose treatment is decreased

		lower lobes and a chest CT scan consistent with these findings.		
Case 405	Internal Medicine	A 19-year-old male presents with palpitations, syncope, and multiple bilateral macular erythematous lesions with central pallor on his thighs, along with an irregular, tachycardic rhythm on cardiac examination. Laboratory and imaging results include an ECG showing irregular heartbeats with no other specific abnormalities noted.	Diagnosis<br>Disease characteristics<br>Treatment	Disseminated Lyme disease<br>Vancomycin<br>Cardiac conduction defects
Case 406	Psychiatry	A 17-year-old boy presents with hallucinations, delusions, paranoia, and acute excitability, with a flat affect and suspicion during the interview, alongside a history of an acute depressive episode and family history of bipolar disorder. Laboratory tests, including urine drug screening, CBC, vitamin B12, renal and liver function tests, thyroid profile, and copper levels, are normal, and a brain CT scan shows no lesions or evidence of trauma.	Diagnosis<br>Disease characteristics<br>Disease characteristics	Schizophrenia<br>"I am the greatest musician of all time, and everyone loves me for my amazing work"<br>The appearance of positive and negative symptoms
Case 407	Minor	A 22-year-old man presents in a semiconscious state with pinpoint pupils, diffuse skin flushing, and a respiratory rate of 7 breaths/min, while vital signs and physical examination are otherwise unremarkable. Laboratory results show normal CBC, metabolic panel, coagulation profile, and urinalysis, with a chest radiograph prompting abdominal radiography.	Diagnosis<br>Treatment<br>Treatment	Opiate overdose<br>Naloxone<br>Leaking cocaine packet
Case 408	Internal Medicine	A 55-year-old woman presents for a routine physical examination with a history of hypercholesterolemia and hypertension, showing normal vital signs, clear breath sounds, and no abnormalities in skin, heart, abdomen, extremities, or neurological examination. Laboratory results indicate normal electrolyte levels and complete blood cell count, with total cholesterol at 190 mg/dL, LDL at 120 mg/dL, HDL at 52 mg/dL, and triglycerides at 140 mg/dL, and no imaging tests were conducted.	Diagnosis<br>Disease characteristics<br>Treatment	Stage 1 hypertension<br>It can potentiate vascular inflammation<br>By protecting elastic fibers from calcium deposition
Case 409	Minor	A 57-year-old man with type 2 diabetes and hypertension presents with a 2-week history of extremely pruritic, skin-colored vesiculobullous lesions on his feet, hands, and fingers, accompanied by nonpitting edema and reduced sensation in his legs. Physical examination shows well-appearing vital signs, with fluid-filled vesicles on the instep and plantar aspects of his feet and palms, without surrounding erythema, and no other lesions or abnormalities noted.	Diagnosis<br>Disease characteristics<br>Disease characteristics	Dyshidrotic eczema<br>Person-to-person contact<br>Hands
Case 410	Paediatrics	A 7-year-old obese boy presents with left hip and knee pain, decreased range of motion, tenderness, and an antalgic gait, following a fall and a history of worsening limp and mobility over two months. Radiographs reveal abnormal medial metaphyseal beaking of the left proximal tibia, mild flattening of the epiphysis medially, and interval changes in the left hip compared to previous imaging, consistent with juvenile Blount disease.	Diagnosis<br>Disease characteristics<br>Diagnosis	Legg-Calvé-Perthes disease<br>Children may present with knee pain<br>Sickle cell anemia
Case 411	Paediatrics	An 18-month-old girl with a history of viral cardiomyopathy presents with nighttime diaphoresis, agitation, and a cardiac examination revealing a lateralized point of maximal intensity, a fourth heart sound, and a holosystolic murmur of mitral regurgitation. Laboratory and imaging results show cardiomegaly on chest radiography, sinus tachycardia with ischemic changes on ECG, a dilated left ventricle with an ejection fraction of 15% on TTE, and a cardiac CT angiography performed for coronary anatomy assessment.	Diagnosis<br>Disease characteristics<br>Disease characteristics	Anomalous coronary artery<br>Feeding intolerance<br>Mitral regurgitation
Case 412	Paediatrics	An 8-year-old boy with autism and lupus presents with congestion, cough, fever, ear pain, chest pain, orthopnea, and shortness of breath, with physical examination revealing respiratory distress, absence of breath sounds on the left, diminished breath sounds with crackles on the right, tachypnea, tachycardia, café au lait spots, conjunctival erythema, and distended neck	Diagnosis<br>Disease characteristics<br>Examination	Acute lymphoblastic leukemia/lymphoma<br>Glioblastoma<br>Whole body MRI beginning at 6 years of age; continue once per year

		veins. Laboratory and imaging results show a large left pleural effusion with mediastinal shift, a normal WBC count, elevated ESR, and a chest CT revealing a large anterior mediastinal mass with malignant cells in the pleural fluid.		
Case 413	Minor	A 48-year-old man presents with severe burning pain during urination and hematuria after a fall, with physical examination showing blood at the urethral meatus but no external lacerations or scrotal abnormalities. Laboratory tests reveal gross hematuria with normal blood counts and coagulation profile, while imaging shows normal pelvic radiographs and a retrograde urethrogram is performed.	Diagnosis	Urethral injury
			Disease characteristics	Posterior urethral injuries are commonly associated with pelvic fractures
			Disease characteristics	Urinary retention
Case 414	Minor	A 59-year-old woman presents with asymptomatic, nonhomogeneous leukoplakia on the maxillary gingiva, showing multifocal thin, thick, and verrucous plaques that do not slough off upon rubbing, with no extraoral abnormalities or lymphadenopathy. Biopsies reveal marked hyperkeratosis with mild epithelial dysplasia, and serial images show the lesions are enlarging and evolving in texture.	Diagnosis	Proliferative verrucous leukoplakia
			Examination	Reassess the diagnosis and consider a biopsy of the new lesions
			Treatment	Both clinical and histopathologic examination
Case 415	Psychiatry	A 30-year-old man presents with recent personality changes, including irritability and impatience, but has normal vital signs, neurologic examination, and mental status examination, with no evidence of hallucinations or delusions. Laboratory tests show normal complete blood count, hematocrit, hemoglobin, red blood cell count, and electrolyte levels, and a PHQ-9 score of 0, indicating no depression.	Diagnosis	Sleep disruption
			Disease characteristics	Short sleep latency indicates sleep deprivation
			Examination	Wearable fitness device that measures sleep stages
Case 416	Major Surgery	A 58-year-old woman with hypertension presents with a firm, movable mass in the left axilla and a small left-sided pleural effusion, but no breast abnormalities or other symptoms. Laboratory tests are normal except for mildly elevated alkaline phosphatase, and imaging shows a possible pleural effusion with biopsy revealing moderately well-differentiated adenocarcinoma that is CK 7+/CK 20-, TTF-1 negative, and estrogen receptor positive.	Diagnosis	Breast
			Disease characteristics	MRI
			Examination	Lung
Case 417	Minor	A 30-year-old woman presents with a 2-week history of new-onset headaches that worsen when lying down, with a physical examination revealing mild acne and papilledema in her right eye. Laboratory tests show normal electrolyte levels and complete blood cell count, while brain CT results are normal.	Diagnosis	Idiopathic intracranial hypertension
			Examination	Avoiding the potential complication of a CSF leak
			Treatment	Brain MRI
Case 418	Internal Medicine	A 66-year-old man presents with bilateral, palpable, nontender neck masses, poor dental hygiene, and hypochromic, microcytic anemia, with no systemic symptoms or organomegaly. Laboratory results show a normal WBC count and mildly elevated ESR, while a contrast-enhanced CT scan of the neck reveals bilateral neck masses.	Diagnosis	Scrofula
			Disease characteristics	PPD can indicate an exposure to M tuberculosis
			Examination	15%
Case 419	Internal Medicine	A 31-year-old primigravid woman at 37 weeks' gestation presents with left-sided chest pain, nonproductive cough, hemoptysis, dyspnea, diminished left hemithorax breath sounds, and end-expiratory wheezing. Laboratory and imaging results show normal blood counts, small bilateral pleural effusions on chest radiography, multiple thin-walled pulmonary cysts on high-resolution CT, elevated vascular endothelial growth factor D, and bilateral kidney cysts on abdominal CT.	Diagnosis	Lymphangioleiomyomatosis
			Examination	Sirolimus
			Treatment	Biopsy
Case 420	Minor	A 14-year-old boy presents with progressive weakness, hoarse voice, shortness of breath, diminished strength, loss of vibratory sense, and absent deep tendon reflexes, with vital signs showing tachycardia, hypertension, and hypoxemia. Laboratory results are normal, except for elevated CSF protein, and MRI imaging is performed for further evaluation.	Diagnosis	Guillain-Barre syndrome
			Examination	Elevated opening pressures
			Disease characteristics	Absent deep tendon reflexes in the lower extremities
Case 421	Psychiatry		Diagnosis	Alcohol use disorder

		A 60-year-old woman presents with sleep problems, low mood, anhedonia, mild agitation, and minimal eye contact, with a history of depression, diabetes, and increased alcohol consumption. Laboratory results show elevated aspartate aminotransferase, probable mild cardiomegaly on chest x-ray, and MRI findings are provided without specific interpretation.	Diagnosis	Substance-induced sleep disorder
			Disease characteristics	All of the above
Case 422	Psychiatry	A 26-year-old man presents with episodes of night screaming, sweating, agitation, recurrent nightmares, hypervigilance, and significant distress, with physical examination revealing elevated respiration rate, tachycardia, hypertension, minor tremulousness, hyperhidrosis, and an anxious, agitated affect. Laboratory and imaging results show a heart rate of 107 beats/min on ECG, negative urine toxicology, normal urinalysis, and complete blood cell count, comprehensive metabolic profile, and thyroid-stimulating hormone levels within reference range.	Diagnosis	Posttraumatic stress disorder
			Disease characteristics	A 34-year-old man who has been sexually assaulted by inmates in a prison
			Diagnosis	Lack of direct personal exposure to physical, sexual, or witnessed violence
Case 423	Paediatrics	A 19-month-old female presents with progressive pubic hair development, aggressive behavior, muscular arms and legs, clitoromegaly, and advanced bone age of 2.5 years. Laboratory results show elevated serum 17-hydroxypregnenolone, DHEAS, DHEA, androstenedione, and testosterone levels.	Diagnosis	Androgen-secreting adrenocortical tumor
			Examination	In addition to androgen hormones, an adrenocortical tumor may also secrete excessive cortisol, leading to clinical symptoms of Cushing syndrome
			Disease characteristics	Abdominal and pelvic ultrasound
Case 424	Minor	A 5-year-old girl with severe asthma and eczema presents with rapid weight gain, excessive daytime sleepiness, inattention, and enlarged tonsils, along with a family history of obesity, obstructive sleep apnea, and narcolepsy. Polysomnography reveals a respiratory disturbance index and apnea-hypopnea index of 6.2 per hour, sleep latency of 0.5 minutes, REM latency of 0.5 minutes, sleep efficiency of 87.5%, and an arousal index of 18.6.	Diagnosis	Narcolepsy
			Disease characteristics	Narcolepsy is associated with the loss of orexin neuropeptides
			Treatment	Modafinil as a daytime stimulant, fluoxetine for cataplexy, and sodium oxybate to improve REM efficiency
Case 425	Internal Medicine	A 42-year-old man presents with altered mental status, seizures, fever, lethargy, and a flat affect, with physical examination showing no nuchal rigidity or rashes. Laboratory and imaging results reveal bilateral central ground-glass opacities on chest X-ray, diffuse leptomeningeal enhancement with cerebral edema on brain MRI, negative HIV test, normal blood counts, elevated CSF nucleated cell count with lymphocytic predominance, elevated protein, normal glucose, negative Gram stain and culture, and elevated CSF beta-(1,3)-D-glucan, leading to concerns for tuberculous meningitis and fungal infection.	Diagnosis	Coccidioides immitis
			Examination	Positive CSF Coccidioides antibodies
			Treatment	Itraconazole
Case 426	Internal Medicine	A 46-year-old man presents with rectal bleeding, pelvic pain, constipation, abdominal distension, and moderate tenderness and rigidity in the iliac fossae, with a history of ulcerative colitis and primary sclerosing cholangitis. Laboratory results show anemia, leukocytosis, thrombocytopenia, elevated carcinoembryonic antigen, bilirubin, gamma-glutamyl transferase, alkaline phosphatase, fecal calprotectin, and C-reactive protein, while imaging reveals dilation of the extrabiliary ducts and a pseudokidney structure in the left iliac fossa suggestive of colon cancer.	Diagnosis	Colon cancer
			Disease characteristics	Sustained remission, whatever the regimen
			Disease characteristics	Colorectal cancer is associated with a longer duration of PSC
Case 427	Internal Medicine	A 47-year-old man presents with severe abdominal pain, inability to pass stool or flatus, fever, fatigue, weight loss, generalized lymphadenopathy, tender splenomegaly, and abdominal tenderness with guarding and rigidity. Laboratory results show anemia, leukocytosis, neutropenia, elevated ferritin, and LDH, while imaging reveals an abdominal lesion.	Diagnosis	Lymphoma
			Disease characteristics	Minute perforation associated with lymphoma, with bacterial translocation
			Disease characteristics	Thiopurine therapy
Case 428	Internal Medicine	A 21-year-old man presents with recurrent mild abdominal discomfort, weakness, weight loss, occasional diarrhea, anal itching, and dark stool,	Diagnosis	Taenia saginata
			Disease characteristics	Pallor

		appearing apprehensive and mildly pale on examination. Laboratory results show a hematocrit of 26%, an eosinophil count of 0.5×10⁹/L, normal abdominal ultrasonography, and stool microscopy revealing small, white, rice-like bits.	Examination	Stool microscopy
Case 429	Internal Medicine	A 25-year-old graduate student presents with acute left heel pain, a history of a stress fracture in her femur, missed menstrual periods, and physical examination reveals she is thin with a heart rate of 41 beats/min and normal skin, heart, respiratory, and neurologic findings. Laboratory tests show a hematocrit of 35% and hemoglobin of 11 g/dL, while imaging reveals a nondisplaced calcaneal fracture and low bone density.	Diagnosis	Stress fracture due to osteopenia
			Disease characteristics	Osteoporosis and osteopenia
			Treatment	Transdermal estrogen
Case 430	Internal Medicine	A 45-year-old man presents with painless bilateral ankle swelling, recurrent fevers, productive cough with blood-tinged sputum, anorexia, weight loss, recurrent epistaxis, saddle nose deformity, and bilateral conductive hearing loss, with physical examination revealing cachexia, pallor, and scattered coarse breath sounds. Laboratory findings show moderate normochromic normocytic anemia, elevated ESR, elevated serum creatinine, low serum albumin, significant proteinuria, and Pseudomonas aeruginosa in sputum, while imaging reveals multiple cavitary lung lesions and moderate hydronephrosis on abdominal ultrasound.	Diagnosis	Granulomatosis with polyangiitis (Wegener granulomatosis)
			Treatment	High serum creatinine level
			Examination	Azathioprine
Case 431	Paediatrics	A 5-year-old boy presents with recurrent ear infections, fever, purulent ear drainage, and a history of recurrent bacterial pneumonia, with physical examination showing a slim build, erythematous left tympanic membrane with purulent drainage, and palpable nontender cervical lymph nodes. Laboratory results reveal a normal white blood cell count, low IgG level, normal IgA and IgM levels, and extremely high titers to some pneumococcal serotypes but lacking protective titers to others.	Diagnosis	Common variable immunodeficiency (CVID)
			Disease characteristics	Pneumococcus
			Disease characteristics	Meningococcus
Case 432	Internal Medicine	A 30-year-old man presents with recurrent syncope, dizziness, nausea, and a forehead bruise, with stable vital signs and an unremarkable physical and neurologic examination. Laboratory tests are unremarkable, and the ECG shows a pattern suggestive of Brugada syndrome.	Diagnosis	Brugada syndrome
			Examination	History of syncopal episodes
			Diagnosis	Downsloping ST-segment elevations in V1-V3
Case 433	Internal Medicine	A 32-year-old woman with a history of severe mitral stenosis presents with acute respiratory distress, tachypnea, labored breathing, bilateral crackles, dullness to percussion at lung bases, and an irregularly irregular tachycardic heart rhythm with a holosystolic murmur. Laboratory and imaging results show pulmonary edema on chest X-ray with a normal-sized heart, and an ECG indicating an abnormal rhythm with a rate of 140 beats/min.	Diagnosis	New-onset atrial fibrillation
			Examination	5
			Treatment	Warfarin
Case 434	Internal Medicine	A 56-year-old woman presents with swollen ears, a facial rash, and right shoulder pain, with physical examination revealing edema of the right ear cartilage and limited range of motion in the right shoulder. Laboratory results show a positive ANA titer of 1:2560 with a speckled pattern, positive rheumatoid factor, positive anti-Ro and anti-La antibodies, elevated ESR, and normal imaging findings, while biopsies reveal lymphoid infiltrates without evidence of chronic T-cell lymphoma.	Diagnosis	Sjögren syndrome
			Examination	Decreased C3 and C4 levels
			Examination	Lymphohistiocytic inflammatory infiltrates and significant mucin deposition
Case 435	Minor	A 66-year-old man presents with a 2-year history of a nonhealing scalp wound, occasional scalp blistering, and tense bullae on the abdomen and forearms, with a physical exam showing a bright red eroded plaque on the scalp and scarring alopecia. A punch biopsy reveals subdermal blistering, eosinophil-rich inflammatory infiltrate, and positive direct immunofluorescence with	Diagnosis	Brunsting-Perry cicatricial pemphigoid
			Disease characteristics	IL-4 and IL-13 are cytokines involved in the inflammatory response; inhibiting their effect will help improve autoimmune reactions

		linear IgG and C3 deposits along the basement membrane.	Treatment	The primary areas of involvement are generally the head and neck
Case 436	Minor	A 77-year-old man with a history of prostate cancer presents with a nonpruritic, nontender maculopapular scrotal rash, prominent jugular venous pulse, faint crackles in the lower lung lobes, a 4/6 systolic murmur, and grade 2 edema. Laboratory results show a PSA level of 155 ng/dL and elevated alkaline phosphatase at 238 U/L, while imaging reveals bilateral lung infiltrates and rib and spine metastasis.	Diagnosis	Cutaneous metastatic prostatic adenocarcinoma
			Disease characteristics	Bone
			Disease characteristics	Spinal cord compression
Case 437	Minor	A 16-year-old girl presents with acute severe headache, nausea, vomiting, intermittent diplopia, dizziness, blurred vision, photophobia, papilledema, venous engorgement, bilateral sixth cranial nerve palsy, and a generalized tonic-clonic seizure, with elevated blood pressure and bilateral extensor plantar reflexes. CT imaging reveals signs consistent with increased intracranial pressure.	Diagnosis	Colloid cyst
			Disease characteristics	All of the above
			Treatment	It is the most common third ventricular mass in adult patients
Case 438	Internal Medicine	A 42-year-old man presents in a comatose state with a Glasgow Coma Scale score of 6, bilateral lower extremity pitting edema, abdominal distension with ascites, and icteric sclerae, following two episodes of loss of consciousness with urinary incontinence and myoclonic jerks. Laboratory results show anemia, thrombocytopenia, hyponatremia, hypokalemia, hyperbilirubinemia, elevated ammonium, hypomagnesemia, and hypocalcemia, with a brain CT scan negative for acute abnormalities.	Diagnosis	Torsade de pointes
			Disease characteristics	A 38-year-old man with a history of schizophrenia recently treated with erythromycin for a sinus infection
			Treatment	Intravenous magnesium sulfate
Case 439	Internal Medicine	A 28-year-old woman presents with severe upper right abdominal pain radiating to the epigastrium, slight jaundice, and mild pruritus, with physical examination showing yellow sclerae and moderate right hypochondrial tenderness. Laboratory results reveal anemia, thrombocytopenia, leukopenia, elevated liver enzymes, and hyperbilirubinemia, while imaging shows a large hyperechoic liver lesion with peripheral vascularity.	Diagnosis	Hemangioma
			Disease characteristics	Left renal vein
			Disease characteristics	Incidental detection
			Treatment	Radiofrequency ablation
Case 440	Internal Medicine	A 60-year-old man presents with hematemesis, progressive dyspepsia, early satiety, severe colicky abdominal pain, and significant weight loss, with physical examination showing epigastric and periumbilical tenderness. Laboratory results reveal anemia with hemoglobin at 8 g/dL, low mean corpuscular volume, elevated LDH, ESR, creatinine, BUN, sodium, and CA 19-9 levels, while imaging shows illuminated gut and abdominal lymph nodes on PET, and circumferential gastric wall thickening on CT, with biopsies positive for CD117 and CD34 markers.	Diagnosis	Gastrointestinal stromal tumor with nodal metastasis
			Disease characteristics	GIST affects the stomach more frequently than the intestine
			Treatment	MALT lymphoma
Case 441	Internal Medicine	A 75-year-old man presents with severe cough, expectoration, fever, sweating, chills, progressive dysphagia, regurgitation, vomiting, weight loss, dehydration, epigastric tenderness, and scattered chest crepitations. Laboratory results show anemia, thrombocytopenia, elevated ESR, hypernatremia, hypokalemia, elevated creatinine and BUN, elevated liver enzymes, and high CEA, while imaging reveals esophageal stricture with candidal infection and severe bronchitis.	Diagnosis	Esophageal cancer
			Treatment	Endoscopic mucosal resection
			Disease characteristics	Liver
Case 442	Internal Medicine	A 50-year-old woman presents with necrotic ulceration of her extremities, gangrenous digits, and right wrist and left-sided foot drop, with physical examination showing no mucous membrane involvement and deformities associated with long-standing rheumatoid arthritis. Laboratory results reveal elevated rheumatoid factor and anti-CCP antibody levels, normocytic normochromic anemia, thrombocytosis, elevated ESR and CRP, with negative ANA, cryoglobulin, and vasculitis panel, and normal complement, liver, and kidney function tests.	Diagnosis	Rheumatoid vasculitis
			Diagnosis	Thromboangiitis obliterans
			Disease characteristics	Rheumatoid vasculitis

Case 443	Internal Medicine	A 50-year-old man presents with oral thrush, skin rash, significant weight loss, diarrhea, severe weakness, fever, malaise, shortness of breath, dry cough, intermittent headaches, neck stiffness, cachexia, and widespread papular rash with some umbilicated lesions, along with nuchal rigidity, cervical lymphadenopathy, and bilateral crackles on lung auscultation. Laboratory tests show a positive HIV-1 antigen/antibody test, elevated lymphocytes, positive 1,3-beta-D-glucan test, and normal comprehensive metabolic panel, while imaging reveals lymphadenopathy, bilateral interstitial infiltrates, multiple pulmonary nodules, small pleural effusions on chest radiograph, and brain MRI shows lesions in	Diagnosis<br>Examination<br>Treatment	Disseminated Cryptococcus infection<br>Clinical improvement after therapy<br>Prozone effect
Case 444	Minor	A 67-year-old woman presents with severe right-sided weakness, difficulty speaking, right-sided facial droop, decreased muscle tone, and a positive Babinski sign on the right, along with conjugate eye deviation to the left and global aphasia. Laboratory analyses are normal, and a noncontrast cerebral CT scan is performed.	Diagnosis<br>Disease characteristics<br>Disease characteristics	Left hemispheric ischemic stroke<br>Aphasia<br>Hypoglycemia
Case 445	Minor	A 47-year-old man with HIV presents with a foreign-body sensation, periorbital edema, erythema, hemorrhagic and edematous sclera, cloudy cornea with an ulcer, and thick yellow discharge in the right eye, along with a right-sided headache. Laboratory results show a white blood cell count of 12,000 cells/μL, hemoglobin level of 12.5 g/dL, and a right corneal ulcer near rupture, leading to an urgent corneal transplant.	Diagnosis<br>Examination<br>Treatment	Infectious keratitis (bacterial)<br>Ceftriaxone<br>NAAT
Case 446	Major Surgery	A 52-year-old obese woman presents with shortness of breath, crampy abdominal pain, mild dyspnea, crackles at the right lung base, and a benign abdominal examination. Laboratory results show hemoglobin of 9.1 g/dL, albumin of 2.3 g/dL, sodium of 131 mEq/L, potassium of 3.3 mEq/L, with imaging revealing a right pleural effusion, ascites, omental caking, and a 7-cm right pelvic mass.	Diagnosis<br>Treatment<br>Treatment	Ovarian cancer<br>Cisplatin IP and paclitaxel IV/IP<br>Referral to a gynecologic oncologist for further evaluation
Case 447	Minor	A 69-year-old woman with a history of myelodysplastic syndrome, neutropenia, COPD, and cirrhosis presents with neutropenic fever, painful skin lesions, shortness of breath, and tachypnea, with physical examination revealing erythematous, edematous plaques on her back and calf, and tender papules on her chest. Laboratory findings show pancytopenia with a WBC count of 1.5×10^9/L, ANC of 0.3×10^9/L, platelet count of 10 $\times10^9$/L, hemoglobin level of 6 g/dL, unremarkable chest radiograph, and biopsy showing mild spongiosis, ac	Diagnosis<br>Diagnosis<br>Treatment	Sweet syndrome<br>Oral prednisone<br>Abrupt onset of painful erythematous plaques or nodules
Case 448	Minor	An 80-year-old man presents with drowsiness, confusion, dehydration, mild epigastric tenderness, and develops difficulty swallowing, slurred speech, and spastic quadriplegia. Laboratory results show severe hyponatremia with sodium at 99 mEq/L, and MRI reveals high signal-intensity areas in the central pons on T2/FLAIR sequences.	Diagnosis<br>Disease characteristics<br>Examination	Central pontine myelinolysis<br>Rapid correction of hyponatremia<br>MRI of the brain
Case 449	Minor	A 73-year-old man presents with a 3-day history of mild headache, somnolence, and vague, tangential responses, with physical examination showing slight hyperreflexia but otherwise normal findings. Laboratory results reveal hyponatremia with a sodium level of 129 mEq/L, and a CT scan of the head shows a right-sided hypodense-to-isodense lesion, signs of increased intracranial pressure, and midline shift.	Diagnosis<br>Diagnosis<br>Disease characteristics	Subacute/chronic subdural hematoma<br>Acute, subacute, and chronic subdural hematoma<br>Prompt surgical drainage improves outcome for hematomas of this size
Case 450	Psychiatry	An 18-year-old college student presents with academic struggles, difficulty focusing, frequent blinking, and compulsive behaviors, with a history of concussions and a family history of similar issues. Physical examination is normal except for anxious affect and repeated blinking, while laboratory tests and urine toxicology are normal, and CT imaging shows findings similar to a previous concussion case.	Diagnosis<br>Treatment<br>Disease characteristics	Obsessive-compulsive disorder<br>Sertraline<br>Maternal smoking during pregnancy
Case 451			Diagnosis	Statin toxicity

	Internal Medicine	A 60-year-old man with chronic tophaceous gout, coronary artery disease, and chronic kidney disease presents with multiple tophi, slight knee instability, and mild pedal edema. Laboratory results show elevated creatinine at 5.9 mg/dL after taking celecoxib daily instead of every other day, with normal electrolytes, calcium, and elevated phosphorus, and urinalysis revealing microscopic hematuria without RBCs; renal function returns to baseline after discontinuation of celecoxib and initiation of a furosemide regimen.	Examination	Low-dose colchicine or a low-dose NSAID with careful monitoring
			Treatment	Myoglobinemia
Case 452	Internal Medicine	A 62-year-old woman presents with diffuse lower abdominal discomfort, bloating, decreased appetite, weight loss, and shortness of breath, with physical examination revealing decreased breath sounds at the lung bases and evidence of abdominal fluid. Laboratory results show elevated CA-125 levels, normal other lab values, and imaging reveals small bilateral pleural effusions and a large volume of ascites on CT scan.	Diagnosis	Primary peritoneal carcinoma
			Treatment	High-dose chemotherapy with peripheral stem cell or bone marrow support
			Treatment	All of the above
Case 453	Internal Medicine	A 67-year-old man with COPD and atrial fibrillation presents with worsening dyspnea, fatigue, and dark stools, accompanied by tachycardia, pale and cold extremities, and weak peripheral pulses. Laboratory results show severe anemia with a hemoglobin level of 6.9 g/dL and a positive fecal occult blood test, while imaging includes an ECG with sinus tachycardia and an echocardiogram showing a normal ejection fraction, with endoscopic images revealing gastrointestinal bleeding.	Diagnosis	Acute gastrointestinal (GI) bleeding
			Treatment	De-escalate therapy to apixaban only
			Treatment	De-escalate therapy to clopidogrel and apixaban (dual therapy)
Case 454	Minor	A 50-year-old man presents with severe pain and progressive weakness in his left shoulder and arm, with physical examination revealing weakness in shoulder abduction, flexion, and external rotation, slight muscle wasting, and diminished sensation over the left deltoid and lateral forearm. Laboratory tests are normal except for glycosuria and elevated blood glucose, and cervical spine MRI results are provided.	Diagnosis	Brachial neuritis
			Disease characteristics	Brachial neuritis can be distinguished from rotator cuff syndrome on clinical grounds
			Treatment	Physical therapy is the mainstay of treatment for brachial neuritis after the painful stage
Case 455	Minor	A 46-year-old woman presents with sudden-onset blindness in her right eye, with physical examination revealing slight swelling and bruising around the glabella and a diffuse white pallor on the retina of the right eye. Laboratory tests show elevated fasting glucose and LDL cholesterol, while imaging reveals no cerebral ischemia or hemorrhage and intact blood flow to the cerebrum.	Diagnosis	Central retinal artery occlusion from dermal fillers
			Examination	Dilated funduscopic examination
			Treatment	Hyaluronidase introduced into the injection site and retrobulbar area
Case 456	Minor	A 33-year-old woman presents with a sudden severe headache, vomiting, mild blurred vision, and left leg weakness, with physical examination revealing bilateral papilledema and slightly sluggish pupillary reaction. Laboratory tests are normal, CT and MRI of the brain are normal, and a magnetic resonance venogram (MRV) is obtained.	Diagnosis	Cerebral venous thrombosis (CVT)
			Treatment	Heparin and warfarin
			Examination	MRV
Case 457	Minor	A 60-year-old woman presents with left-sided weakness, somnolence, severe occipital headache, episodic vomiting, feverish feeling, and positive Brudzinski sign, along with decreased muscle strength and brisk reflexes on the left side, left hemianesthesia, and left homonymous hemianopsia. Laboratory findings show elevated leukocyte count, elevated ESR, hyponatremia, and CSF analysis with elevated protein and lymphocytic pleocytosis, while a CT scan reveals a hypodense area in the right middle cerebral artery distribution consistent with cerebral infarction.	Diagnosis	Tuberculous meningitis with vasculitis
			Examination	Hypodense lesion with edema out of proportion to the mass effect on contrast-enhanced CT of the brain
			Examination	CSF ADA levels
Case 458	Internal Medicine	An 80-year-old woman presents with malaise, nausea, vomiting, abdominal pain, watery diarrhea, slight confusion, shortness of breath, icteric conjunctiva, and bilateral rales, with vital signs showing hypotension, tachypnea, tachycardia, and fever. Laboratory results reveal leukopenia, anemia, thrombocytopenia, elevated creatinine, urea, liver enzymes, bilirubin, lactate,	Diagnosis	Ehrlichiosis
			Disease characteristics	The lone star tick (Amblyomma americanum)
			Disease characteristics	Use of tick repellants such as DEET (N,N-diethyl-3-methylbenzamide) or permethrin

		ferritin, and triglycerides, while imaging shows diffuse bilateral interstitial and patchy air space opacities, moderate bilateral pleural effusions, and ground glass opacities on lung sections.		
Case 459	Internal Medicine	An 80-year-old man with COPD presents with sudden-onset right-sided chest pain, tachypnea, and a pansystolic murmur, with physical examination revealing absent breath sounds in the right upper and mid-lung zones and diffuse rhonchi. Laboratory results show chronic leukocytosis, normal cardiac enzymes, and a chest X-ray indicating bilateral airspace disease, a right lower-lobe nodule, and cardiomegaly, while a chest CT scan is performed for further evaluation.	Diagnosis	Pneumothorax
			Examination	Supine chest x-ray
			Treatment	Chemical pleurodesis via tube thoracostomy
Case 460	Minor	A 69-year-old woman presented with sudden right-cheek swelling, tenderness, mild difficulty swallowing, and a swollen, tender, mobile, and firm right parotid gland with purulent fluid upon pressure, while her face was asymmetrical and lower extremities showed pitting edema. Laboratory and imaging results are not provided in the case details.	Diagnosis	Bacterial (suppurative) parotitis
			Diagnosis	S aureus
			Treatment	Carbapenems
Case 461	Minor	A 40-year-old man presents with peripheral neuropathy, dry eyes and mouth, urinary retention, gastrointestinal symptoms, weight loss, decreased sweating, erectile dysfunction, and frequent syncopal episodes, with physical examination showing orthostatic hypotension, dusky-colored feet, reduced strength in lower extremities, decreased pin sensation below the mid-thigh, and mild foot drop. Laboratory tests, including blood work and antibody panels, were largely unremarkable except for a positive antinuclear antibody titer, while imaging and biopsy results showed reduced unmyelinated axons in the calf and moderate to severe sensorimotor axonal polyneuropathy, with no tumors found on CT.	Diagnosis	Hereditary amyloidosis due to transthyretin variant
			Disease characteristics	Orthostatic hypotension
			Examination	DNA analysis
Case 462	Internal Medicine	A 61-year-old woman presents with tachycardia, night sweats, significant weight loss, anxiety, diarrhea, occasional diplopia, bilateral exophthalmos, and a diffuse nontender goiter, with vital signs showing a pulse of 160 beats/min and blood pressure of 190/117 mm Hg. Laboratory studies show normal complete blood cell count, electrolytes, renal function, and cardiac markers, with normal chest radiography findings, and an ECG is obtained.	Diagnosis	Thyrotoxicosis
			Examination	PTU, propranolol, potassium iodide, dexamethasone, and acetaminophen
			Treatment	TSH
Case 463	Internal Medicine	A 36-year-old man presents with frequent vomiting, mild epigastric pain, dehydration, cachexia, and temporal wasting, with physical examination showing mild tenderness in the epigastrium and no acute distress. Laboratory tests are pending, chest radiography is normal, and abdominal CT reveals a large gastric mass causing gastric outlet obstruction.	Treatment	Begin potassium chloride replacement
			Examination	Decreased T-waves
			Treatment	Magnesium
Case 464	Paediatrics	A 10-month-old male infant presents with cough, difficulty breathing, significant respiratory distress, nasal flaring, retractions, elevated heart rate, and mild rales in the lower to mid lung fields. Laboratory and imaging results include an ECG with tachycardia and a chest radiograph showing bilateral perihilar infiltrates.	Diagnosis	Heart failure
			Examination	Hemodynamically stable SVT or VT can be treated medically in pediatric patients
			Treatment	The rhythm seen in myocarditis is most commonly sinus tachycardia
Case 465	Minor	A 56-year-old man with a history of kidney transplant and immunosuppressive therapy presents with a progressive rash on his chest, upper arms, buttocks, mid-back, and feet, accompanied by intermittent itching but no other systemic symptoms. Laboratory tests including complete blood cell count, comprehensive metabolic panel, and autoantibody measurements are within normal limits, and physical examination reveals no lymphadenopathy or other abnormalities.	Diagnosis	Extensive tinea corporis
			Treatment	Systemic antifungal therapy
			Disease characteristics	History of diabetes
Case 466	Minor	A 51-year-old man presents with an epidermal cyst on his left lower eyelid and two 8-mm pink macules with scale on his right arm, which blanch slightly with	Diagnosis	Superficial basal cell carcinoma
			Treatment	Vismodegib

		compression. The lesions are biopsied using the shave technique for pathologic evaluation.	Treatment	All of the above
Case 467	Major Surgery	A 29-year-old pregnant woman at 20 weeks' gestation presents with a 2-day history of fever, chills, abdominal pain, and serosanguinous vaginal discharge, with physical examination revealing a temperature of 102.9°F, heart rate of 139 beats/min, and blood pressure of 92/36 mm Hg. Laboratory results show anemia with a hemoglobin level of 9.7 g/dL, elevated lactic acid at 2.6 mmol/L, and low magnesium and phosphorus levels, while chest CT reveals bilateral ground-glass opacities, and PCR tests for COVID-19 and other respiratory viruses are negative.	Diagnosis	Fungal chorioamnionitis
			Treatment	Until delivery
			Treatment	Liposomal amphotericin B
Case 468	Minor	A 57-year-old man presents with left-sided weakness, slurred speech, right eye ptosis, and decreased sensation on the left side following violent coughing, with physical examination revealing marked left hemiparesis and pupillary asymmetry. Laboratory results are normal, chest X-ray is normal, ECG shows left ventricular hypertrophy, CT scan reveals a hyperdense right middle cerebral artery, and angiography shows stenosis at the origin of the right internal carotid artery.	Diagnosis	Right internal carotid artery dissection
			Examination	Intravenous heparin followed by oral anticoagulation for 3-6 months with periodic MRI/MRA
			Treatment	MRI/MRA with fat-suppressed T1 images
Case 469	Minor	A 24-year-old man presents with progressive lower extremity weakness, numbness, tingling, and incontinence, with physical examination showing decreased strength, impaired sensation, and absent deep tendon reflexes in the lower extremities. Laboratory results reveal macrocytic anemia with elevated homocysteine and methylmalonic acid levels, low-normal vitamin B12, and a normal brain CT scan.	Diagnosis	Subacute combined degeneration of the spinal cord
			Treatment	Spinocerebellar tract, lateral corticospinal tract, dorsal columns
			Disease characteristics	Vitamin B12 supplementation
Case 470	Internal Medicine	A 70-year-old man presents with a rapidly growing, nontender mass in the left medial epitrochlear region, with normal overlying skin and no systemic symptoms. CT imaging reveals a 4.0 cm ×3.1 cm soft tissue density exerting mass effect on the basilic vein, with surgical pathology showing a gelatinous pink-gray tissue mass.	Diagnosis	Diffuse large B-cell lymphoma
			Disease characteristics	PET
			Examination	BCL2 and BCL6
Case 471	Internal Medicine	A 30-year-old woman presents with recurrent abdominal pain, loose stools, and bloating, with physical examination showing a soft, non-distended abdomen and brown, guaiac-negative stool. Laboratory tests, including complete blood cell count and basic metabolic profile, are within the reference range.	Diagnosis	Irritable bowel syndrome
			Examination	Establish a therapeutic clinician/patient relationship to validate the patient's symptoms
			Treatment	Loperamide
Case 472	Minor	A 50-year-old man presents with a 2-month history of a worsening wound on his left foot, characterized by edematous, macerated, yellow-crusted plaques and significant tenderness, along with thickened and dystrophic nails. Laboratory findings reveal hyphae on potassium hydroxide preparation and swabs growing Pseudomonas aeruginosa.	Diagnosis	Mixed toe web infection
			Disease characteristics	Fungal infection
			Treatment	Oral antibiotics
Case 473	Minor	A 45-year-old woman with rheumatoid arthritis presents with right hand weakness, difficulty with buttoning and writing, and physical examination reveals swelling and tenderness of the metacarpophalangeal joints, limited range of motion in the right elbow and shoulder, and weaker extension of the right wrist, fingers, and thumb. Laboratory tests show an elevated erythrocyte sedimentation rate, and a plain radiograph of the right wrist is normal.	Diagnosis	Posterior interosseous nerve syndrome
			Disease characteristics	Electrodiagnostic studies
			Examination	Extensor digitorum communis and extensor pollicis longus
Case 474	Minor	A 31-year-old woman presents with progressive right wrist pain, particularly intense when lifting her child, with mild soft-tissue swelling near the radial styloid and exquisite pain on the Finkelstein maneuver. Laboratory results show a mildly elevated ESR with normal CBC, rheumatoid factor, and ANA, while radiographs show no bony abnormalities and MRI findings are not specified.	Diagnosis	de Quervain tenosynovitis
			Disease characteristics	Pain with use of the thumb
			Disease characteristics	The pain typically occurs with repetitive lifting and radial deviation at the wrist
Case 475	Internal Medicine	A 72-year-old woman presents with severe exhaustion, fatigue, hypertension, poorly controlled diabetes, paroxysmal atrial fibrillation, and physical examination reveals frailty, a pansystolic murmur, and nodularity in	Diagnosis	Obstructive sleep apnea
			Disease characteristics	Mandibular advancement splint
			Treatment	Apnea-hypopnea index

		the oral cavity with bony masses. Laboratory results show elevated hemoglobin and hematocrit, high serum bicarbonate, normal thyroid function, and CT imaging reveals multiple exostotic bony lesions in the maxilla, mandible, and oral cavity floor.		
Case 476	Minor	A 17-year-old girl presents with progressive clumsiness, dystonia, speech difficulty, mild jaundice, icteric sclera, dark rings around the iris, slow mentation, slurred speech, ataxic gait, diffuse muscle rigidity, and a fine resting tremor, with an enlarged, firm liver. Laboratory results show elevated bilirubin, alanine aminotransferase, and alkaline phosphatase levels, prolonged prothrombin time, negative hepatitis markers, and brain MRI findings consistent with Wilson disease.	Diagnosis	Wilson disease
			Disease characteristics	Chelation with penicillamine
			Treatment	Although KF rings are diagnostically useful, they are no longer considered pathognomonic for Wilson disease

Table S2. Comparison of Medscape respondents and GPT model responses

(a) Comparison of Medscape respondents (majority vote) and GPT-4o responses

		GPT-4o		Total
		Correct	Wrong	
Medscape respondents	Correct	1124	88	1212
	Wrong	137	77	214
Total		1261	165	1426

(b) Comparison of Medscape respondents (majority vote) and o1 responses

		o1		Total
		Correct	Wrong	
Medscape respondents	Correct	1176	36	1212
	Wrong	169	45	214
Total		1345	81	1426

Table S3. Performance by Option Count

Option count	No. of questions	Accuracy (95% CI, n)					Odds Ratio[a]	Adjusted *P* value[b]
		Medscape respondents	GPT					
			3.5 Turbo	4 Turbo	4 Omni	o1		
2-3	2	100.0 (15.8–100.0, 2)	50.0 (1.3–98.7, 1)	50.0 (1.3–98.7, 1)	100.0 (15.8–100.0, 2)	100.0 (15.8–100.0, 2)	–	–
4	906	86.1 (83.7–88.3, 780)	60.8 (57.6–64.0, 551)	83.4 (80.9–85.8, 756)	89.6 (87.5–91.5, 812)	95.0 (93.4–96.4, 861)	3.52 (2.41–5.14)	**< .001**
5	480	84.4 (80.8–87.5, 405)	61.7 (57.2–66.0, 296)	81.0 (77.2–84.5, 389)	87.7 (84.4–90.5, 421)	93.8 (91.2–95.7, 450)	3.57 (2.12–6.01)	**< .001**
≥6	38	65.8 (48.7–80.4, 25)	42.1 (26.3–59.2, 16)	63.2 (46.0–78.2, 24)	68.4 (51.4–82.5, 26)	84.2 (68.8–94.0, 32)	4.68 (0.98–22.4)	0.054
Total	1426	85.0 (83.1–86.8, 1212)	60.6 (58.1–63.1, 864)	82.1 (80.1–84.0, 1170)	88.4 (86.8–90.1, 1261)	94.3 (93.1–95.5, 1345)	3.20 (2.42–4.23)	–

[a] o1 relative to Medscape respondents

[b] Medscape respondents vs. o1, *P* value adjusted using the Holm–Bonferroni correction

CI, confidence interval

Table S4. Performance by Image Modality

Image Modality	Presence	No.of questions (% of total)	No.of correct responses (Accuracy)		
			Medscape respondents	GPT	
				4 Omni	o1
Radiography	Yes	164 (17.9 %)	135 (82.3 %)	138 (84.1 %)	147 (89.6 %)
	No	753 (82.1 %)	636 (84.5 %)	672 (89.2 %)	714 (94.8 %)
CT	Yes	221 (24.1 %)	188 (85.1 %)	198 (89.6 %)	208 (94.1 %)
	No	696 (75.9 %)	583 (83.8 %)	612 (87.9 %)	653 (93.8 %)
Sonography	Yes	58 (6.3 %)	51 (87.9 %)	50 (86.2 %)	53 (91.4 %)
	No	859 (93.7 %)	720 (83.8 %)	760 (88.5 %)	808 (94.1 %)
MRI	Yes	141 (15.4 %)	108 (76.6 %)	124 (87.9 %)	132 (93.6 %)
	No	776 (84.6 %)	663 (85.4 %)	686 (88.4 %)	729 (93.9 %)
Pathology Images	Yes	179 (19.5 %)	144 (80.4 %)	158 (88.3 %)	170 (95.0 %)
	No	738 (80.5 %)	627 (85.0 %)	652 (88.3 %)	691 (93.6 %)
Cutaneous /External findings	Yes	171 (18.6 %)	145 (84.8 %)	156 (91.2 %)	167 (97.7 %)
	No	746 (81.4 %)	626 (83.9 %)	654 (87.7 %)	694 (93.0 %)
ECG	Yes	79 (8.6 %)	70 (88.6 %)	73 (92.4 %)	75 (94.9 %)
	No	838 (91.4 %)	701 (83.7 %)	737 (87.9 %)	786 (93.8 %)
Endoscopy	Yes	77 (8.4 %)	57 (74.0 %)	66 (85.7 %)	71 (92.2 %)
	No	840 (91.6 %)	714 (85.0 %)	744 (88.6 %)	790 (94.0 %)
Angiography	Yes	21 (2.3 %)	18 (85.7 %)	20 (95.2 %)	21 (100.0 %)
	No	896 (97.7 %)	753 (84.0 %)	790 (88.2 %)	840 (93.8 %)
Others	Yes	148 (16.1 %)	119 (80.4 %)	129 (87.2 %)	140 (94.6 %)
	No	769 (83.9 %)	652 (84.8 %)	681 (88.6 %)	721 (93.8 %)
Total		917	771 (84.1 %)	810 (88.3 %)	861 (93.9 %)
